# Transcriptome Profiling of Huanglongbing (HLB) Tolerant and Susceptible *Citrus* Plants Reveals the Role of Basal Resistance in HLB Tolerance

**DOI:** 10.3389/fpls.2016.00933

**Published:** 2016-06-28

**Authors:** Yunsheng Wang, Lijuan Zhou, Xiaoyue Yu, Ed Stover, Feng Luo, Yongping Duan

**Affiliations:** ^1^College of Plant Protection, Hunan Agricultural UniversityChangsha, China; ^2^School of Computing, Clemson UniversityClemson, SC, USA; ^3^U.S. Horticultural Research Laboratory, Agricultural Research ServiceFort Pierce, FL, USA

**Keywords:** citrus, Huanglongbing, transcriptome, basal resistance

## Abstract

Huanglongbing (HLB) is currently the most destructive disease of citrus worldwide. Although there is no immune cultivar, field tolerance to HLB within citrus and citrus relatives has been observed at the USDA Picos farm at Ft. Pierce, Florida, where plants have been exposed to a very high level of HLB pressure since 2006. In this study, we used RNA-Seq to evaluate expression differences between two closely related cultivars after HLB infection: HLB-tolerant “Jackson” grapefruit-like-hybrid trees and HLB susceptible “Marsh” grapefruit trees. A total of 686 genes were differentially expressed (DE) between the two cultivars. Among them, 247 genes were up-expressed and 439 were down-expressed in tolerant citrus trees. We also identified a total of 619 genes with significant differential expression of alternative splicing isoforms between HLB tolerant and HLB susceptible citrus trees. We analyzed the functional categories of DE genes using two methods, and revealed that multiple pathways have been suppressed or activated in the HLB tolerant citrus trees, which lead to the activation of the basal resistance or immunity of citrus plants. We have experimentally verified the expressions of 14 up-expressed genes and 19 down-expressed genes on HLB-tolerant “Jackson” trees and HLB-susceptible “Marsh” trees using real time PCR. The results showed that the expression of most genes were in agreement with the RNA-Seq results. This study provided new insights into HLB-tolerance and useful guidance for breeding HLB-tolerant citrus in the future.

## Introduction

Huanglongbing (HLB), commonly known as citrus “greening”, is the most economically destructive disease of citrus worldwide. Since first identification of the disease in 2005 in Florida, HLB has spread throughout the state of Florida and has been found in other citrus producing states such as Texas and California. Because of its wide spread and lack of adequate control measures, Florida's 9 billion dollar citrus industry is presently fighting for its survival with an estimated $3.6 billion in lost revenues and more than 66,00 lost jobs from 2006 to 2014 (Hodges et al., 2014).

The HLB bacteria, including the prevalent species of “*Candidatus* Liberibacter asiaticus” (Las), reside in the phloem of the plant hosts and cause a systemic disease (Jagoueix et al., 1994). As an obligate and insect-transmitted plant pathogen, Las attacks all species and hybrids in the genus of *Citrus* (Halbert and Manjunath, 2004). Although there is no immune cultivar, some resistance or field tolerance to HLB within citrus and citrus relatives has been described (Miyakawa, 1980; Nariani, 1981; Miyakawa and Yuan, 1990; Halbert and Manjunath, 2004; Sharma et al., 2004). Compared to other tested cultivars within individual experiments, lower susceptibility to HLB associated with Las, has been reported for limes (Schwarz et al., 1973; Shokrollah et al., 2009), pummelos (Schwarz et al., 1973; Koizumi et al., 1997), lemons (Schwarz et al., 1973; Nariani, 1981; Cheema et al., 1982), some mandarin types (e.g., “Ladu” and “Som Pan” in Thailand, Koizumi et al., 1997) and various non-cultivated *Citrus* or related species.

**Figure 1:**
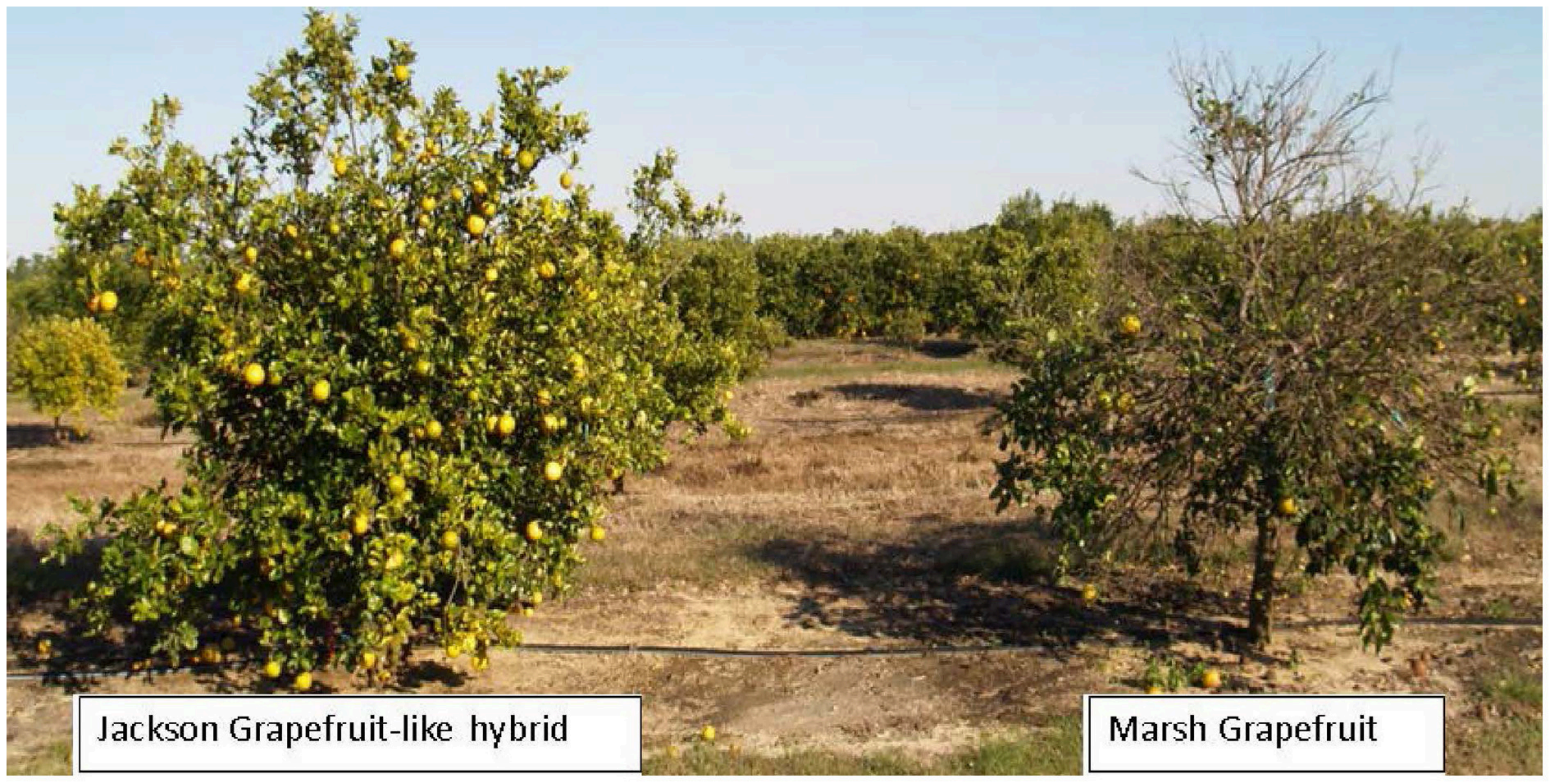
**Huanglongbing (HLB) tolerant “Jackson” and HLB susceptible “Marsh” grapefruit**. Picture was taken in 2010 but trees look similar in 2015.

The USDA citrus scion breeding program has been in existence for over 100 years and has an extraordinary diversity of materials under evaluation. Many of these unique trees are at the Ft. Pierce Picos farm of the USDA where plants have been exposed to a very high level of HLB pressure. Following 9 years of exposure to HLB, several entire hybrid populations including some cultivars show HLB tolerant with good growth and cropping despite the presence of HLB symptoms. One representative cultivar is the HLB tolerant “Jackson” which is considered a grapefruit but is a hybrid of the highly HLB-susceptible true grapefruit such as the cultivar “Marsh” (Figure 1). True grapefruit cultivars are hybrids between the sweet orange and the pummelo, and “Jackson” appears to be a further hybrid between grapefruit and sweet orange. Therefore, these two cultivars are very closely related, and are very similar in phenotype, but differ markedly in susceptibility to HLB (Stover et al., 2013). These similarities and differences provide an excellent opportunity to identify genes whose expression is associated with HLB tolerance.

Previously, both microarray and high-throughput sequencing technology were used to understand the global gene expressions of different tissues of citrus tree after HLB infection. Both Kim et al. (2009) and Mafra et al. (2013) used microarray to profile the gene expressions of sweet orange leaves after HLB infection. Liao and Burns (2012) used microarray and Martinelli et al. (2012) used RNA-Seq to understand the gene expressions of sweet orange fruit after HLB infection. Aritua et al. (2013) used microarray to study the gene expressions of both citrus stem and root in response to HLB infection. And Zhong et al. (2015) used RNA-Seq to analyze the gene expression of citrus root after HLB infection. Those studies showed some key pathways and processes, such as sugar and starch metabolism, cell wall metabolism, stress response, hormone signaling and phloem genes, were significantly altered in HLB affected citrus. However, the significantly altered genes were different in different tissues. By comparing the gene expression profiles of four types of tissue (immature fruits, mature fruits, young leaves, mature leaves) with four disease status (apparently healthy, asymptomatic, symptomatic and HLB-free control) using RNA-Seq, Martinelli et al. pointed out that the HLB symptoms would be mainly caused by source-sink disruption (Martinelli et al., 2013). To understand the citrus response to the HLB at system level, Zheng and Zhao compared the gene expression network of the previously reported microarray datasets, and found that some genes were commonly up-regulated due to HLB infection. Especially, they revealed a number of genes that were regulated specifically at different stage of the infection (Zheng and Zhao, 2013).

To best of our knowledge, there is no comparative study on transcriptome profiling between HLB tolerant and susceptible citrus cultivars. In this study, we profiled gene expression using RNA-Seq in two closely related cultivars of HLB tolerant and susceptible citrus trees under natural conditions. Our study was able to identify differentially expressed genes and revealed different responses to HLB infections in these two cultivars.

## Materials and methods

### Plant material and conformation of HLB infection

The transcriptome analysis was performed on three HLB tolerant “Jackson” grapefruit trees (R20T17, R20T18, and R19T17) and three HLB susceptible “Marsh” grapefruit trees (R19T23, R19T24, and R20T24). For expression validation of DE genes, we added two tolerant “Jackson” trees (R20T09 and R20T10) and two susceptible “Marsh” trees (R20T07 and R20T08) since these trees displayed similar phenotypes as the ones when we collected the flushes for RNA-Seq. All trees were growing in the USDA Picos farm at Ft. Pierce, Florida, where plants have been exposed to a very high level of HLB naturally for years, and the infection rate for these 10 year old grapefruit trees was 100% (Stover et al., 2013). To confirm HLB infection on these selected trees, leaf samples with typical HLB symptoms were collected. Genomic DNA was extracted using the DNeasy Plant Mini Kit (Qiagen, Valencia, CA) as described by Pitino et al. (2014). Real-time PCR based on the Las 16S rDNA was used for the titration of Las bacteria in these infected plants (Li et al., 2006). The bacterial titer sets were analyzed for statistical differences by Student's *t*-tests and shown no significant difference between two groups. Meanwhile, the young flush leaves were collected from each tree for RNA isolation and transcriptome analysis.

### RNA extraction and high throughput sequencing

The young leaves on each HLB tolerant or susceptible citrus plant were collected and frozen in liquid nitrogen using properly labeled tubes. Total RNA was extracted from whole leaves of each sample according to the RNeasy Plant Mini Kit standard protocol (Qiagen Inc., Valencia, CA). The quantity and quality of RNA was evaluated using Nanodrop ND-1000 spectrophotometer. A total of 20–30 μg RNA were sent to BGI-Hong Kong (China) for RNA sequencing.

We constructed the RNA-Seq libraries following the Illumina protocol of mRNA-sequencing sample preparation (Illumina Inc., San Diego, CA). The quality of each library was examined using a BioRad Experion (BioRad, Hercules, CA). The high-throughput sequencing was carried out by BGI using HiSeq2000 (Illumina, San Diego, CA).

### RNA-Seq data processing and analyzing

First, we cleaned the raw Illumina reads by removing low quality reads (Q20) and trimming adaptor sequences. Then, reads of each sample were mapped using Star (Dobin et al., 2013) with a maximum intron 5000 bp (–alignIntronMax 5000) to the *C. clementina* reference genome (Version 182) (Wu et al., 2014), which was downloaded from the Citrus Genome Database (http://www.citrusgenomedb.org/). The alignment bam files were sorted and indexed using SAMtools (Li et al., 2009). We counted the unique mapped reads for each gene using htseq-count from HTSeq (Anders et al., 2014).

We identified differentially expressed (DE) genes between HLB tolerant and HLB susceptible citrus trees using the DESeq (Anders and Huber, 2010) Bioconductor package. The raw counts of each gene were normalized to adjust for different sequencing depths across samples using DESeq. After estimating the dispersion of each gene, the DESeq identified the differentially expressed genes between HLB tolerant and HLB susceptible citrus trees using adjusted *p*-value (FDR) threshold 0.1.

### Transcriptome *De novo* assembly and reference genome annotation update

We merged all clean reads of the six samples into one big pseudo sample and assembled the merged clean reads using Trinity (Haas et al., 2013) with default parameters except that the “normalize reads” option was selected. Then, we mapped the assembled contigs to the reference genome using BLAT (Kent, 2002) and GMAP (Wu and Watanabe, 2005). We validated and combined the alignments using PASA (Haas et al., 2003). Then, with the transcriptome alignments, we used PASA to update the original genome annotation, such as adding UTRs, modifying the splicing, extending gene models, merging the spliced genes and detecting the isoform genes.

### Functional categorization and pathway analysis

We used Interproscan (Jones et al., 2014) to annotate the gene function and extract the GO terms of each gene. The GO enrichment analysis was carried out using topGO (Alexa et al., 2006) Bioconductor package with the *p*-value threshold of 0.01. The functionalities of differentially expressed genes were analyzed using MapMan (Urbanczyk-Wochniak et al., 2006) based on the pathways of Clementina_182 downloaded from the map server of MapMan. The pathways, which were affected by the differentially expressed genes, were analyzed using PageMan (Usadel et al., 2006) with WILCOXON as the summary statistic method.

### Protein-protein interaction network construction

We first predicted the citrus orthologs of *Arabidopsis* genes using BlastP (McGinnis and Madden, 2004) with *e*-value less than 1e-20. Then, we mapped the citrus orthologs onto the protein-protein interaction (PPI) network of Arabidopsis. The networks were identified and represented using Cytoscape software (Smoot et al., 2011).

### Transcript splicing analysis

Based on the updated citrus genome annotation from our RNA-Seq data, we compared the different splicing of each gene between HLB tolerant and HLB susceptible citrus trees using rMATS with junction reads only. rMATS (Shen et al., 2014) could detect differential occurrence of alternative splicing events, such as skipped exon (SE), alternative 5′ splice site (A5SS), alternative 3′ splice site (A3SS), mutually exclusive exons (MXE) and retained intron (RI) events, from replicate RNA-Seq data. We also detected the differential isoform usage of each genes between HLB tolerant and HLB susceptible citrus trees using IUTA (Niu et al., 2014).

### Real time PCR validation

Total RNA was extracted from tolerant and susceptible citrus leaves, respectively, with RNA extraction kits (Invitrogen, USA) according to the manufacturer's protocol. The RNA quality and quantity were detected with a Nanodrop spectrophoto-meter (Thermo Scientific, USA). Then 2 μg of RNA were treated with Turbo DNA-free DNase (Ambion, USA) to digest any residual DNA. The cDNA was synthesized using M-MLV Reverse Transcriptase (Invitrogen, USA) and an oligo (dT15) primer according to the manufacturer's protocol. The RT-PCR amplification were performed in an Eppendorf Mastercycler realplex thermal cycler (Eppendorf, Hauppauge, NY) using Fast SYBR Green Master Mix (Invitrogen, USA). The relative expression values were determined using the GADPH gene from citrus as reference gene and the comparative Ct method (2^−ΔΔCt^). The GADPH forward primer was: 5-GGAAGGTCAAGATCGGAATCAA-3; and reverse primer was: 5-CGTCCCTCTGCAAGATGACTCT -3. Each experiment was repeated three times.

## Results

### Transcriptome profiling using RNA-Seq

The transcriptome of young flush leaves from three HLB-tolerant “Jackson” (from here on, tolerant citrus refer to “Jackson”) grapefruit trees and three HLB-susceptible “Marsh” (from here on, susceptible citrus refer to “Marsh”) grapefruit trees were examined using the RNA-seq. Before the RNA-seq study, the real time PCR showed both susceptible and tolerant citrus trees were infected by HLB (Table 1). After cleaning the raw reads, we obtained more than 615 million clean reads in total, with more than 100 million reads for each of five samples and 72.9 million reads for one sample, R19T17 (Table 2). We mapped clean reads to the reference genome sequences of *Citrus clementina* (Wu et al., 2014) using the STAR (Dobin et al., 2013) program with a maximum intron space of 5 kb. We were able to map about 95% of the clean reads to the reference genome and more than 82% of the reads could be uniquely mapped to the reference genome (Table 2). Among those uniquely mapped reads, more than 80% of the reads were mapped to the exon regions. Meanwhile, 5.76–8.05% of the reads were mapped to the intronic regions and 9.67–11.5% of the reads were mapped to the intergenic regions (Figure S1). We have In total, we detected 22,497 *C. clementina* genes among all six samples and mapped RNA-Seq reads to 20,039–21,284 *C. clementina* genes.

**Table 1:** **Titers of *Candidatus* Liberibacter asiaticus in infected grapefruit plants**.

**Citrus varieties**	**Sample**	**Ct value**	**Las cell numbers/gram tissue**
Jackson-Tolerant	R19T17 R20T17 R20T18	28.78 ± 0.86a	331131
Marsh-Susceptible	R19T23 R19T24 R20T24	30.52 ± 1.29b	118304

**Table 2:** **RNA-Seq reads and mapping information**.

**Sample**	**Class**	**Total reads**	**Uniquely mapped reads**	**Number of mapped genes**
R19T23	S	106,378,986	94,591,386	21,284
R19T24	S	118,519,100	101,425,400	21,007
R20T24	S	101,960,380	86,943,256	21,070
R20T17	R	112,970,616	93,513,608	21,020
R20T18	R	102,649,394	89,659,798	21,137
R19T17	R	72,947,684	67,025,294	20,039

S, denoted HLB susceptible; R, denoted HLB tolerant.

### Updating citrus genome annotation using RNA-Seq reads

The citrus samples in our experiments have different pedigrees from the haploid *C. clementina* reference genome, but all are hybrids of the two species *C. reticulata* and *C. maxima*. However, there may be genes in our samples which do not exist in the *C. clementina* reference genome. Thus, we performed *de novo* assembly of genes using the RNA-seq reads to identify potential new genes. We combined all clean reads of the six samples together and assembled the transcripts using Trinity (Haas et al., 2013) with default parameters. We were able to obtain 241,048 contigs with more than 200 bp in length. The longest contig was 13,188 bases in length and the median length of contigs was 610 bp. Ninety percent of the contigs were less than 2 kb in length. The mean GC content of the assembled contigs was 0.39.

**Figure 2:**
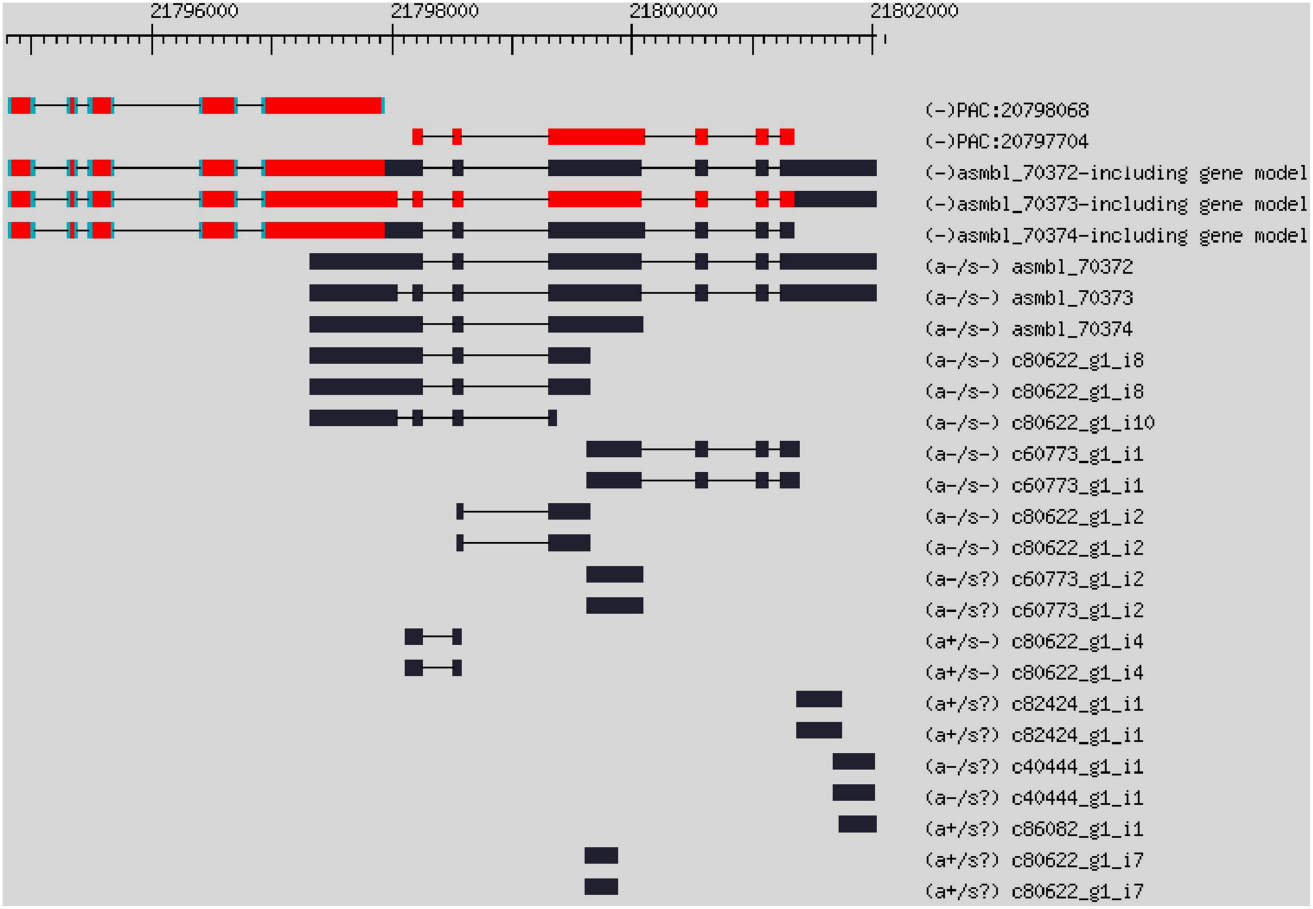
**Example of gene merging using RNA-Seq contigs**. Gene PAC:20798068 and PAC:10797704 were two genes in the original gene annotation, which can be merged into one gene with the evidence of RNA-Seq data.

Based on *de novo* assembled transcript contigs, we updated the citrus genome annotation using PASA (Haas et al., 2003). The PASA first aligned assembled transcripts to the *Citrus clementina* genome with BLAT (Kent, 2002) and GMAP (Wu and Watanabe, 2005). Out of the 241,048 assembled transcript contigs, 179,739 and 162,891 contigs were aligned by GMAP and BLAT, respectively. Together, there were 185,904 valid alignments in total. The PASA assembled the alignments into 97,479 assemblies. The PASA grouped the assemblies into 56,661 clusters. Each cluster corresponds to one gene. A cluster may have multiple assemblies due to splicing. Then, the PASA updated the citrus genome annotation by incorporating with the *C. clementina* reference genome (annotation version v182 downloaded from JGI). The PASA updated 14,902 UTR, 1712 gene extensions and 2075 genes for the original gene models. The PASA identified 519 new genes, which were absent from the original gene annotation (the gene model GFF file in Table S1). The PASA also identified 8441 alternative splicing isoforms from the RNA-Seq data. Some genes were split into multiple genes in the original JGI genome annotation. For example, with the evidence of RNA-Seq data, two genes PAC:20798068 and PAC:10797704 in the original JCI gene annotation can be merged into one gene (Figure 2). The PASA reported a total of 175 merged genes from 365 original genes.

### Identifying differentially expressed genes

Due to variation in sample preparation and sequencing procedures among different samples, the raw read count of genes cannot be compared directly. We first normalized the raw read counts of genes to remove the sequencing depth differences across samples. As shown in Figure S2, the variation of raw read counts of genes across different samples was high, and the normalization reduced the variations among different samples. Before normalization, the median read counts were from 361 to 445 among different samples. After normalization, the median read counts were from 381 to 416 among different samples.

Then, we used the DESeq (Anders and Huber, 2010) to identify differentially expressed (DE) genes between three HLB tolerant and three HLB susceptible citrus trees. We were able to identify 686 DE genes with adjusted *p*-value less than 0.1. The number of up-expressed genes in HLB susceptible “Marsh” trees was much greater than the number of genes down-expressed in HLB tolerant “Jackson” trees. In HLB susceptible “Marsh” trees 247 of 686 genes were up-expressed and 439 genes were down-expressed compared to HLB tolerant “Jackson” trees (Table S2). However, among top 20 significant DE genes (with the lowest adjusted *p*-values), 14 genes were up-expressed in HLB tolerant “Jackson” trees.

### Comparing alternative splicing of genes in HLB tolerant and susceptible citrus

In eukaryotes, a gene may produce multiple mRNA and protein isoforms through alternative combinations of exons during splicing. Alternative splicing is an essential method for eukaryotes to increase functional genes. We applied two complementary methods to identify alternative splicing difference between HLB tolerant and susceptible citrus trees. First, we compared the transcript splicing event difference using rMATS (Shen et al., 2014). Table 3 summarized five alternative splicing events and details of significantly differential alternative splicing events which are listed in Table S3. We have identified 134 significantly differential skipped exon (SE) events with FDR threshold 0.05. Among 134 events, 79 were in HLB susceptible “Marsh” trees and 55 were in HLB tolerant “Jackson” trees. We have identified 36 significantly differential MXE events. Both HLB tolerant and susceptible cultivar trees had 18 significant MXE events. Meanwhile, both HLB tolerant and susceptible cultivar trees had 32 alternative 5′ splice site (A5SS) events and similar numbers of alternative 3′ splice site (A3SS) events (36 for HLB tolerant “Jackson” trees and 43 for HLB susceptible “Marsh” trees). The HLB tolerant “Jackson” trees had 515 retained intron (RI) events, which were significantly higher than the 201 RI events that HLB susceptible “Marsh” trees had.

**Table 3:** **Differential alternative splicing events between HLB tolerant “Jackson” citrus trees compared to HLB susceptible “Marsh” trees**.

**ASE**	**Number of differential ASE**	**Number of ASE observed to be significant in S group**	**Number ASE observed to be significant in R group**
SE	134	79	55
MXE	36	18	18
A5SS	64	32	32
A3SS	78	36	42
RI	716	201	515

ASE, alternative splicing events; SE, skipped exon; MXE, mutually exclusive exon; A5SS, alternative 5′ splice site; A3SS, alternative 3′ splice site; RI, retained intron; S, HLB susceptible; R, HLB tolerant.

**Figure 3:**
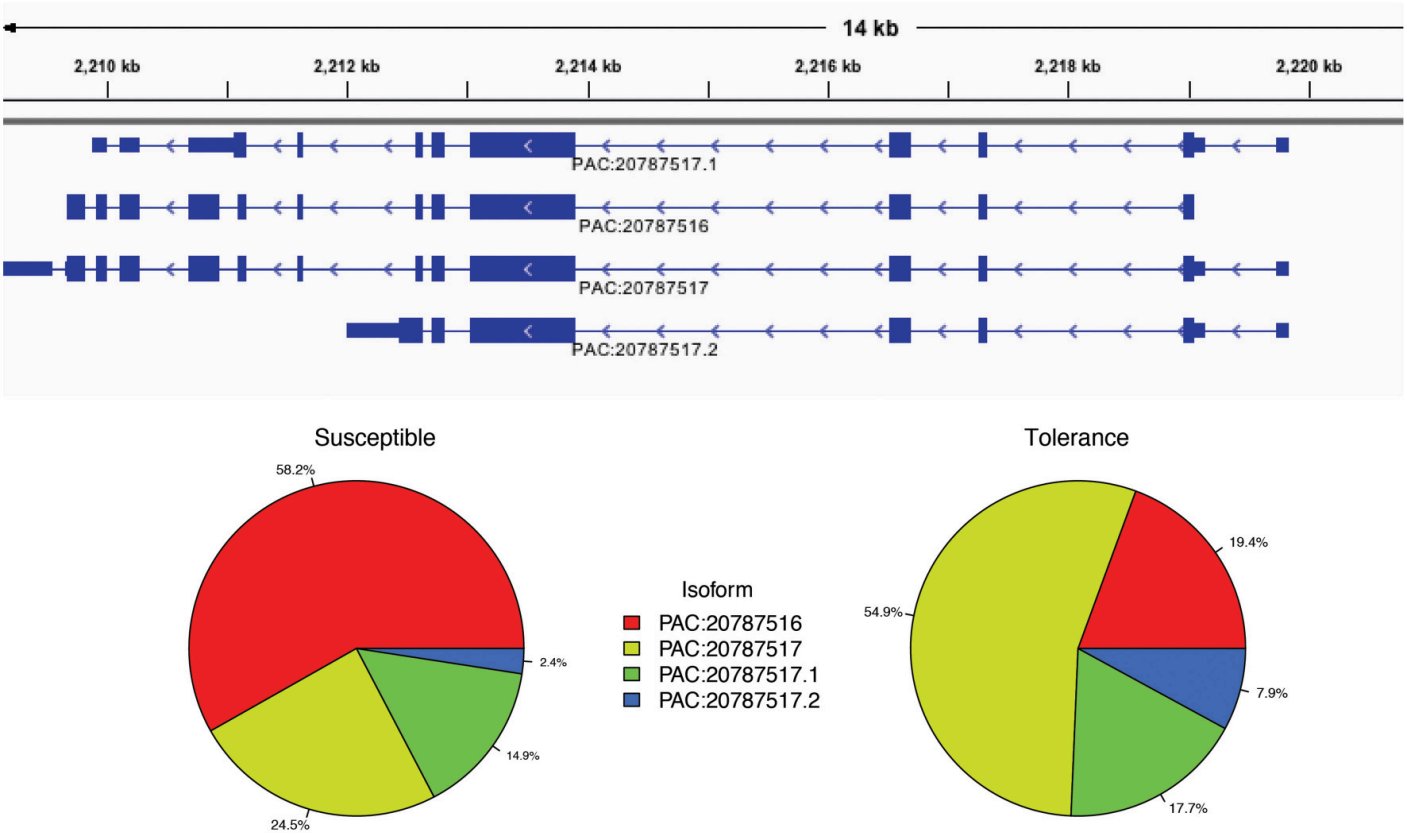
**Different isoform usages of gene Ciclev10000408m between HLB tolerant “Jackson” and HLB susceptible “Marsh” trees**. The most abundant isoform in HLB susceptible citrus trees is PAC:20787516 while the dominant isoform in HLB tolerant citrus trees is PAC:20787517.

We then used the IUTA (Niu et al., 2014) to detect differential isoform usage of each gene. There were 8,218 genes with alternative splicing events in the reference *C. clementina* genome with a total of 26,493 isoform transcripts. The median number of splicing isoform was 3 and the maximum was 35. Using the IUTA R package, we identified a total of 619 genes with significantly differential expression of alternative splicing isoforms between HLB tolerant “Jackson” and HLB susceptible “Marsh” trees (Table S4). For example, there were 4 splicing isoforms of gene Ciclev10000408m.g: PAC:20787516, PAC:20787517, PAC:20787517.1, and PAC:20787517.2. The most abundant isoform in HLB susceptible citrus trees was PAC:20787516 while the dominant isoform in HLB tolerant citrus trees was PAC:20787517 (Figure 3). Most of those genes showed no differential expression at gene level and only 14 genes were identified to be differentially expressed using DESeq with adjusted *p*-value less than 0.1.

### Functional analysis of RNA-Seq data

In order to understand the differential expressed genes between HLB tolerant “Jackson” and HLB susceptible “Marsh” trees, we analyzed the functionalities of DE genes using 2 methods. First, we examined the enriched functions in DE genes through GO enrichment analysis. We re-annotated the genes using InterProscan (Jones et al., 2014) and assigned the GO terms to each gene. Then, we used TopGO, which employed the Kolmogorov-Smirnov test and Fisher's exact test, to extract significantly enriched GO terms. For DE genes up-expressed in HLB tolerant “Jackson” trees, the most significantly enriched biological process GO terms were related to protein phosphorylation (GO:0006468, GO:0006464, GO:0036211, GO:0016310, GO:0006796, GO:0006793, GO:0043412) (Table S5). The other significantly enriched biological process GO terms were related to drug transmembrane transport, such as GO:0006855, GO:0015893, GO:0042493, GO:0042221. For DE genes up-expressed in HLB tolerant “Jackson” trees, the most significant molecular function GO terms were related to transferase activity (GO:0016740, GO:0016773, GO:0016301, GO:0004672, GO:0016772). The drug transporter activity (GO:0015238, GO:0090484) and oxidoreductase activity (GO:0016638, GO:0016641) were also up-expressed in HLB tolerant citrus trees. Only one cellular component GO term, cell periphery (GO:0071944), was enriched in DE genes up-expressed in HLB tolerant citrus trees.

On the other hand, in HLB susceptible citrus trees the most significant biological process GO terms in DE genes up-expressed were related to carbohydrate derivative catabolic processes (GO:1901136, GO:1901565). Biological processes related to chitin catabolism, such as GO:0006030, GO:0006032, were in DE genes up-expressed in HLB susceptible citrus. Another biological process category significantly DE genes up-expressed in HLB susceptible citrus was related to protein complex assembly, such as GO:0006461, GO:0070271, GO:0034622, GO:0065003. The most significant molecular function GO term in DE genes up-expressed in HLB susceptible citrus trees were related to chitinase activity (GO:0004568). Aspartic-type endopeptidase activity related GO items, such as GO:0004190, GO:0070001, were up-expressed in HLB susceptible citrus trees. The two cellular component GO terms enriched in DE genes up-expressed in HLB susceptible citrus trees are microtubule (GO:0005874) and protein-DNA complex (GO:0032993).

**Figure 4:**
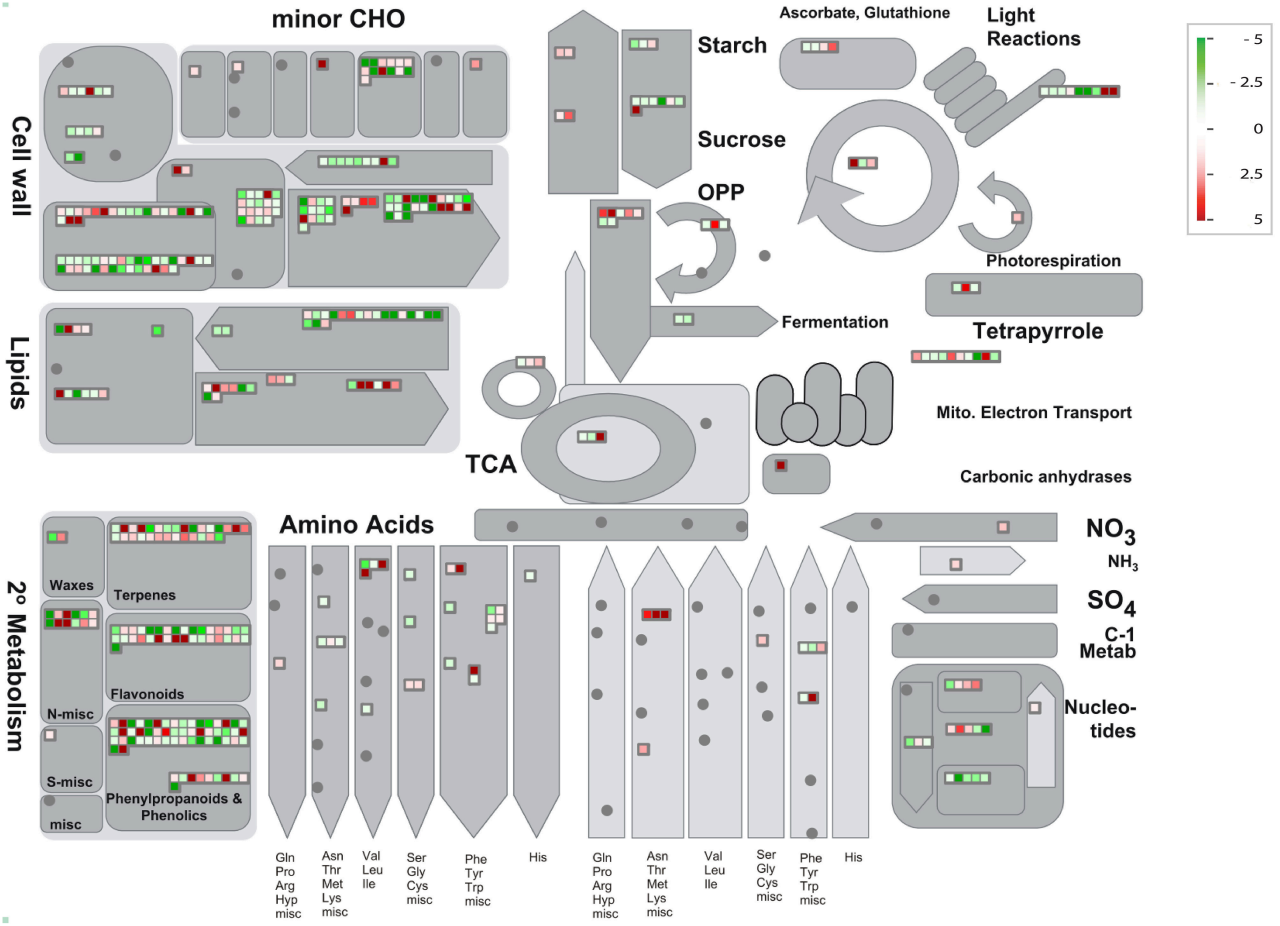
**Visualization of functional categories of differentially expressed genes between HLB tolerant “Jackson” and HLB susceptible “Marsh” trees**. Each colored square denoted a single annotated gene in a particular pathway. Color gradient between red (up-expressed in HLB tolerant “Jackson” trees compared to HLB susceptible “Marsh” trees) and green (down-expressed in HLB tolerant “Jackson” trees compared to HLB susceptible “Marsh” trees) represented the log fold ratios. Figure created by MapMan (Urbanczyk-Wochniak et al., 2006).

Then, we used PageMan (Usadel et al., 2006) to analyze gene functional categories that were differentially expressed in HLB tolerant and HLB susceptible citrus trees. We extended our study to whose average expressions with log2 based fold ratios >1 and <-1 between HLB tolerant and HLB susceptible citrus trees. The PageMan can pinpoint up-expressed and down-expressed genes onto different metabolic and cell function pathways. As shown in Table S6, gene expressions in the HLB tolerant citrus trees were increased in biotic and abiotic stress, secondary metabolism, glutathione transferase, cytochrome P450, PHOR1 regulation of transcription, ribosome biogenesis and signaling receptor kinases leucine rich repeat XI and DUF. Meanwhile, gene expressions in the HLB susceptible citrus trees were increased in RNA, DNA and protein biosynthesis, cell wall, cell organization, amino acid metabolism synthesis, beta 1,3 glycan hydrolases, signaling receptor kinases leucine rich repeat III and major intrinsic transport proteins. Then, we used MapMan (Urbanczyk-Wochniak et al., 2006) software to display and analyze the functional classes that were significantly different in HLB tolerant and HLB susceptible citrus trees. The MapMan provides an overview of pathways and functions identified by PageMan. As shown in Figure 4, cell wall, lipids, minor CHO, Tetrapyrrole and secondary metabolism are the major categories in which genes are differentially expressed between HLB tolerant and HLB susceptible citrus trees. We used the JGI citrus genome annotations for the MapMan analysis.

### Analyzing DE genes using protein-protein interaction network

**Figure 5:**
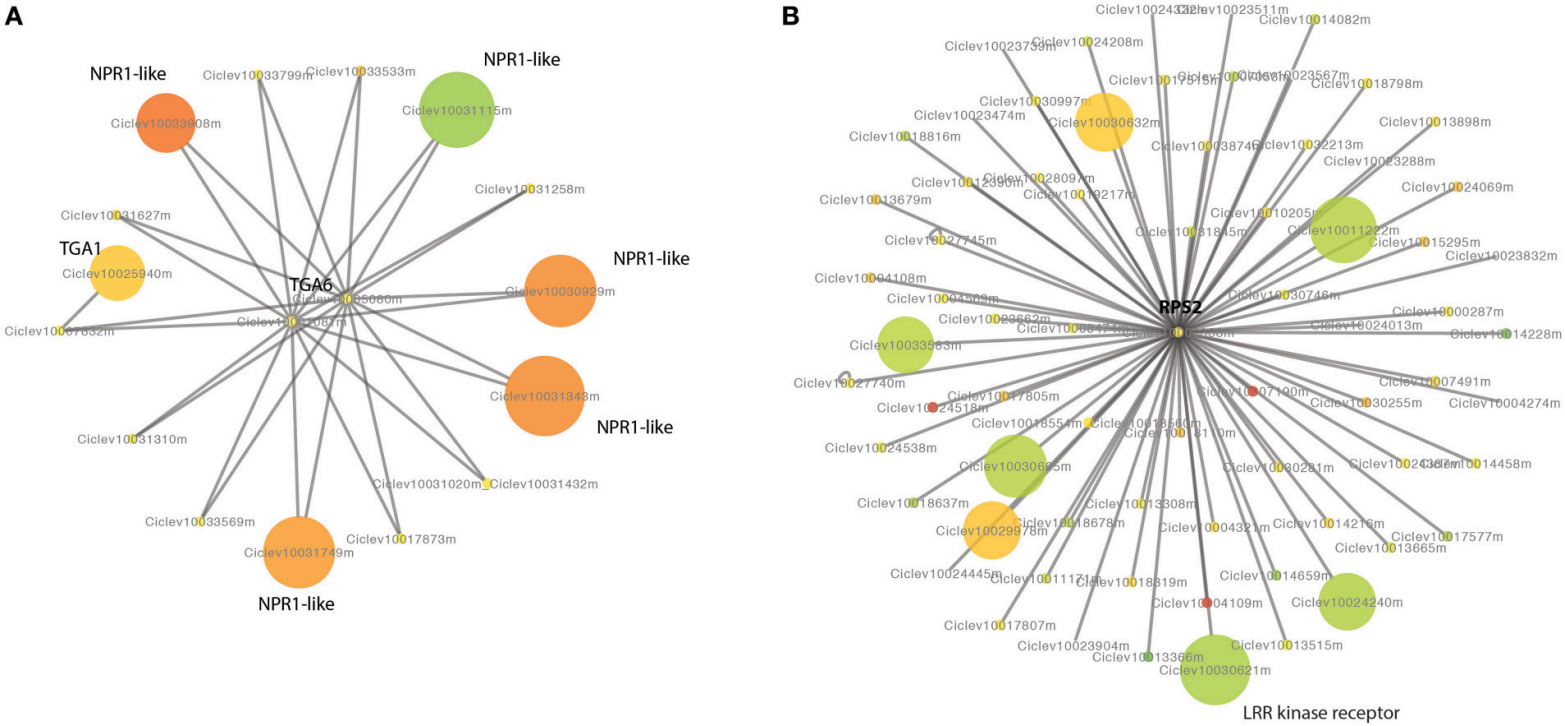
**Protein-protein interaction network of citrus predicted based on the knowledge of IPP network of *Arabidopsis***. **(A)** Interactions between NPR1-like proteins and TGA transcription factors. **(B)** Proteins interacting with RPS2. The DE genes are indicated in colors, red for up-expressed and green for down-expressed in HLB tolerant “Jackson” trees compared to HLB susceptible “Marsh” trees. The size of the nodes negatively correlates to the FDR and the color correlates to the log2 fold ratio.

We predicted a PPI network of citrus using the PPIs of Arabidopsis. There are 1,259 proteins and 2,298 interactions in our Citrus PPI network. Among 1,259 proteins, 42 proteins were differentially expressed between the HLB tolerant and susceptible citrus. An interesting PPI sub-network included 14 citrus NPR1-like proteins and three TGA proteins. NPR1-like genes were reported to be required for plant disease resistance in Arabidopsis (Despres et al., 2003; Liu et al., 2005). NPR1-like proteins interact with members of the TGA class of transcription factors and regulate their DNA binding activity. There were four NPR1-like genes significantly up-expressed in HLB tolerant citrus trees and one NPR1-like gene up-expressed in HLB susceptible citrus trees (Figure 5A). There is also one TGA gene up-expressed in HLB tolerant citrus trees. Another interesting sub-network includes the RPS2 protein, which interacts with 66 LRR kinase receptors. The Arabidopsis RPS2 protein is a specific resistance gene for the avirulence gene avrRpt2 of *Pseudomonas syringae* strains (Kunkel et al., 1993). RPS2 interacts with other receptors, such as LRR kinase receptors. There were two LRR kinase receptors significantly up-expressed in HLB tolerant citrus trees and four LRR kinase receptors significantly up-expressed in susceptible citrus trees (Figure 5B). Other interested interactions are between two lipoxygenase genes, LOX2 (Ciclev10017776m and Ciclev10014207m) and EIF4E, a translation initiation factor (Freire et al., 2000). The plant oxylipins have been reported to function as signals in defense and development. Disruption of lox3 increased the resistance to fungal pathogens in maize (Gao et al., 2007). The two LOX2 genes were down-expressed in the HLB tolerant citrus trees.

### Analyzing DE genes related to disease response

**Figure 6:**
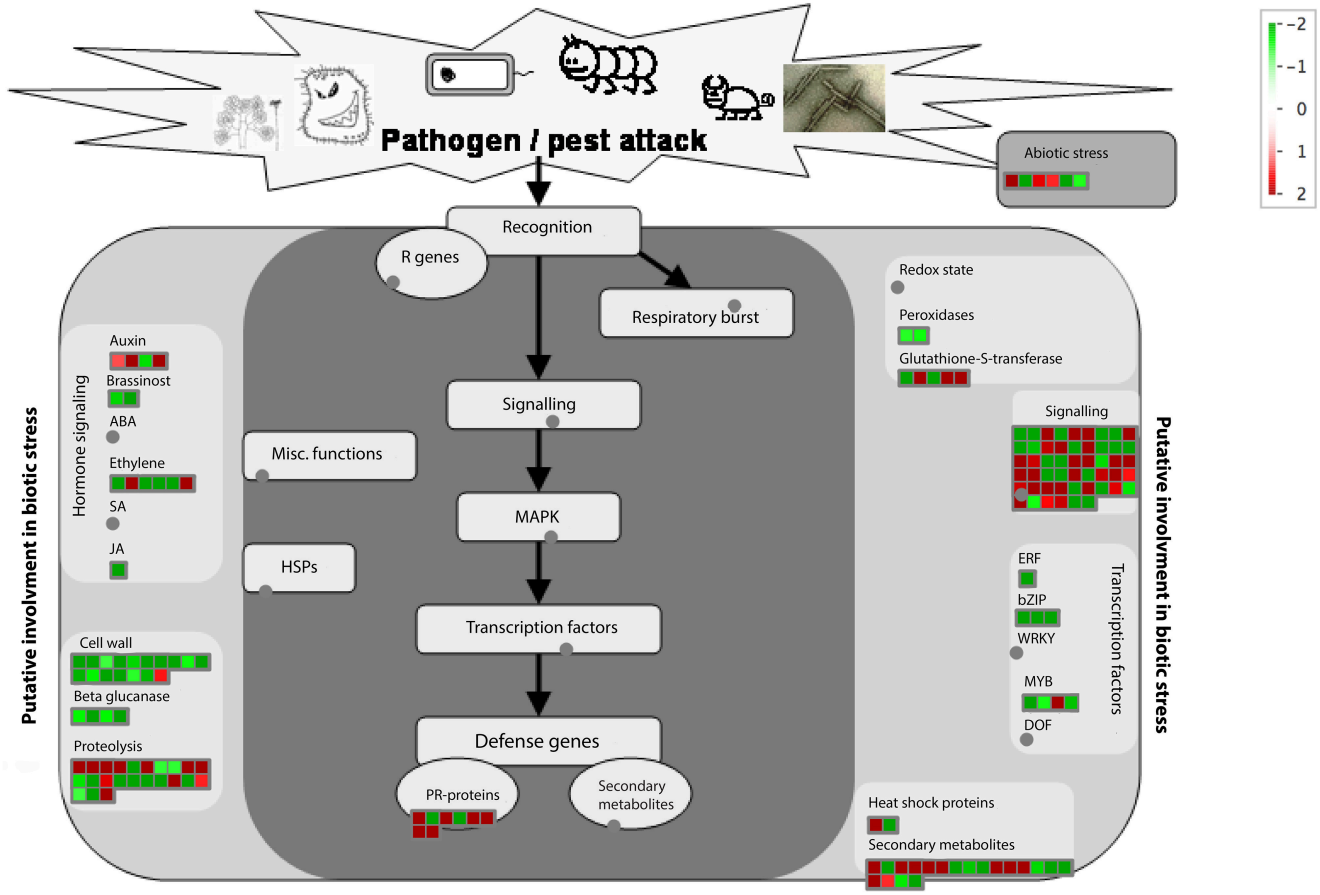
**Visualization of functional categories of 155 disease response genes differentially expressed between HLB tolerant “Jackson” and HLB susceptible “Marsh” trees**. Figure created by MapMan (Urbanczyk-Wochniak et al., 2006).

We then applied the MapMan software to identify and visualize genes related to disease response. The MapMan annotated 155 genes (Table S7) related to disease response and Figure 6 is the overview of functional categories of these genes. The 155 genes belong to the following functional categories: hormone signaling, cell wall, beta glucanase, proteolysis, glutathione-S-transferase, signaling, transcription factors, abiotic stress, Pathogenesis Related (PR)-genes and secondary metabolites. Understanding the expression differences of those 155 genes may help elucidate the response difference between HLB tolerant and HLB susceptible citrus trees after the disease is well-established.

#### Differences in secondary metabolites

Secondary metabolites play essential roles in plant defense. In our study, there were 19 genes involving secondary metabolites synthesis among the 155 DE genes between the HLB-susceptible and tolerant cultivars (Table S7). The beta-amyrin synthase (Ciclev10033766, Ciclev10033766, Ciclev10033930, Ciclev10033377), cycloartenol synthase (Ciclev10010416) and Camelliol C synthase (Ciclev10031967), which are involved in terpenoid biosynthesis, were significantly up-expressed in the HLB tolerant citrus trees while the terpene synthase related genes (CiClev10014707, Ciclev10017785) were down-expressed in the HLB tolerant citrus trees. Plant O-methyltransferases (OMTs) are involved in the synthesis of a variety of secondary metabolites including phenylpropanoids, flavonoids and alkaloids, which are important in disease resistance (Lam et al., 2007). We identified an OMT gene (Ciclev10015724m) that was up-expressed in the HLB tolerant cultivar. Flavonoids are widely distributed in plants and serve as physiological regulators. Some flavonoids can play inhibitory roles against plant pathogens, e.g., *Fusarium oxysporum* (Galeotti et al., 2008). Two enzymes (ciclev10019871m, ciclev10003684m) involved in flavonoid biosynthesis were up-expressed in HLB tolerant “Jackson” while two isoflavone reductases (ciclev10013926m, ciclev10012523m) were down-expressed. There were three HXXXD-type acyl-transferases (Ciclev10028364m, Ciclev10011647m and Ciclev10031464m) that were up-expressed in HLB susceptible “Marsh.” HXXXD-type acyl-transferase, which belongs to the BAHD acyl-transferase superfamily, plays an important role in the biosynthesis of salicylic acid (SA), which can trigger systemic acquired resistance (SAR) in plants to provide resistance to biotrophic and hemi-biotrophic pathogens (Zheng et al., 2009).

#### Differences in pathogenesis related genes

There were six TIR-NBS-LRR genes and two Kunitz trypsin inhibitor (KTI) genes among the 155 DE genes between the HLB-susceptible and tolerant cultivars (Table S7). The pathogenesis-related (PR) genes were significantly up-expressed in the HLB tolerant citrus trees. Four TIR-NBS-LRR genes were up-expressed and all two KTI genes were also up-expressed in HLB tolerant citrus trees.

#### Differences in cell wall genes

All 17 cell wall related genes among the 155 disease response DE genes were up-expressed in the HLB susceptible citrus trees, such as cellulose synthase or transferase (ciclev10030861m, ciclev10014531m, ciclev10023570m), cellulases (ciclev10019799m, ciclev10014994m, ciclev10028301m), expansins (ciclev10012518m, ciclev10012611m, ciclev10013804m), and pectinesterases (ciclev10007806m, ciclev10013004m, ciclev10007993m). The cellulases, expansins and pectinesterases are all related to cell wall breakdown. The high expressions of these genes may have contributed to the development of HLB symptoms in susceptible citrus trees.

#### Differences in abiotic stress related genes

In the 155 DE genes, there were two heat shock genes (Hsp70) differentially expressed between HLB tolerant and HLB susceptible cultivars. Hsp70 is involved in resistance development in response to drought, high salt and heat stresses in plants (Lee and Schoffl, 1996). Hsp70 has also been reported to be related to plant disease resistance. For example, Hsp70 is the major target of the virulence effector gene, HopI1, of pathogenic *Pseudomonas syringae* (Jelenska et al., 2010). Hsp70 gene Ciclev10033341m was up-expressed in HLB tolerant citrus trees, while Hsp70 gene Ciclev10015130m was up-expressed in HLB susceptible citrus trees.

HLB-affected trees often display water-stress related symptoms. There were three genes related to dehydration response among the 155 DE genes. Two of them (ciclev10030080m and ciclev10000463m) were up-expressed in HLB tolerant “Jackson” and an ERD (early-responsive to dehydration stress) family gene (Ciclev10024532m) was up-expressed in HLB susceptible “Marsh.”

#### Differences in receptor like kinases

The majority of the 51 signaling related genes among the 155 DE genes were receptor-like kinases (RLKs) (Table S7), which are transmembrane receptor genes similar to animal receptor tyrosine kinases. The RLK genes play important roles in plant disease resistance (Shiu and Bleecker, 2001). Overall, there were more RLK genes that had been up-expressed in HLB tolerant “Jackson.” Out of 822 RLK genes in the citrus genome, 243 and 152 RLK genes were up-expressed (log2 ratio > 1) in the HLB tolerant and HLB susceptible citrus trees, respectively. There were 42 RLK genes among the 155 disease response DE genes. These 42 RLK genes were enriched mainly in two classes of RLKs, LRR-RLKs and DUF26-RLKs. Among 42 RLK genes, 29 are LRR-RLKs and 12 are DUF26-RLKs, respectively.

#### Differences in transcription factors

There were 39 transcription factors in identified DE genes (Table S8). The majority (32 genes) of 39 transcription factors were up-expressed in HLB susceptible “Marsh.” There were 8 transcription factors in 155 DE genes and 7 of them were up-expressed in HLB susceptible “Marsh.” The ethylene-responsive element-binding (EREB) transcription factors are often involved in ethylene responses. We identified an ERF (Ethylene response factor) gene, Ciclev10005701m, which was down-expressed in the HLB tolerant “Jackson” trees (Zhao et al., 2012). The basic region/leucine zipper motif (bZIP) transcription factors in plants can regulate processes related to pathogen defense (Jakoby et al., 2002). There were 3 bZIP transcription factors (ciclev10031441m, ciclev10022056m, ciclev10015966m) in 155 DE genes and all were up-expressed in HLB susceptible “Marsh.” MYB transcription factors (TF) play important roles in disease resistance, abiotic stress tolerance and other biological processes (Ambawat et al., 2013). We identified 4 MYB transcription factors in the DE genes. Only one of them was up-expressed in HLB tolerant “Jackson” and 3 of them were up-expressed in HLB susceptible “Marsh.”

#### Differences in proteolysis related genes

Ubiquitin mediated protein degradation plays an important role in plant-pathogen interactions (Zeng et al., 2006). There were 23 protein degradation related genes among the 155 DE genes (Table S7). Among these 23 genes, 11 were up-expressed in HLB tolerant “Jackson” and 12 were up-expressed in HLB susceptible “Marsh.” Of these 23 genes, 14 were ubiquitin-related genes and 12 of them were E3 ubiquitin ligase genes. 4 of 12 E3 genes were up-expressed in HLB tolerant “Jackson” and 8 of them were up-expressed in HLB susceptible “Marsh.” Among these E3 genes, 4 belong to RING sub-family and 8 belong to the F-box sub-family. There was one E2 ubiquitin-conjugating enzyme gene (Ciclev10016889m), which was significantly up-expressed in the HLB susceptible “Marsh.”

#### Differences in hormone signaling pathways

There were 12 hormone signaling genes in 155 DE genes. Among them, 2, 4, and 6 genes related to brassinosteroids, auxins and ethylene, respectively. Both brassinosteroid biosynthesis related genes (ciclev10013348m, ciclev10006027m) were down-expressed in HLB tolerant “Jackson.” Brassinosteroids can suppress the salicylate-mediated immunity in rice (De Vleesschauwer et al., 2012). Down expression of brassinosteroids may contribute to suppression of HLB in tolerant citrus trees.

The DMR6 and DMR6-like genes may be involved in the biosynthesis of ethylene. Recently, it is reported that DRM6-like genes were able to suppress immunity in Arabidopsis (Zeilmaker et al., 2015). A mutation of DMR6 had led to downy mildew resistance in Arabidopsis (van Damme et al., 2008). There were 4 DMR6-like genes in our list and all of them were down-expressed in HLB tolerant “Jackson” trees.

Auxin is a key hormone in pathogenesis and plant defense (Vidhyasekaran, 2015). Auxin was found to promote the expression of expansins in tomato (Catala et al., 2000), rice (Ding et al., 2008), and soybean (Downes et al., 2001) contributing to breakage of plant cell walls, which are natural barriers against pathogens (Fu and Wang, 2011). On the other hand, the suppression of expansin genes may promote resistance to pathogens. Small auxin-up RNA (SAUR) genes have been reported to negatively regulate auxin synthesis in rice (Kant et al., 2009). There were three SAUR-like genes in the 155 DE genes and all of these genes were up-expressed in HLB tolerant “Jackson,” which may also contribute to the down expression of expansin genes in HLB tolerant citrus trees.

#### Differences in beta glucanase

Beta glucanase plays multiple roles in plants, including being involved in response to biotic and abiotic stresses. Studies have shown that beta-1,3-glucanase deficiency reduced susceptibility to a viral disease in plants (Beffa et al., 1996). There were four beta-1,3-glucanase genes in the 155 DE genes and all were down-expressed in HLB tolerant “Jackson.” This implies that beta-1,3-glucanase may negatively regulate HLB as it does in the viral disease.

### Validating DE genes using real time PCR

We conducted real-time PCR (RT-PCR) analyses in the HLB tolerant and susceptible citrus trees to validate the expression patterns of a subset of differentially expressed genes identified by RNA-Seq. We selected 14 up-expressed genes and 19 down-expressed genes based on their differential expression level and their predicted functions in disease development (Table S9). We validated the gene expression level using the mature infected leaves of tolerant citrus (R20T18 and R20T17) and susceptible citrus (R19T24 and R20T24). Out of the 14 up-expressed genes, 11 genes were validated to be significantly up-expressed or only expressed in one of the two cultivars (Table S9). All of the 19 down-expressed genes were validated to be down-expressed or not expressed in the tolerant citrus.

To further confirm the RNA-Seq results, we also selected sister trees with similar disease index: (R20T09 and R20T10) from the tolerant citrus “Jackson” and (R20T07 and R20T08) from the susceptible citrus “Marsh” to validate the expression patterns of these DE genes (Table S9). Out of the 14 up-expressed genes from RNA-Seq, 13 genes were significantly up-expressed in the tolerant citrus. Out of the 19 down-expressed genes, 15 genes were down-expressed in the tolerant citrus. The results showed that the gene expressions of most genes in agreement with the RNA-Seq results had similar expression pattern after HLB infection, which further supported the tolerant citrus and the susceptible citrus may have different defense response to Las infection.

## Discussion

Both the HLB tolerant and HLB susceptible citrus trees were naturally infected with Las in the field and had been infected with high titers of Las for at least 4 years when this study was initiated. The HLB tolerant “Jackson” trees showed typical blotchy mottle symptoms during the growth season, but maintained good canopies and had normal fruits while the HLB susceptible “Marsh” trees displayed typical HLB symptoms along with heavily decline and misshapen fruits. In this study, we used RNA-Seq to profile the transcriptome of three HLB tolerant “Jackson” trees and three closely related HLB susceptible “Marsh” trees. We have identified 686 genes that differentially expressed between these HLB tolerant and HLB susceptible citrus trees.

**Figure 7:**
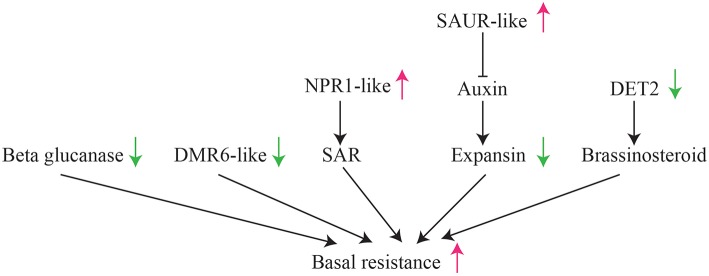
**Illustration of suppression/activation of multiple pathways in HLB tolerant “Jackson” citrus trees compared to HLB susceptible “Marsh” trees**.

Specially, we found multiple pathways suppressed or activated in HLB tolerant citrus trees, which lead to the activation of basal resistance in HLB tolerant citrus trees (Figure 7). For example, down expression of beta glucanases, DMR6-like genes, expansin and DET2 may be associated with the suppression of citrus immunity, while activation of NPR1-like genes may induce the resistance responses to HLB. One of these findings is supported by over expression of an Arabidopsis NPR1 in citrus enhances resistance against HLB (Dutt et al., 2015). Although the mechanism triggering the suppression and activation of these genes is still unclear, further experimental studies on the NBS-LRR and RLK genes differentially expressed between HLB tolerant and HLB susceptible citrus trees may provide understanding of HLB related receptor genes.

Meanwhile, some of our DE genes that are related to the downstream of HLB infection are consistent with previous studies. For example, the lipoxygenase isozyme, LOX2, which is required for wound-induced jasmonic acid accumulation (Bell et al., 1995), has showed that it was up-regulated in the leaves after HLB infection in previous studies (Albrecht and Bowman, 2008; Kim et al., 2009; Fan et al., 2011). There were two LOX2 genes, Ciclev10014207 and Ciclev10017776, in our DE gene list. Both of them were significantly up expressed in the susceptible citrus leaves. Another example is the PP2 gene, which is related to callose deposition to plug the sieve pores in HLB infected citrus. Several previous studies showed that PP2 were significantly up-regulated in the leaves and roots after HLB infection (Tatineni et al., 2008; Kim et al., 2009; Mafra et al., 2013). The blockage of the phloem vessels affected the translocation of the important nutrients from the source (mature leaves) to the sink (young leaves). In our DE gene list, one phloem gene, PP2-A1 (Ciclev10033500), was also significantly up expressed in the susceptible citrus.

## Conclusion

It is evident that there are significant differences of HLB resistance/tolerance among citrus cultivars. In this study, we were able to advance our knowledge on citrus genomics and transcriptomics related to HLB by updating the citrus genome via annotation, and analyzing DE genes between closely related HLB tolerant and susceptible cultivars. This study provided new insights into HLB-tolerance by revealing the differences in secondary metabolites, pathogenesis related genes, transcription factors, hormone signaling pathways and receptor-like kinases, etc. between HLB tolerant and susceptible plants. Moreover, we also identified some potential targets, such as DMR6-like and NPR1-like genes for breeding HLB-tolerant citrus in the future.

## Author contributions

Conceived and designed the experiment: ES, FL, YD. Performed the experiment LZ, XY. Analyzed the data: YW, FL. Wrote the manuscript: ES, FL, YD.

### Conflict of interest statement

The authors declare that the research was conducted in the absence of any commercial or financial relationships that could be construed as a potential conflict of interest.

## Supplementary material

Figure S1 **Overview of gene location mapped by RNA-Seq reads**.

Figure S2 **Boxplot of gene read counts of six samples. (A)** Raw data without normalization. **(B)** Normalization by DESeq.

Table S1 **New gene models predicted from RNA-Seq data using PASA pipeline**.

Table S2 **Differentially expressed genes identified by DESeq**.

Table S3 **Differential alternative splicing events identified by rMAST**.

Table S4 **Genes with significantly differential isoform usage between HLB tolerant and susceptible citrus trees identified by IUTA**.

Table S5 **GO enrichment analysis of the DE genes**.

Table S6 **Gene functional classification of DE genes using PageMan**.

Table S7 **DE genes related to disease response identified by MapMan**.

Table S8 **Differentially expressed transcription factors**.

Table S9 **DE genes validated using real time PCR**.
