# Supplementary figures and images for: Transcriptome Profiling of Huanglongbing (HLB) Tolerant and Susceptible Citrus Plants Reveals the Role of Basal Resistance in HLB Tolerance
Source: Front Plant Sci. 2016 Jun 28;7:933. doi: 10.3389/fpls.2016.00933 (PMC4923198; doi:10.3389/fpls.2016.00933)

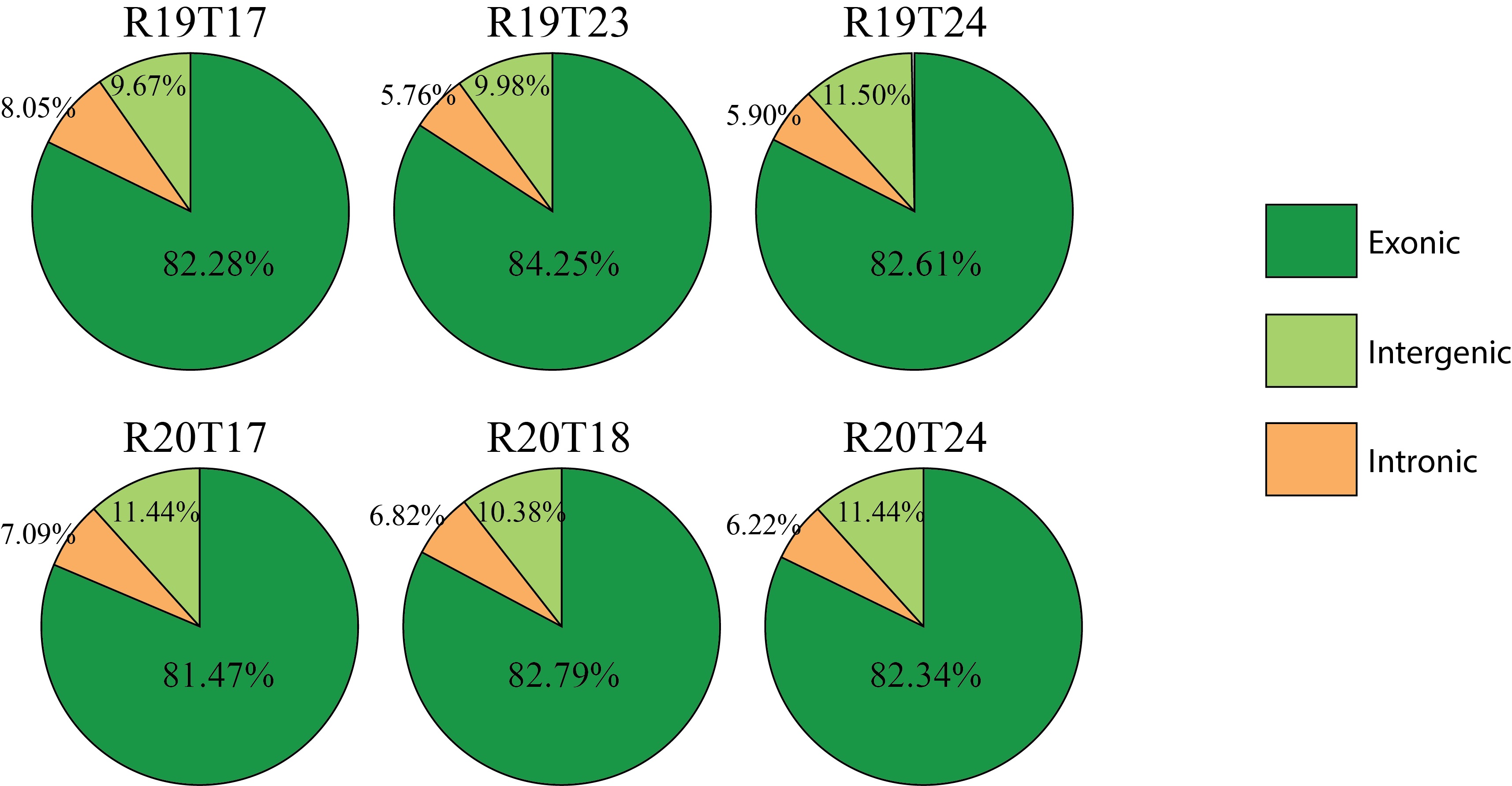

Supplement: Figure S1 — Overview of gene location mapped by RNA-Seq reads. [file Image1.jpg]

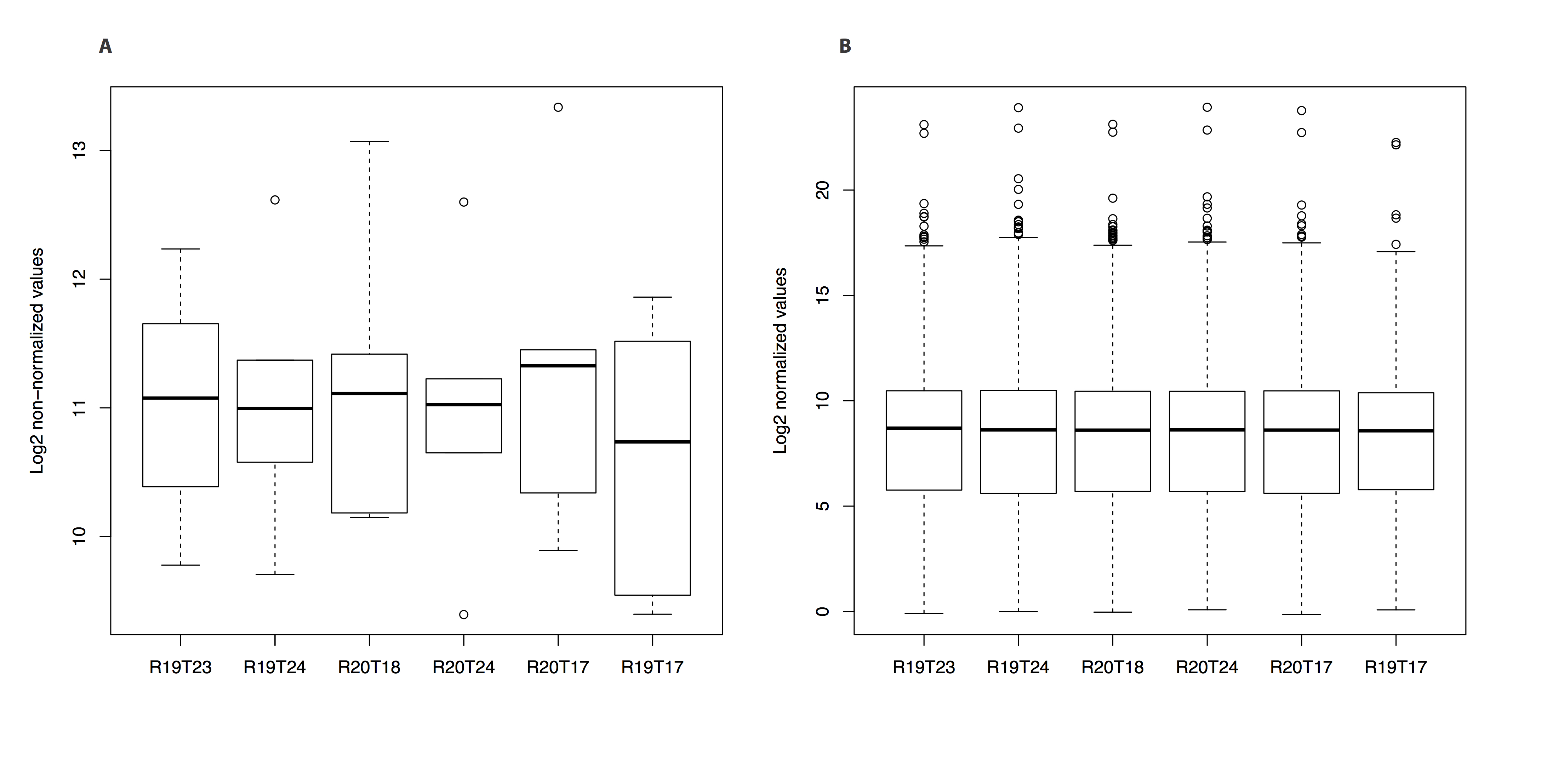

Supplement: Figure S2 — Boxplot of gene read counts of six samples. (A) Raw data without normalization. (B) Normalization by DESeq. [file Image2.jpg]

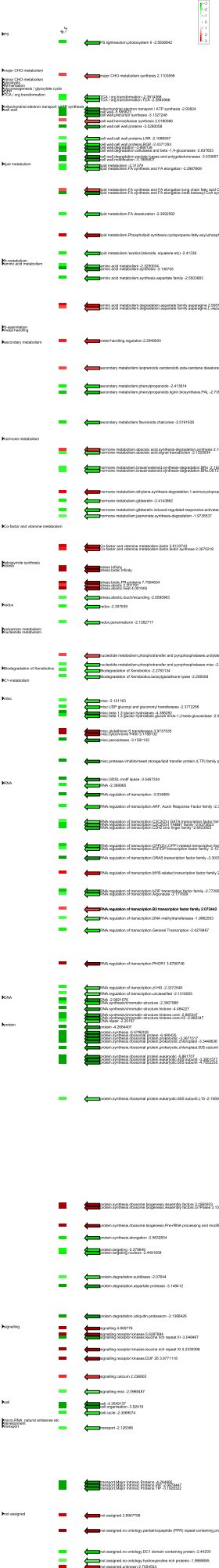

Supplement: Table S6 — Gene functional classification of DE genes using PageMan. [file Table6.PDF]
