# Supplementary material for: Transcriptome Profiling of Huanglongbing (HLB) Tolerant and Susceptible Citrus Plants Reveals the Role of Basal Resistance in HLB Tolerance
Source: Front Plant Sci. 2016 Jun 28;7:933. doi: 10.3389/fpls.2016.00933 (PMC4923198; doi:10.3389/fpls.2016.00933)
Supplement: Table S2 — Differentially expressed genes identified by DESeq. [file Table2.PDF]

Figure S2. Differentially expressed genes identified by DESeq

R19T23, R19T24 and R20T24 were HLB susceptible samples, R20T18, R20T17 and R19T17 were HLB tolerance samples. The DE analysis was carried out by comparing the tolerance samples with susceptible sampels.

ID	R19T23	R19T24	R20T24	R20T18	R20T17	R19T17	baseMeanA	baseMeanB	2FoldChange	pval	padj	FuncAnn
Ciclev10013495m.g	799	1129	1199	2	1	0	1043.35062	0.95375387	-10.095319	1.16E-36	2.61E-32	
Ciclev10020491m.g	1	2	3	1314	1380	746	2.03144725	1115.74252	9.10128052	1.47E-35	1.26E-31	DegP protease 1;
Ciclev10003018m.g	859	1200	1149	1	0	4	1067.50072	1.7712075	-9.2352881	1.68E-35	1.26E-31	
g.4398	1	0	4	1113	1214	691	1.72453831	980.127248	9.15061508	9.60E-35	5.40E-31	
Ciclev10023040m.g	0	1	3	981	941	595	1.3941662	819.648603	9.19945917	2.09E-33	9.39E-30	
Ciclev10023913m.g	0	0	1	474	466	662	0.35448907	534.868151	10.5592265	2.55E-30	9.55E-27	disease resistance protein (TIR-NBS-LRR class), putative;
Ciclev10026680m.g	1	3	9	978	1144	692	4.48908065	915.351627	7.67176223	4.01E-30	1.29E-26	Na+/H+ antiporter 6;
Ciclev10010049m.g	327	456	512	0	1	0	432.549477	0.30350209	-10.476943	1.68E-28	4.73E-25	ubiquitin-conjugating enzyme 22;
Ciclev10033981m.g	0	0	0	310	435	284	0	335.484228	Inf	6.15E-27	1.54E-23	
Ciclev10031775m.g	0	2	5	1047	590	916	2.43384334	850.625728	8.44914438	3.34E-26	7.52E-23	KAR-UP F-box 1;
Ciclev10033500m.g	451	346	305	1	0	1	360.809523	0.68664629	-9.0374546	1.31E-25	2.68E-22	phloem protein 2-A1;
Ciclev10026868m.g	1	9	11	1077	1197	1644	7.18225281	1307.79212	7.50848109	3.86E-25	7.25E-22	
Ciclev10023524m.g	0	0	1	306	295	362	0.35448907	319.892024	9.81762818	4.69E-25	8.12E-22	TTF-type zinc finger protein with HAT dimerisation domain;
Ciclev10029707m.g	2	0	1	376	400	319	0.96765316	358.973178	8.53517031	9.94E-25	1.60E-21	
Ciclev10011675m.g	238	285	354	0	1	0	292.704872	0.30350209	-9.9135246	4.93E-24	7.39E-21	F-box/RNI-like superfamily protein;
Ciclev10011873m.g	28	22	40	2081	1846	1174	30.039238	1661.27678	5.78930043	6.63E-24	9.32E-21	alpha/beta-Hydrolases superfamily protein;
Ciclev10003409m.g	15	7	10	590	878	936	10.4585144	796.682204	6.25125451	1.53E-23	2.02E-20	Protein kinase family protein with leucine-rich repeat domain;
Ciclev10014207m.g	1123	1070	911	0	6	44	1021.07911	17.7279102	-5.8479284	2.82E-23	3.53E-20	lipoxygenase 2;
g.26810	1	0	0	234	236	336	0.30658205	269.176806	9.77806517	4.58E-23	5.43E-20	
Ciclev10020066m.g	3	3	0	350	454	262	1.91184315	346.302355	7.50092422	1.54E-22	1.73E-19	Major facilitator superfamily protein;
Ciclev10017776m.g	4894	5061	6262	9	96	327	5393.89072	150.279505	-5.1656061	4.44E-22	4.76E-19	lipoxygenase 2;
Ciclev10021888m.g	2	0	4	433	502	217	2.03112035	371.587486	7.51528238	7.78E-22	7.95E-19	O-methyltransferase 1;
Ciclev10029708m.g	2	7	13	487	587	361	7.53641497	467.000899	5.95340311	2.68E-20	2.62E-17	
g.58299	1	0	2	983	572	1135	1.01556018	903.527602	9.79714914	4.69E-20	4.40E-17	
Ciclev10015993m.g	1265	1419	1061	63	33	19	1233.20107	37.3673877	-5.0444845	3.18E-19	2.86E-16	O-methyltransferase family protein;
Ciclev10033953m.g	14	12	9	593	743	332	11.4509383	538.326477	5.55494366	3.68E-19	3.18E-16	TTF-type zinc finger protein with HAT dimerisation domain;
Ciclev10023778m.g	0	0	0	112	173	198	0	160.501	Inf	1.30E-18	1.08E-15	Leucine-rich repeat transmembrane protein kinase protein;
Ciclev10013569m.g	197	150	186	0	0	1	175.93648	0.3615204	-8.9267619	1.61E-18	1.29E-15	pfkB-like carbohydrate kinase family protein;
Ciclev10013216m.g	148	310	261	0	0	0	240.41248	0	#NAME?	2.57E-18	1.99E-15	
g.9442	255	264	336	3	3	6	284.591285	4.05500635	-6.1330435	7.69E-18	5.77E-15	
Ciclev10015130m.g	156	158	171	0	0	1	160.694872	0.3615204	-8.7960311	1.47E-17	1.07E-14	mitochondrial HSO70 2;
Ciclev10033409m.g	164	197	281	1	1	1	215.038587	0.99014838	-7.7627351	1.98E-17	1.40E-14	
Ciclev10001024m.g	443	405	450	7	13	12	429.269023	10.5596532	-5.3452477	2.25E-17	1.52E-14	cytochrome P450, family 716, subfamily A, polypeptide 1;
Ciclev10020105m.g	4	10	12	328	415	420	8.78718701	384.433227	5.45118783	2.29E-17	1.52E-14	Cysteine proteinases superfamily protein;
Ciclev10023716m.g	0	1	2	156	260	136	1.03967714	178.796956	7.42604279	3.23E-17	2.07E-14	3-beta hydroxysteroid dehydrogenase/isomerase family protein;
Ciclev10015724m.g	14	11	12	1645	1513	672	12.1837065	1236.97246	6.66571648	6.68E-17	4.17E-14	O-methyltransferase family protein;
g.15290	177	98	134	0	0	0	134.175059	0	#NAME?	7.25E-17	4.41E-14	Transposon Ty3-I Gag-Pol polyprotein
Ciclev10030087m.g	2197	1572	1489	93	117	35	1721.25381	78.3996661	-4.4564685	1.22E-16	7.23E-14	cytochrome P450, family 87, subfamily A, polypeptide 6;
g.46082	1	1	4	217	160	178	2.05523731	183.463284	6.48004257	9.61E-16	5.54E-13	

ID	R19T23	R19T24	R20T24	R20T18	R20T17	R19T17	baseMeanA	baseMeanB	2FoldChange	pval	padj	FuncAnn
Ciclev10013437m.g	125	136	140	0	0	1	132.92629	0.3615204	-8.5223337	1.17E-15	6.60E-13	F-box/RNI-like/FBD-like domains-containing protein;
Ciclev10027009m.g	169	125	68	0	0	0	117.254998	0	#NAME?	1.32E-15	7.25E-13	
Ciclev10024175m.g	0	0	1	133	127	133	0.35448907	129.868722	8.51709717	1.99E-15	1.07E-12	HSP20-like chaperones superfamily protein;
g.5948	0	0	0	123	222	88	0	139.181743	Inf	4.50E-15	2.35E-12	
Ciclev10013892m.g	148	109	119	0	1	0	123.604533	0.30350209	-8.6698095	5.40E-15	2.76E-12	FAR1-related sequence 5;
Ciclev10003728m.g	70	124	125	0	0	0	106.778553	0	#NAME?	1.41E-14	7.05E-12	ADP-ribosylation factor C1;
Ciclev10011889m.g	1	0	8	1944	1534	3269	3.14249457	2279.42713	9.50254532	1.57E-14	7.68E-12	UDP-glucosyl transferase 73C2;
Ciclev10013355m.g	175	116	94	1	0	1	125.334915	0.68664629	-7.5120055	2.19E-14	1.05E-11	NB-ARC domain-containing disease resistance protein;
Ciclev10031343m.g	35	24	31	618	637	529	29.6563088	585.502923	4.30326543	3.80E-14	1.78E-11	NPR1-like protein 3;
Ciclev10031514m.g	0	0	3	114	115	182	1.0634672	137.763805	7.01727755	5.41E-14	2.48E-11	Cysteine proteinases superfamily protein;
Ciclev10024580m.g	8	5	13	292	328	198	8.71450924	266.066484	4.93222356	5.89E-14	2.65E-11	Histone superfamily protein;
Ciclev10006839m.g	115	126	146	3	0	0	128.680413	0.97537768	-7.0436158	6.96E-14	3.07E-11	glutathione S-transferase tau 7;
Ciclev10011972m.g	122	251	247	2	2	0	207.967259	1.25725596	-7.3699342	1.08E-13	4.69E-11	alpha/beta-Hydrolases superfamily protein;
Ciclev10007181m.g	391	261	281	3	9	24	305.797448	12.3833861	-4.6260986	1.24E-13	5.27E-11	Eukaryotic aspartyl protease family protein;
g.61000	119	93	65	0	0	0	90.2800602	0	#NAME?	3.49E-13	1.45E-10	
Ciclev10023985m.g	99	75	100	0	0	0	90.6029545	0	#NAME?	3.71E-13	1.52E-10	disease resistance protein (TIR-NBS-LRR class), putative;
Ciclev10027067m.g	211	256	279	2	7	18	248.250206	9.28213363	-4.7411946	4.75E-13	1.91E-10	receptor-like protein kinase 1;
Ciclev10006105m.g	130	69	69	0	0	0	87.1336429	0	#NAME?	6.90E-13	2.72E-10	MLP-like protein 423;
Ciclev10031115m.g	268	288	314	12	10	19	288.714868	13.8054192	-4.3863388	2.14E-12	8.30E-10	NPR1-like protein 3;
Ciclev10010969m.g	119	59	72	0	0	0	81.5177175	0	#NAME?	2.59E-12	9.86E-10	Leucine-rich repeat protein kinase family protein;
Ciclev10003666m.g	6	0	2	131	154	134	2.54847041	137.774547	5.75653397	2.74E-12	1.03E-09	
Ciclev10023335m.g	6	7	4	196	417	205	5.57234157	264.396727	5.56827681	3.38E-12	1.25E-09	
Ciclev10014567m.g	324	600	555	29	21	14	494.493417	20.8634803	-4.5668996	4.60E-12	1.66E-09	Protein kinase superfamily protein;
g.69950	0	0	0	236	296	532	0	358.895182	Inf	4.64E-12	1.66E-09	Putative AC transposase
Ciclev10006027m.g	171	144	90	1	1	7	131.950202	3.15927079	-5.3842582	1.18E-11	4.12E-09	C-8,7 sterol isomerase;
Ciclev10023520m.g	3	10	22	305	215	213	12.0254956	241.420191	4.32737808	1.19E-11	4.12E-09	cytochrome P450, family 72, subfamily A, polypeptide 10;
Ciclev10021050m.g	3	4	5	194	497	456	4.01498748	378.768263	6.55977615	1.33E-11	4.53E-09	sulfotransferase 2A;
Ciclev10012877m.g	94	89	70	0	1	0	83.0651583	0.30350209	-8.0963932	1.76E-11	5.90E-09	
Ciclev10013856m.g	161	212	256	4	17	7	210.217099	8.99068184	-4.5473057	2.04E-11	6.74E-09	Protein with RING/U-box and TRAF-like domains;
Ciclev10023516m.g	135	227	236	5	5	16	200.11667	8.92746633	-4.4864468	2.55E-11	8.31E-09	
Ciclev10012059m.g	2	8	2	295	136	188	3.96773426	205.154258	5.69224988	2.92E-11	9.39E-09	chromatin protein family;
Ciclev10016019m.g	3908	2943	3215	134	355	536	3311.05215	345.085046	-3.2622659	3.02E-11	9.56E-09	strictosidine synthase-like 4;
g.47673	1	2	0	165	247	78	0.96798006	156.809379	7.33981881	3.44E-11	1.07E-08	
Ciclev10012953m.g	241337	179152	260089	6179	58602	9299	225433.686	23156.5603	-3.2832102	4.16E-11	1.28E-08	lipid transfer protein 1;
Ciclev10030929m.g	12	10	12	239	247	183	11.2398434	218.828337	4.28310573	5.36E-11	1.61E-08	NPR1-like protein 3;
Ciclev10010498m.g	347	229	206	13	25	7	255.13879	14.3448316	-4.1526793	5.38E-11	1.61E-08	ArfGap/RecO-like zinc finger domain-containing protein;
g.55050	17	7	7	524	559	204	10.0082113	413.773796	5.36958623	5.66E-11	1.68E-08	Uncharacterized mitochondrial protein AtMg00310
g.59737	85	69	56	0	0	0	68.7290929	0	#NAME?	6.15E-11	1.80E-08	Pentatricopeptide repeat-containing protein At2g20540
Ciclev10031749m.g	45	22	51	580	567	329	39.1505125	479.598913	3.61472529	6.77E-11	1.95E-08	NPR1-like protein 3;
Ciclev10028415m.g	172	251	177	5	11	13	198.482127	9.66391763	-4.3602571	7.48E-11	2.13E-08	Jojoba acyl CoA reductase-related male sterility protein;
Ciclev10027478m.g	198	99	110	2	4	6	132.436244	4.03338254	-5.0371639	7.82E-11	2.20E-08	Thioredoxin superfamily protein;
Ciclev10030291m.g	816	977	1088	98	98	93	958.94798	95.2269393	-3.3320109	1.53E-10	4.25E-08	decapping 1;
Ciclev10013454m.g	6460	3255	5785	156	584	599	5107.66452	444.515578	-3.5223579	1.57E-10	4.25E-08	GAST1 protein homolog 4;

ID	R19T23	R19T24	R20T24	R20T18	R20T17	R19T17	baseMeanA	baseMeanB	2FoldChange	pval	padj	FuncAnn
Ciclev10033341m.g	78	26	28	656	569	309	42.4372676	497.685077	3.55182945	1.57E-10	4.25E-08	heat shock cognate protein 70-1;
Ciclev10013779m.g	2	6	7	128	122	168	5.07878158	139.378796	4.77838486	2.12E-10	5.68E-08	
g.54606	90	58	46	0	0	0	63.0794234	0	#NAME?	2.58E-10	6.82E-08	
Ciclev10012611m.g	17431	12654	7877	838	815	2072	12321.0072	1268.87997	-3.2794927	2.93E-10	7.66E-08	expansin A5;
Ciclev10013211m.g	1	0	0	68	83	137	0.30658205	96.8275289	8.30300025	3.38E-10	8.74E-08	UBX domain-containing protein;
Ciclev10014632m.g	7915	10837	12335	898	1637	1310	10383.0046	1262.38769	-3.039997	4.54E-10	1.16E-07	terpene synthase 14;
Ciclev10023127m.g	3	8	5	110	153	147	5.3377835	135.343166	4.66423744	5.34E-10	1.35E-07	gamma-glutamyl hydrolase 1;
Ciclev10012523m.g	64	95	68	1	0	1	75.1429128	0.68664629	-6.7739261	5.58E-10	1.40E-07	NmrA-like negative transcriptional regulator family protein;
Ciclev10029804m.g	54	79	50	0	0	0	60.4051051	0	#NAME?	5.91E-10	1.46E-07	
Ciclev10011743m.g	57	37	30	933	559	1064	40.3457118	857.657832	4.40991494	7.25E-10	1.77E-07	Plant protein of unknown function (DUF247);
Ciclev10025880m.g	1680	1369	774	110	136	200	1242.15931	149.344212	-3.056137	8.73E-10	2.11E-07	Protein of unknown function, DUF642;
Ciclev10030621m.g	590	342	391	66	32	27	432.587691	40.9314265	-3.4017118	9.27E-10	2.22E-07	Leucine-rich repeat receptor-like protein kinase family protein;
Ciclev10030536m.g	15	13	21	206	185	293	16.3420881	229.049298	3.8089939	1.41E-09	3.33E-07	Leucine-rich repeat protein kinase family protein;
Ciclev10029498m.g	2259	1936	1533	189	242	298	1876.23385	242.629378	-2.9510135	1.88E-09	4.40E-07	histone H2A 12;
Ciclev10024495m.g	30	15	34	260	318	333	26.2105747	301.432689	3.52361499	2.08E-09	4.83E-07	Radical SAM superfamily protein;
g.30947	54	76	79	0	2	0	69.693191	0.60700417	-6.8431675	2.13E-09	4.90E-07	Putative ribonuclease H protein At1g65750
g.51141	114	39	56	0	0	0	67.6990021	0	#NAME?	2.82E-09	6.36E-07	LINE-1 reverse transcriptase homolog
g.63413	33	54	77	0	0	0	55.2706118	0	#NAME?	2.83E-09	6.36E-07	
Ciclev10021063m.g	103	96	109	4	6	0	101.964363	3.12151608	-5.0296743	3.16E-09	7.05E-07	Leucine-rich repeat protein kinase family protein;
Ciclev10033090m.g	0	0	0	113	161	38	0	99.3408369	Inf	3.73E-09	8.20E-07	NPR1-like protein 4;
Ciclev10002795m.g	1469	1410	1409	137	257	164	1416.12972	181.831629	-2.9612783	3.76E-09	8.20E-07	histone H2A 7;
Ciclev10024450m.g	4	6	1	79	134	102	3.56501128	103.229306	4.85580215	4.34E-09	9.39E-07	triosephosphate isomerase;
Ciclev10013619m.g	83	35	44	0	0	0	52.6182939	0	#NAME?	4.73E-09	1.01E-06	Argonaute family protein;
g.4298	0	1	0	86	66	31	0.330699	59.1990969	7.48391265	5.52E-09	1.17E-06	
Ciclev10001578m.g	518	381	344	3300	2219	3283	406.750059	2933.25805	2.85028954	6.98E-09	1.47E-06	SGNH hydrolase-type esterase superfamily protein;
Ciclev10022596m.g	81	112	56	5	1	0	81.7228219	1.92913155	-5.4047156	7.63E-09	1.59E-06	
Ciclev10020999m.g	8	9	7	158	179	88	7.91037087	137.51056	4.11965326	8.03E-09	1.66E-06	RNA-binding (RRM/RBD/RNP motifs) family protein;
Ciclev10033373m.g	39	59	55	0	0	0	50.9648397	0	#NAME?	9.01E-09	1.84E-06	UDP-Glycosyltransferase superfamily protein;
Ciclev10018835m.g	40	13	10	223	196	297	20.1072596	239.361042	3.57339999	9.55E-09	1.94E-06	Disease resistance protein (TIR-NBS-LRR class) family;
Ciclev10012833m.g	52	78	44	0	1	0	57.3343076	0.30350209	-7.5615484	1.01E-08	2.02E-06	Adenine nucleotide alpha hydrolases-like superfamily protein;
Ciclev10021714m.g	226	113	85	1137	881	1128	136.788101	1044.84849	2.93327912	1.10E-08	2.19E-06	Nodulin MtN3 family protein;
Ciclev10004063m.g	29	58	63	0	0	0	50.4042327	0	#NAME?	1.12E-08	2.21E-06	
Ciclev10023012m.g	2013	951	1024	43	75	164	1294.64122	96.0324158	-3.7528871	1.21E-08	2.37E-06	Gibberellin-regulated family protein;
Ciclev10006937m.g	489	280	291	5	104	5	345.67066	34.9974484	-3.3040765	1.28E-08	2.48E-06	
Ciclev10023264m.g	1658	1436	868	159	197	201	1290.89331	184.150529	-2.8094123	1.33E-08	2.56E-06	gamma histone variant H2AX;
Ciclev10029878m.g	4	1	2	54	83	111	2.26600532	82.876236	5.19273533	1.55E-08	2.95E-06	4-hydroxy-3-methylbut-2-enyl diphosphate reductase;
Ciclev10023448m.g	1	4	1	76	114	49	1.98386713	77.0233053	5.27890773	1.77E-08	3.34E-06	proteinaceous RNase P 3;
Ciclev10016785m.g	2329	2950	1834	274	453	353	2339.7246	354.187641	-2.7237529	2.26E-08	4.24E-06	
Ciclev10013741m.g	492	480	577	47	99	51	514.11408	63.7651639	-3.0112481	2.40E-08	4.47E-06	Protein of unknown function (DUF594);
Ciclev10003406m.g	187	153	78	8	1	18	135.577937	9.41187647	-3.8484962	2.51E-08	4.63E-06	cytochrome P450, family 87, subfamily A, polypeptide 6;
Ciclev10012553m.g	125569	109124	62161	20464	11877	15962	96619.794	16028.6592	-2.591665	3.09E-08	5.65E-06	gamma tonoplast intrinsic protein;
Ciclev10011222m.g	1388	837	880	232	173	47	1014.28132	144.926527	-2.8070643	3.18E-08	5.77E-06	Leucine-rich repeat receptor-like protein kinase family protein;
Ciclev10023777m.g	45	19	19	2287	2021	618	26.8147654	1580.36024	5.88108201	3.31E-08	5.95E-06	

ID	R19T23	R19T24	R20T24	R20T18	R20T17	R19T17	baseMeanA	baseMeanB	2FoldChange	pval	padj	FuncAnn
Ciclev10003586m.g	1	0	1	49	62	64	0.66107111	57.8856038	6.45225531	3.81E-08	6.80E-06	
Ciclev10019327m.g	57	53	64	1	1	0	57.6895241	0.62862798	-6.5199591	3.97E-08	7.03E-06	Protein of unknown function (DUF3133);
Ciclev10001446m.g	132	213	219	14	17	19	188.540823	16.5801856	-3.5073449	4.17E-08	7.34E-06	Zinc-binding alcohol dehydrogenase family protein;
Ciclev10018487m.g	0	0	1	198	176	445	0.35448907	278.667873	9.61858984	5.06E-08	8.82E-06	pleiotropic drug resistance 12;
Ciclev10013015m.g	49	45	60	0	1	0	51.1733194	0.30350209	-7.3975416	5.74E-08	9.93E-06	Cytidine/deoxycytidylate deaminase family protein;
g.54694	189	277	91	3	8	0	181.806136	3.40339436	-5.7392828	5.84E-08	1.00E-05	
Ciclev10030369m.g	33	69	51	0	1	0	51.0143812	0.30350209	-7.3930538	6.13E-08	1.04E-05	zinc finger (C2H2 type) family protein;
Ciclev10032702m.g	81	80	145	973	629	519	102.689981	694.879394	2.75846717	6.19E-08	1.05E-05	glutathione S-transferase tau 7;
Ciclev10013518m.g	162	77	66	3	5	2	98.5263932	3.21592891	-4.9372028	6.46E-08	1.09E-05	NB-ARC domain-containing disease resistance protein;
Ciclev10013535m.g	146	211	284	20	16	11	215.213363	15.3352756	-3.8108417	7.53E-08	1.25E-05	heteroglycan glucosidase 1;
Ciclev10017196m.g	464	395	371	33	58	72	404.39562	54.3617444	-2.8951037	8.57E-08	1.42E-05	
Ciclev10011671m.g	184	36	126	0	0	0	112.981883	0	#NAME?	8.62E-08	1.42E-05	Plant protein of unknown function (DUF247);
Ciclev10033679m.g	157	212	164	12	17	20	176.377777	16.2914542	-3.4364815	8.81E-08	1.44E-05	nuclear factor Y, subunit C13;
Ciclev10010717m.g	0	2	2	38	70	79	1.37037614	62.1600417	5.50334363	1.12E-07	1.82E-05	ribosomal protein L11 methyltransferase-related;
g.61948	72	31	27	0	0	0	41.8967812	0	#NAME?	1.17E-07	1.88E-05	
g.43469	0	1	0	30	46	69	0.330699	48.6597805	7.2010673	1.26E-07	2.02E-05	
Ciclev10017142m.g	276	130	338	11	12	3	247.424819	8.30297105	-4.8972187	1.44E-07	2.29E-05	
Ciclev10012029m.g	24	23	11	169	323	142	18.8634259	204.313347	3.43711983	1.86E-07	2.93E-05	F-box family protein;
Ciclev10011747m.g	807	313	191	1	0	1	418.627911	0.68664629	-9.2518857	1.89E-07	2.95E-05	Plant protein of unknown function (DUF247);
Ciclev10019254m.g	12	12	13	132	139	159	12.2557305	142.585152	3.54029537	2.08E-07	3.23E-05	exocyst subunit exo70 family protein E2;
Ciclev10003082m.g	7	17	24	150	146	201	16.275695	165.745789	3.34818117	2.17E-07	3.34E-05	
Ciclev10012633m.g	7655	5067	4279	941	514	1652	5539.39613	1059.17524	-2.3867874	2.35E-07	3.60E-05	plasma membrane intrinsic protein 2;4;
Ciclev10020843m.g	153	144	140	901	1034	706	144.156179	861.99299	2.58004346	2.60E-07	3.96E-05	senescence-related gene 1;
Ciclev10029006m.g	4801	3522	1626	538	39	39	3213.02152	200.853607	-3.9997144	2.66E-07	4.02E-05	unknown seed protein like 1;
Ciclev10033466m.g	0	3	5	41	98	76	2.76454234	70.5489166	4.67351135	2.92E-07	4.39E-05	Leucine-rich repeat protein kinase family protein;
Ciclev10017131m.g	1298	1249	1005	186	194	236	1167.24806	204.671636	-2.5117281	2.99E-07	4.45E-05	Histone superfamily protein;
g.25134	0	0	1	42	58	38	0.35448907	44.9961837	6.98791771	3.06E-07	4.53E-05	Putative ribonuclease H protein At1g65750
g.26569	5	6	5	79	141	61	5.28954959	90.5314841	4.09720282	3.08E-07	4.53E-05	
Ciclev10013580m.g	5	2	1	89	64	60	2.54879731	70.0515621	4.78052867	3.36E-07	4.91E-05	sugar transporter 1;
Ciclev10013298m.g	59	94	82	2	3	6	78.2421505	3.72988046	-4.3907447	3.61E-07	5.24E-05	
Ciclev10010929m.g	189	68	78	2	3	0	108.081686	1.56075804	-6.1137314	3.64E-07	5.24E-05	pleiotropic drug resistance 9;
g.54612	53	25	39	0	0	0	38.3413971	0	#NAME?	4.03E-07	5.77E-05	Uncharacterized mitochondrial protein AtMg00810
Ciclev10010386m.g	78	25	57	3	0	0	52.3867515	0.97537768	-5.7470972	4.31E-07	6.12E-05	multidrug resistance-associated protein 3;
Ciclev10017058m.g	52	140	223	0	0	0	141.291189	0	#NAME?	4.33E-07	6.12E-05	
Ciclev10028699m.g	273	36	243	0	0	0	181.742906	0	#NAME?	4.45E-07	6.26E-05	2-oxoglutarate (2OG) and Fe(II)-dependent oxygenase superfamily protein;
Ciclev10024532m.g	274	191	271	42	38	15	243.233527	30.6111728	-2.9902119	4.68E-07	6.54E-05	ERD (early-responsive to dehydration stress) family protein;
Ciclev10012865m.g	27613	24259	29642	1169	4120	10672	26995.8421	5488.64649	-2.2982149	5.24E-07	7.27E-05	
g.45580	109	52	54	2	1	6	69.7562008	3.12287628	-4.4813741	5.35E-07	7.36E-05	Retrovirus-related Pol polyprotein from transposon TNT 1-94
Ciclev10019799m.g	169	69	116	6	3	20	115.751329	10.0916697	-3.519792	5.37E-07	7.36E-05	cellulase 2;
Ciclev10013895m.g	356	389	265	22	50	76	331.724724	49.8034245	-2.7356697	5.44E-07	7.42E-05	Protein of unknown function (DUF1191);
Ciclev10025377m.g	298	152	216	11	23	50	218.197337	28.6329529	-2.9298851	5.82E-07	7.89E-05	Cytochrome P450 superfamily protein;
Ciclev10028896m.g	8	6	10	2798	1785	501	7.98174105	1632.57519	7.67623025	5.99E-07	8.07E-05	NmrA-like negative transcriptional regulator family protein;

ID	R19T23	R19T24	R20T24	R20T18	R20T17	R19T17	baseMeanA	baseMeanB	2FoldChange	pval	padj	FuncAnn
Ciclev10020723m.g	41996	27426	23089	4506	6838	7113	30129.7686	6111.85916	-2.3015064	6.83E-07	9.14E-05	reversibly glycosylated polypeptide 3;
Ciclev10031263m.g	144	128	75	4	6	19	113.063967	9.99040373	-3.5004524	7.32E-07	9.74E-05	RNI-like superfamily protein;
g.44620	591	332	453	140	85	0	451.565606	71.3153022	-2.662652	7.96E-07	0.00010538	Benzoate carboxyl methyltransferase
Ciclev10003084m.g	11	28	21	2005	2232	425	20.076245	1482.94024	6.20682719	8.22E-07	0.00010819	
Ciclev10007033m.g	219	107	133	3	12	34	149.673307	16.9090964	-3.1459455	8.68E-07	0.00011347	Major facilitator superfamily protein;
Ciclev10027680m.g	168	141	136	10	10	27	146.344856	16.0473306	-3.1889668	9.19E-07	0.00011952	pleiotropic drug resistance 3;
Ciclev10019618m.g	403	292	313	12	45	98	331.071751	52.988104	-2.6434035	9.37E-07	0.00012112	protein kinases;ubiquitin-protein ligases;
g.61199	798	802	791	225	174	35	790.273925	138.615903	-2.51126	9.42E-07	0.00012114	
Ciclev10009291m.g	24226	20351	17139	2604	7989	2047	20232.9002	4011.33825	-2.3345476	1.02E-06	0.00012982	Pathogenesis-related thaumatin superfamily protein;
Ciclev10030665m.g	259	256	330	51	49	24	281.045087	40.1295124	-2.8080661	1.02E-06	0.00012982	Leucine-rich repeat receptor-like protein kinase family protein;
Ciclev10028465m.g	10	2	15	198	115	83	9.04455445	129.28386	3.83734893	1.06E-06	0.00013437	UDP-glucosyl transferase 73C2;
Ciclev10026742m.g	1897	1838	1276	111	252	622	1641.73896	337.43719	-2.2825339	1.18E-06	0.00014849	Histone superfamily protein;
Ciclev10013699m.g	154	125	81	14	19	0	117.264625	10.3183021	-3.5064904	1.19E-06	0.00014862	Protein of unknown function, DUF617;
g.77207	24	41	40	0	0	0	35.0961909	0	#NAME?	1.30E-06	0.00016101	
g.77332	350	425	508	61	51	105	427.931238	73.2709281	-2.5460662	1.36E-06	0.00016753	
g.46830	2841	1973	2091	68	276	1051	2264.70537	485.833079	-2.2207907	1.39E-06	0.00016933	Putative elongation of fatty acids protein DDB_G0272012
Ciclev10019826m.g_Cic	1285	264	219	3704	2644	2385	558.895572	2868.95198	2.35987317	1.39E-06	0.00016933	cytochrome P450, family 76, subfamily C, polypeptide 4;cytochrome P450, family 76, subfamily C, polypeptide 4;
Ciclev10018519m.g	6967	5365	5255	917	855	1869	5772.99732	1233.31636	-2.2267776	1.39E-06	0.00016933	RNA helicase family protein;
Ciclev10022854m.g	723	1209	1163	4886	4978	5181	1033.7447	4972.4357	2.26607278	1.44E-06	0.0001736	
Ciclev10013864m.g	131	242	233	35	37	9	202.787359	25.862667	-2.9710248	1.52E-06	0.00018301	MATE efflux family protein;
Ciclev10003604m.g	42	45	46	0	2	0	44.0643982	0.60700417	-6.1817633	1.56E-06	0.00018606	
Ciclev10013438m.g	14	13	7	125	133	101	11.0726592	117.520075	3.40783358	1.56E-06	0.00018606	MATE efflux family protein;
Ciclev10017068m.g	788	435	504	34	192	87	564.103208	100.778956	-2.4847647	1.66E-06	0.00019653	
Ciclev10006569m.g	287	389	322	43	38	81	330.776439	54.7966452	-2.593697	1.74E-06	0.00020468	2-oxoglutarate (2OG) and Fe(II)-dependent oxygenase superfamily protein;
Ciclev10026181m.g	2156	2628	1984	279	697	414	2233.37418	451.920524	-2.305084	1.76E-06	0.00020672	Pathogenesis-related thaumatin superfamily protein;
Ciclev10002881m.g	2243	1262	1451	97	219	689	1619.36931	347.091726	-2.2220432	1.94E-06	0.00022596	Glycosyl hydrolase family 35 protein;
Ciclev10019731m.g	95	108	109	593	669	444	103.480095	556.357608	2.42665921	2.04E-06	0.000236	cytochrome P450, family 72, subfamily A, polypeptide 15;
Ciclev10025188m.g	9	20	15	176	150	286	14.6905545	206.142305	3.81067985	2.23E-06	0.00025754	Leucine-rich repeat protein kinase family protein;
Ciclev10003479m.g	55	44	51	2	2	0	49.4917111	1.25725596	-5.2988366	2.30E-06	0.00026441	Zinc finger C-x8-C-x5-C-x3-H type family protein;
Ciclev10004959m.g	2722	1606	1665	92	352	806	1955.84323	428.129761	-2.1916707	2.33E-06	0.00026627	NAD(P)-binding Rossmann-fold superfamily protein;
Ciclev10033983m.g	397	295	283	57	79	25	319.589684	51.5468507	-2.6322646	2.35E-06	0.00026732	
Ciclev10033469m.g	1904	1945	1468	308	326	470	1747.33173	368.995044	-2.2434802	2.37E-06	0.00026763	Zinc-binding alcohol dehydrogenase family protein;
Ciclev10030101m.g	26	48	25	0	0	0	32.7069121	0	#NAME?	2.75E-06	0.00030738	Aluminium activated malate transporter family protein;
Ciclev10018392m.g	59	30	27	0	1	0	37.5805156	0.30350209	-6.9521346	2.75E-06	0.00030738	
Ciclev10019274m.g	464	314	283	50	55	77	346.413962	60.7859803	-2.5106866	2.77E-06	0.00030738	RNI-like superfamily protein;
Ciclev10020189m.g	35	67	65	1582	1254	462	55.9289942	1061.9632	4.24699358	2.77E-06	0.00030738	Class I glutamine amidotransferase-like superfamily protein;
Ciclev10002813m.g	6493	3310	3795	1143	1360	245	4430.53694	872.95423	-2.3435036	2.83E-06	0.00031233	arabinogalactan protein 10;
Ciclev10028364m.g	2680	1241	936	4	72	149	1563.83911	77.0191937	-4.3437303	2.92E-06	0.00032032	HXXXD-type acyl-transferase family protein;
Ciclev10010949m.g	180	50	57	0	0	0	91.9255953	0	#NAME?	3.00E-06	0.00032767	NB-ARC domain-containing disease resistance protein;
Ciclev10013609m.g	2194	2239	3360	83	836	496	2604.15934	460.027312	-2.5010263	3.24E-06	0.00035185	

ID	R19T23	R19T24	R20T24	R20T18	R20T17	R19T17	baseMeanA	baseMeanB	2FoldChange	pval	padj	FuncAnn
Ciclev10001812m.g	9957	5248	7188	214	2300	2454	7336.21321	1654.80281	-2.1483763	3.47E-06	0.00037513	N-terminal nucleophile aminohydrolases (Ntn hydrolases) superfamily protein;
Ciclev10021802m.g	3821	2790	2838	425	763	878	3100.14019	687.165509	-2.1736039	3.54E-06	0.00038063	RING/U-box superfamily protein;
Ciclev10013524m.g	0	6	2	40	95	45	2.69317216	58.1061519	4.43131256	3.58E-06	0.00038203	Leucine-rich repeat protein kinase family protein;
Ciclev10024591m.g	16	13	24	667	639	170	17.7121374	472.255271	4.73675668	3.58E-06	0.00038203	MAP kinase kinase 7;
Ciclev10014994m.g	3729	1861	2002	585	572	319	2468.36241	479.126848	-2.3650747	3.72E-06	0.00039462	glycosyl hydrolase 9B1;
Ciclev10031960m.g	159	126	108	808	809	382	128.699439	646.335702	2.32827792	3.75E-06	0.00039624	Pyridoxal phosphate (PLP)-dependent transferases superfamily protein;
Ciclev10013661m.g	2832	3775	1525	612	282	5	2657.22492	286.372236	-3.2139568	3.84E-06	0.00040353	
Ciclev10022628m.g	626	918	860	193	107	190	800.362643	163.912897	-2.2877245	4.00E-06	0.0004184	
Ciclev10024312m.g	161	100	129	5	17	23	128.158699	15.1001342	-3.0852981	4.04E-06	0.00042112	
Ciclev10011317m.g	10803	11437	10872	3034	2909	1528	10948.2155	2421.7227	-2.1765902	4.15E-06	0.00043011	Thiamine pyrophosphate dependent pyruvate decarboxylase family protein;
Ciclev10030752m.g	42	49	32	0	2	0	40.4243473	0.60700417	-6.0573742	4.23E-06	0.00043628	Leucine-rich repeat protein kinase family protein;
Ciclev10013302m.g	0	0	0	32	28	70	0	44.2085151	Inf	4.38E-06	0.00045006	
Ciclev10015019m.g	84	49	58	349	385	336	62.5175089	351.788095	2.49237446	4.47E-06	0.00045698	UDP-Glycosyltransferase superfamily protein;
Ciclev10017219m.g	11264	17071	8126	213	465	3827	11979.281	1593.91886	-2.9098912	4.49E-06	0.0004574	cell wall protein precursor, putative;
Ciclev10029826m.g	54	29	39	1	1	0	39.9707752	0.62862798	-5.9905953	4.73E-06	0.00047812	phosphate transporter 1;1;
Ciclev10022725m.g	3751	3110	5394	762	1010	493	4090.57718	732.512595	-2.481379	4.74E-06	0.00047812	2-oxoglutarate (2OG) and Fe(II)-dependent oxygenase superfamily protein;
Ciclev10027531m.g	292	254	321	34	69	46	287.310495	48.6258628	-2.5628149	5.02E-06	0.00050279	RWP-RK domain-containing protein;
Ciclev10017011m.g	149	227	263	46	40	13	213.980023	31.7956397	-2.7505753	5.03E-06	0.00050279	Saccharopine dehydrogenase ;
Ciclev10011476m.g	36	45	27	206	201	275	35.4896136	227.397964	2.67975056	5.18E-06	0.00051534	Jojoba acyl CoA reductase-related male sterility protein;
Ciclev10031967m.g_Cic	462	216	269	1604	1933	830	308.429449	1408.2334	2.19087404	5.29E-06	0.00052412	camelliol C synthase 1;Terpenoid cyclases family protein;lupeol synthase 2;lupeol synthase 2;
Ciclev10016664m.g	1111	755	861	265	159	154	895.505487	190.089335	-2.2360247	5.64E-06	0.00055671	
Ciclev10017097m.g	1234	725	1123	149	366	146	1016.17024	212.3075	-2.2589149	5.72E-06	0.00056241	proline-rich family protein;
Ciclev10018578m.g	2	1	1	74	62	153	1.29835216	98.1890669	6.24080874	6.60E-06	0.00064543	Disease resistance protein (TIR-NBS-LRR class) family;
Ciclev10004342m.g	236	214	217	29	30	49	220.047077	36.2482132	-2.6018304	6.69E-06	0.00065138	acyl-CoA dehydrogenase-related;
Ciclev10025820m.g	653	266	311	13	32	63	398.410111	36.7144887	-3.4398328	6.72E-06	0.00065138	PQ-loop repeat family protein / transmembrane family protein;
Ciclev10000018m.g	213	231	195	916	1117	1580	210.818814	1208.02938	2.51858002	7.06E-06	0.00068155	callose synthase 5;
Ciclev10033783m.g	82	113	131	511	476	626	108.946783	536.918096	2.30107843	7.21E-06	0.00069317	
Ciclev10009541m.g	3352	1531	1836	156	410	530	2184.80512	366.761308	-2.5745912	7.63E-06	0.00073001	Plant invertase/pectin methylesterase inhibitor superfamily protein;
Ciclev10027526m.g	61	91	87	7	5	8	79.6356629	6.68555489	-3.5742954	8.49E-06	0.00080945	FAD-binding Berberine family protein;
Ciclev10016684m.g	71251	55305	46531	5072	23342	13034	56628.3165	13445.4411	-2.0744066	8.55E-06	0.0008112	Plant invertase/pectin methylesterase inhibitor superfamily protein;
Ciclev10032663m.g	1150	1155	959	195	401	133	1074.48172	233.186099	-2.2040872	9.00E-06	0.00084687	MATE efflux family protein;
g.60722	66	106	98	6	4	16	90.028438	8.94909013	-3.3305679	9.02E-06	0.00084687	Putative AC transposase
Ciclev10012776m.g	1010	566	554	165	168	124	693.210446	149.462653	-2.2135084	9.03E-06	0.00084687	calmodulin like 42;
Ciclev10019871m.g	155	148	105	642	628	660	133.685022	637.933598	2.25456843	9.25E-06	0.00086309	2-oxoglutarate (2OG) and Fe(II)-dependent oxygenase superfamily protein;
Ciclev10006737m.g	194	591	646	14	31	7	483.919965	16.49097	-4.8750203	1.05E-05	0.00097619	Protein of unknown function (DUF594);

ID	R19T23	R19T24	R20T24	R20T18	R20T17	R19T17	baseMeanA	baseMeanB	2FoldChange	pval	padj	FuncAnn
Ciclev10020332m.g	1637	438	741	6	4	60	909.397371	24.8559878	-5.1932455	1.06E-05	0.00097638	GDSL-like Lipase/Acylhydrolase superfamily protein;
Ciclev10011628m.g	2698	1776	1968	348	661	503	2112.11427	495.603452	-2.0914298	1.06E-05	0.00097638	
Ciclev10022230m.g	30926	24909	23220	7552	8950	2720	25949.974	6155.0299	-2.0758953	1.07E-05	0.00098406	plasmodesmata callose-binding protein 3;
Ciclev10013306m.g	13	11	20	152	118	89	14.713037	117.407698	2.99636003	1.08E-05	0.00098406	G10 family protein;
Ciclev10002388m.g	184	184	161	6	27	49	174.332453	27.8598114	-2.6455838	1.09E-05	0.00099431	TRAM, LAG1 and CLN8 (TLC) lipid-sensing domain containing protein;
Ciclev10001683m.g	204	119	114	29	20	12	142.307672	19.8369374	-2.8427522	1.10E-05	0.00099431	beta HLH protein 93;
Ciclev10011533m.g	2543	3271	2775	656	843	544	2845.06174	665.801943	-2.095295	1.11E-05	0.00100561	cytochrome P450, family 94, subfamily D, polypeptide 2;
Ciclev10004641m.g	44	7	44	499	278	208	31.4020219	321.807644	3.35726923	1.15E-05	0.00103038	acyl-activating enzyme 7;
Ciclev10033507m.g	46	78	57	11	0	0	60.1031732	3.57638482	-4.0708673	1.15E-05	0.00103038	Protein kinase family protein with leucine-rich repeat domain;
Ciclev10002409m.g	125	191	264	31	25	9	195.071379	20.9201384	-3.2210378	1.16E-05	0.00103149	TRAF-like family protein;
Ciclev10033908m.g	1	3	1	90	78	21	1.65316813	60.5264215	5.1942597	1.18E-05	0.00105105	NPR1-like protein 4;
g.14469	33	42	12	0	0	0	28.2604345	0	#NAME?	1.21E-05	0.0010697	
Ciclev10013804m.g	515	216	331	1	6	121	346.65662	45.8901071	-2.9172522	1.25E-05	0.00110636	expansin A8;
Ciclev10029360m.g	90	194	118	871	590	419	133.577701	613.727931	2.19992006	1.26E-05	0.00110858	Calcium-binding EF-hand family protein;
Ciclev10010265m.g	409	177	147	3	2	34	236.035673	13.8740755	-4.0885414	1.31E-05	0.00115103	NB-ARC domain-containing disease resistance protein;
Ciclev10023475m.g	8	1	5	53	70	74	4.5558007	65.2293281	3.83974423	1.40E-05	0.0012166	N-terminal nucleophile aminohydrolases (Ntn hydrolases) superfamily protein;
Ciclev10018876m.g	3	4	4	47	83	50	3.66049842	58.5476102	3.99949828	1.41E-05	0.0012166	Ubiquitin carboxyl-terminal hydrolase-related protein;
Ciclev10014825m.g	903	574	400	35	137	196	608.460443	123.817191	-2.2969518	1.41E-05	0.0012166	SKU5 similar 4;
Ciclev10015614m.g	26273	22640	16819	5318	7170	3848	21504.0072	5296.25996	-2.0215597	1.44E-05	0.00124378	fatty acid desaturase 2;
Ciclev10023238m.g	1	0	0	62	66	171	0.30658205	102.008932	8.37820655	1.46E-05	0.00125138	double-stranded DNA binding;
Ciclev10022056m.g	127	104	80	12	21	3	101.687742	11.3596157	-3.1621598	1.51E-05	0.00129172	
Ciclev10033338m.g	28	33	57	0	3	0	39.7032412	0.91050626	-5.446444	1.52E-05	0.00129172	Bifunctional inhibitor/lipid-transfer protein/seed storage 2S albumin superfamily protein;
Ciclev10008705m.g	35	78	68	265	426	244	60.6301504	303.661228	2.32435541	1.54E-05	0.00130628	lysine-ketoglutarate reductase/saccharopine dehydrogenase bifunctional enzyme;
Ciclev10000073m.g	234	203	261	1032	1148	833	231.393743	985.096811	2.08991566	1.64E-05	0.0013872	NB-ARC domain-containing disease resistance protein;
Ciclev10033720m.g	57	37	48	1	2	3	46.7265149	2.01669127	-4.5341793	1.73E-05	0.00145687	Protein of unknown function (DUF674);
Ciclev10013599m.g	30	40	27	0	1	0	31.9966263	0.30350209	-6.7200696	1.76E-05	0.00148159	Ribosomal protein S4;
Ciclev10024083m.g	1	2	4	173	74	279	2.38593632	179.570126	6.23384801	1.84E-05	0.0015379	receptor like protein 15;
Ciclev10021176m.g	768	221	817	16	43	12	598.157058	22.5908488	-4.726714	1.85E-05	0.0015379	Tetratricopeptide repeat (TPR)-like superfamily protein;
Ciclev10032088m.g	2231	1806	817	136	195	409	1570.84451	251.261873	-2.6442767	1.94E-05	0.00160688	Ferritin/ribonucleotide reductase-like family protein;
Ciclev10004050m.g	280	157	158	11	49	43	193.771989	33.9933643	-2.511035	1.97E-05	0.00162639	homeobox protein 33;
g.77208	24	33	38	0	0	1	31.7416207	0.3615204	-6.4561549	2.01E-05	0.00165449	Uncharacterized mitochondrial protein AtMg00310
Ciclev10027807m.g	1252	1269	933	87	230	511	1134.23606	282.828358	-2.0037222	2.03E-05	0.00167067	Minichromosome maintenance (MCM2/3/5) family protein;
Ciclev10011647m.g	14655	15024	13970	2391	5937	2822	14413.594	3599.47847	-2.0015703	2.11E-05	0.00172957	HXXXD-type acyl-transferase family protein;
Ciclev10020494m.g	3298	5904	4658	1148	1386	969	4614.76458	1144.21169	-2.0119031	2.13E-05	0.00173412	Flavin-containing monooxygenase family protein;
Ciclev10033229m.g	1	0	2	349	344	27	1.01556018	227.634705	7.80830099	2.14E-05	0.00173794	
Ciclev10011292m.g	323	193	259	6	55	88	254.663577	50.4571655	-2.3354616	2.20E-05	0.0017769	Major facilitator superfamily protein;
Ciclev10015966m.g	720	597	832	50	175	274	713.101281	168.42575	-2.0819943	2.22E-05	0.0017926	Basic-leucine zipper (bZIP) transcription factor family protein;
Ciclev10023776m.g	152	148	149	27	37	6	148.362794	22.1770987	-2.7419868	2.32E-05	0.0018658	RING/U-box superfamily protein;
Ciclev10031163m.g	0	0	1	61	13	56	0.35448907	44.0233491	6.95638397	2.35E-05	0.00187967	glycerol-3-phosphate acyltransferase 1;

ID	R19T23	R19T24	R20T24	R20T18	R20T17	R19T17	baseMeanA	baseMeanB	2FoldChange	pval	padj	FuncAnn
Ciclev10026128m.g	7403	6125	4512	1032	1675	1906	5894.61296	1532.9538	-1.9430829	2.36E-05	0.00187967	uncoupling protein 5;
Ciclev10016471m.g	1032	633	383	57	90	150	661.494454	100.075424	-2.7246413	2.43E-05	0.00193324	Thymidine kinase;
Ciclev10016123m.g	2960	2457	2225	440	461	1032	2508.74848	656.058909	-1.9350706	2.54E-05	0.00201314	xyloglucan endotransglucosylase/hydrolase 5;
g.79523	44	26	11	0	0	0	25.9871639	0	#NAME?	2.62E-05	0.00206837	Retrovirus-related Pol polyprotein from transposon TNT 1-94
Ciclev10033874m.g	353	533	360	76	112	85	412.102095	89.4310356	-2.2041543	2.73E-05	0.00214454	GroES-like zinc-binding dehydrogenase family protein;
Ciclev10014545m.g	90	93	142	20	18	5	108.684839	13.7731574	-2.9802195	2.79E-05	0.00218917	Protein of unknown function (DUF594);
Ciclev10013532m.g	30	33	34	153	473	350	32.1631568	319.832889	3.31383738	2.91E-05	0.00227211	Eukaryotic protein of unknown function (DUF914);
Ciclev10030320m.g	12	15	24	129	85	145	17.1472072	120.159376	2.8089037	2.97E-05	0.00230891	receptor like protein 13;
Ciclev10023311m.g	0	0	0	18	20	46	0	28.5522463	Inf	3.23E-05	0.00250858	Leucine-rich repeat protein kinase family protein;
Ciclev10001544m.g	515	367	281	23	60	173	378.867716	88.2310503	-2.1023358	3.29E-05	0.00254687	CYCLIN A3;4;
Ciclev10020602m.g	16	27	50	222	153	155	31.5586391	174.64943	2.46835503	3.40E-05	0.00262189	MATE efflux family protein;
Ciclev10027095m.g	226	252	321	70	64	29	266.414682	52.6670376	-2.3387014	3.57E-05	0.00274037	Ankyrin repeat family protein;
Ciclev10011860m.g	1327	894	965	203	321	273	1044.56123	262.119796	-1.9945989	3.64E-05	0.00278794	Protein of unknown function (DUF1262);
Ciclev10012379m.g	7825	8630	4688	1847	1113	2651	6914.78166	1896.69593	-1.8661953	3.69E-05	0.00281091	plasma membrane intrinsic protein 2;
Ciclev10014457m.g	495	419	129	6	35	33	336.050085	24.5035016	-3.7776164	3.70E-05	0.00281091	Zinc finger (C3HC4-type RING finger) family protein;
Ciclev10004897m.g	156	234	232	923	928	690	207.451829	831.190212	2.00240229	3.72E-05	0.00281828	P-loop containing nucleoside triphosphate hydrolases superfamily protein;
Ciclev10019118m.g	597	233	277	8	43	61	358.275821	37.7043414	-3.2482681	3.87E-05	0.00291903	basic helix-loop-helix (bHLH) DNA-binding superfamily protein;
Ciclev10023230m.g	14	19	14	125	75	134	15.5382767	111.847127	2.84762979	3.90E-05	0.00293465	
Ciclev10026582m.g	11737	8603	8619	2564	3610	1582	9498.69827	2501.19059	-1.9251148	4.00E-05	0.00299937	early nodulin-like protein 17;
Ciclev10011664m.g	40	72	66	3	5	6	59.4698885	4.66201052	-3.6731352	4.14E-05	0.00309516	F-box/RNI-like superfamily protein;
Ciclev10030383m.g	4	6	6	63	47	78	5.33745661	62.9461206	3.55989313	4.40E-05	0.00328074	rna processing factor 2;
Ciclev10004905m.g	12305	7218	7435	2161	2606	2508	8795.1037	2400.21666	-1.8735359	4.43E-05	0.00328916	Exostosin family protein;
Ciclev10026223m.g	16645	20566	10386	1635	4352	6447	15585.9373	4183.14394	-1.8975854	4.55E-05	0.00336317	HAD superfamily, subfamily IIIB acid phosphatase ;
Ciclev10028347m.g	18	15	17	89	137	118	16.505276	113.175398	2.77756121	4.56E-05	0.00336317	cytochrome BC1 synthesis;
Ciclev10020041m.g	108	110	113	386	443	585	109.545016	471.439454	2.10554865	4.71E-05	0.0034593	
Ciclev10028735m.g	3173	1721	2315	492	1035	394	2362.56001	616.525636	-1.9381182	4.96E-05	0.00363388	CYCLIN D1;1;
Ciclev10023313m.g	63	92	70	2	5	17	74.5532119	8.31360905	-3.1647237	5.01E-05	0.003659	Protein kinase superfamily protein;
Ciclev10008128m.g	824	411	467	68	87	226	554.087291	130.216853	-2.0891971	5.06E-05	0.00368643	cyclin a2;1;
Ciclev10014629m.g	3359	1767	2723	281	1016	805	2579.42796	690.742419	-1.9008314	5.11E-05	0.00370791	Tetratricopeptide repeat (TPR)-like superfamily protein;
Ciclev10018400m.g	127	156	152	23	22	29	144.407303	24.6390331	-2.5511262	5.15E-05	0.00372371	
Ciclev10004465m.g	4924	5232	2913	679	705	2157	4272.45384	1214.52896	-1.814668	5.16E-05	0.00372371	Glycosyl hydrolases family 32 protein;
Ciclev10012479m.g	214	181	119	24	40	30	167.649277	30.7887169	-2.4449726	5.33E-05	0.0038219	NAD(P)-binding Rossmann-fold superfamily protein;
Ciclev10015894m.g	1671	1084	1257	255	376	420	1316.36908	348.862456	-1.9158338	5.34E-05	0.0038219	
Ciclev10012772m.g	1478	1442	929	342	466	198	1259.31657	324.206067	-1.957658	5.35E-05	0.0038219	ribosomal protein 5A;
Ciclev10025171m.g	75	100	111	467	301	557	95.4118402	444.554784	2.220121	5.37E-05	0.0038219	Leucine-rich repeat protein kinase family protein;
Ciclev10022946m.g	147	34	59	1	1	1	77.2261818	0.99014838	-6.2853015	5.39E-05	0.0038219	Bifunctional inhibitor/lipid-transfer protein/seed storage 2S albumin superfamily protein;
Ciclev10002428m.g	2510	2536	1583	182	453	1142	2169.3298	609.515657	-1.8315142	5.48E-05	0.00387754	DUTP-PYROPHOSPHATASE-LIKE 1;
Ciclev10006981m.g	23	17	33	0	0	0	24.3714093	0	#NAME?	5.50E-05	0.00387935	glutathione S-transferase TAU 8;
Ciclev10020402m.g	0	1	0	28	36	23	0.330699	28.3445693	6.42140984	5.63E-05	0.00395895	
Ciclev10029484m.g	4758	1543	1045	55	11	2	2339.42701	21.9434878	-6.7362186	5.66E-05	0.00396757	
Ciclev10020875m.g	15857	16868	13125	5552	4317	2813	15092.3713	4132.27435	-1.8688116	5.79E-05	0.00404365	O-methyltransferase 1;

ID	R19T23	R19T24	R20T24	R20T18	R20T17	R19T17	baseMeanA	baseMeanB	2FoldChange	pval	padj	FuncAnn
g.52133	221	341	279	1320	1935	724	279.425442	1278.18349	2.19355966	6.13E-05	0.0042661	Jasmonate O-methyltransferase
Ciclev10030632m.g	656	580	689	2644	2261	2184	637.166211	2335.41163	1.87393519	6.74E-05	0.00467704	Leucine-rich repeat receptor-like protein kinase family protein;
Ciclev10013891m.g	23	10	10	332	340	804	13.9032678	501.794909	5.17360192	6.81E-05	0.00471137	Plant protein of unknown function (DUF247);
Ciclev10006539m.g	15	28	28	0	0	0	23.7839967	0	#NAME?	6.91E-05	0.0047709	
Ciclev10003117m.g	0	0	0	14	38	20	0	23.3152498	Inf	7.42E-05	0.00509556	MEI2 C-terminal RRM only like 1;
Ciclev10013350m.g	4	2	0	59	28	37	1.8877262	41.0567409	4.44289768	7.43E-05	0.00509556	sugar transporter 1;
Ciclev10013396m.g	3	2	3	35	72	31	2.64461134	44.4386889	4.07068863	7.57E-05	0.00517741	Protein of unknown function (DUF581);
g.42881	5	7	3	50	95	32	4.91127046	56.6576456	3.52810247	7.67E-05	0.00522604	
Ciclev10032762m.g	4	12	10	156	377	106	8.73960689	203.461088	4.54104071	7.69E-05	0.00522604	MATE efflux family protein;
g.25137	60	40	23	231	217	390	39.7761315	281.956991	2.82550024	7.81E-05	0.00529308	
Ciclev10024444m.g	0	1	0	23	17	42	0.330699	27.8212879	6.39452669	7.88E-05	0.00532041	
Ciclev10019047m.g	202	121	153	16	32	42	156.18098	30.0979379	-2.3754822	8.93E-05	0.00601823	SEUSS-like 2;
Ciclev10014891m.g	173	161	152	10	35	48	160.163572	31.2268112	-2.3586889	9.07E-05	0.00609188	ATP binding;protein kinases;protein serine/threonine kinases;
Ciclev10028904m.g	33840	11250	30738	3218	4360	1177	24991.3851	2795.03373	-3.1604933	9.46E-05	0.00633313	NmrA-like negative transcriptional regulator family protein;
Ciclev10015223m.g	261	257	297	59	67	59	270.290811	60.8467711	-2.1512599	9.53E-05	0.00636515	ARM repeat superfamily protein;
Ciclev10015086m.g	22102	11748	10419	1105	2938	5401	14354.5499	3203.52493	-2.163776	0.00010095	0.00670767	O-Glycosyl hydrolases family 17 protein;
Ciclev10033033m.g	274	415	277	956	1165	1414	319.437039	1175.59013	1.87978165	0.00010108	0.00670767	
Ciclev10008234m.g	95	47	27	0	0	9	54.2393524	3.25368362	-4.0591941	0.00010276	0.00679942	Transducin/WD40 repeat-like superfamily protein;
Ciclev10030662m.g	260	267	207	17	35	113	241.387203	57.0015186	-2.0822769	0.00011102	0.0073244	DEAD/DEAH box RNA helicase family protein ;
Ciclev10009892m.g	29856	54216	34471	8471	16787	6144	39302.0834	10070.2123	-1.9645117	0.00011326	0.00745021	Bifunctional inhibitor/lipid-transfer protein/seed storage 2S albumin superfamily protein;
Ciclev10031464m.g	2293	1883	1035	283	857	329	1692.59504	471.052127	-1.8452782	0.00011442	0.00750498	HXXXD-type acyl-transferase family protein;
Ciclev10016248m.g	19	9	7	137	147	42	11.2827734	104.340911	3.20911129	0.00011487	0.0075125	Leucine-rich repeat transmembrane protein kinase;
Ciclev10028716m.g	14725	9136	10120	1716	6030	2209	11123.1161	3186.63218	-1.8034566	0.00011917	0.0077707	Pathogenesis-related thaumatin superfamily protein;
Ciclev10031299m.g	252	290	252	716	999	1293	262.492631	1003.4346	1.93459776	0.0001202	0.00781565	beta glucosidase 40;
Ciclev10028372m.g	43608	29035	25394	7912	9160	11866	31973.1708	9642.27626	-1.7294162	0.00012471	0.00808549	O-Glycosyl hydrolases family 17 protein;
Ciclev10024115m.g	808	576	579	181	293	52	643.450089	166.572959	-1.949674	0.00012606	0.00814937	
Ciclev10020328m.g	20731	15392	14077	3926	7511	3384	16436.0141	4779.43346	-1.7819489	0.00012792	0.0082457	FASCICLIN-like arabinogalactan-protein 10;
Ciclev10002052m.g	123	312	263	33	19	49	234.118305	34.2101938	-2.7747396	0.00012921	0.00830524	LRR and NB-ARC domains-containing disease resistance protein;
g.26568	4	13	5	54	81	131	7.29786057	89.4996398	3.61633638	0.00013245	0.00848904	
Ciclev10017986m.g	35	15	17	0	0	0	21.7171708	0	#NAME?	0.0001341	0.00857037	Protein of unknown function (DUF604);
Ciclev10012737m.g	3	1	0	18	52	34	1.25044514	33.9260682	4.76188057	0.00013754	0.00876577	Protein of unknown function (DUF567);
Ciclev10022832m.g	76	80	33	20	0	0	61.4542951	6.50251785	-3.2404435	0.00013869	0.00881382	Bifunctional inhibitor/lipid-transfer protein/seed storage 2S albumin superfamily protein;
Ciclev10011511m.g	1773	1751	1781	855	663	9	1753.96895	482.458205	-1.8621473	0.00013941	0.00881949	cytochrome P450, family 76, subfamily G, polypeptide 1;
Ciclev10024158m.g	0	0	0	17	21	52	0	30.6997449	Inf	0.00013988	0.00881949	
g.46803	179	214	346	17	20	76	248.30099	39.0727325	-2.667856	0.00013995	0.00881949	
Ciclev10032615m.g	15208	7757	9045	2769	4326	2411	10434.0855	3084.84931	-1.7580322	0.00014086	0.00883589	auxin-responsive family protein;
Ciclev10029978m.g	83	36	59	291	167	309	58.2663289	257.006287	2.14106933	0.000141	0.00883589	Protein kinase family protein with leucine-rich repeat domain;
Ciclev10020092m.g	994	2220	2047	13058	9592	4909	1764.53346	8931.38956	2.33959786	0.00014429	0.00901714	UDP-glucosyltransferase 74F2;
Ciclev10018937m.g	388	318	294	66	101	81	328.335903	81.3951721	-2.0121574	0.00014855	0.0092572	Protein kinase superfamily protein;
Ciclev10033897m.g	46	38	59	6	5	0	47.5841912	3.46826578	-3.778196	0.00015019	0.00930946	
Ciclev10013777m.g	238	222	198	68	56	24	216.570541	47.7811671	-2.180323	0.00015021	0.00930946	

ID	R19T23	R19T24	R20T24	R20T18	R20T17	R19T17	baseMeanA	baseMeanB	2FoldChange	pval	padj	FuncAnn
Ciclev10023075m.g	0	0	0	32	67	9	0	33.9923519	Inf	0.00015446	0.00954647	
Ciclev10010842m.g	85	84	80	20	11	4	82.1973156	11.2871224	-2.8644136	0.00015529	0.00957157	
Ciclev10022964m.g	83	65	65	282	368	233	69.9835344	287.608523	2.039019	0.00015588	0.00957828	cationic amino acid transporter 3;
Ciclev10018902m.g	25	12	15	113	81	121	16.9502752	105.066863	2.63192713	0.00015625	0.00957828	receptor-like protein kinase 1;
Ciclev10029921m.g	27	8	42	0	0	0	25.811848	0	#NAME?	0.0001569	0.00959151	2-oxoglutarate (2OG) and Fe(II)-dependent oxygenase superfamily protein;
Ciclev10023033m.g	669	683	377	175	262	30	564.613187	147.26019	-1.9388954	0.00015878	0.00968042	Lactoylglutathione lyase / glyoxalase I family protein;
Ciclev10019432m.g	19	12	15	131	113	50	15.1107829	94.9632477	2.65179086	0.00016022	0.00974172	C2 domain-containing protein / GRAM domain-containing protein;
Ciclev10011627m.g	8315	5788	5314	1151	2175	2486	6347.07045	1933.07666	-1.715192	0.00016198	0.00982231	IQ calmodulin-binding motif family protein;
Ciclev10013429m.g	155	281	117	23	29	28	181.921858	26.4020273	-2.7845983	0.00016299	0.00984255	ubiquitin 7;
Ciclev10005368m.g	11	9	17	129	156	42	12.3750077	104.471422	3.07760703	0.00016319	0.00984255	Protein kinase superfamily protein;
Ciclev10031503m.g	82	101	85	481	1105	429	88.6718978	646.847612	2.86687701	0.00016781	0.010092	MATE efflux family protein;
Ciclev10028964m.g	237	496	162	20	7	26	294.11388	18.0265629	-4.0281786	0.00016822	0.010092	basic chitinase;
Ciclev10027614m.g	121	182	170	18	38	41	157.546788	32.2076818	-2.2903036	0.00017009	0.01017689	Leucine-rich repeat transmembrane protein kinase;
Ciclev10020202m.g	0	0	0	13	27	54	0	31.9432946	Inf	0.00017848	0.01063296	Actin-like ATPase superfamily protein;
Ciclev10031335m.g	51	65	89	422	513	168	68.6806465	353.635124	2.36428605	0.00017866	0.01063296	Protein of unknown function (DUF1336);
Ciclev10011796m.g	6	3	7	150	37	109	5.31301275	99.4041849	4.22570451	0.00018151	0.01074975	HXXXD-type acyl-transferase family protein;
Ciclev10005701m.g	23283	15515	5899	1090	2647	400	14360.0758	1302.3654	-3.4628572	0.00018158	0.01074975	ethylene-responsive element binding protein;
Ciclev10010410m.g	104	69	166	12	17	10	113.547949	12.6762502	-3.1631017	0.00018351	0.01083583	indoleacetic acid-induced protein 16;
Ciclev10013304m.g	2604	1596	1510	648	642	400	1861.41375	550.138078	-1.7585331	0.0001866	0.01098916	Low temperature and salt responsive protein family;
Ciclev10016587m.g	737	692	400	88	124	295	596.590306	172.893856	-1.7868539	0.00019326	0.01135203	high-mobility group box 6;
Ciclev10019909m.g	90	20	44	1	1	3	49.8038831	1.71318919	-4.8615019	0.00019714	0.01154984	alpha/beta-Hydrolases superfamily protein;
Ciclev10020979m.g	264	244	258	24	42	120	253.086396	63.9325573	-1.9850073	0.00020292	0.01185726	DP-E2F-like 1;
Ciclev10018540m.g	311	224	254	718	840	1188	259.463816	917.868381	1.82275393	0.00021015	0.01224833	LRR and NB-ARC domains-containing disease resistance protein;
Ciclev10017187m.g	31	50	70	0	4	10	50.8532282	4.82921237	-3.3964795	0.00021826	0.01268763	ketol-acid reductoisomerase;
g.8136	959	665	345	96	193	142	636.225748	141.123885	-2.1725766	0.00021951	0.01272754	Shikimate O-hydroxycinnamoyltransferase
Ciclev10028588m.g	4097	1882	1729	412	400	568	2491.35376	460.69629	-2.435042	0.00022337	0.0129183	
Ciclev10031944m.g	85	65	23	12	0	6	55.7081577	6.07063312	-3.1979697	0.00022428	0.01293749	myb domain protein 105;
Ciclev10023570m.g	197	73	134	34	26	20	132.039225	26.1757426	-2.3346642	0.00022733	0.0130799	cellulose synthase like G3;
Ciclev10009186m.g	2219	1465	1418	532	637	363	1667.4451	497.529709	-1.7447847	0.00022951	0.01315952	non-intrinsic ABC protein 3;
Ciclev10024382m.g	0	0	0	53	38	163	0	87.6925771	Inf	0.00022988	0.01315952	
Ciclev10010532m.g	99	95	146	586	745	1250	113.523432	868.53333	2.93559111	0.00023249	0.0132453	receptor kinase 2;
Ciclev10024240m.g	56	69	66	5	12	6	63.3831042	7.4367769	-3.0913489	0.00023256	0.0132453	Leucine-rich repeat receptor-like protein kinase family protein;
Ciclev10001630m.g	5	1	5	89	52	25	3.63605456	53.7563229	3.8859887	0.00023488	0.01334391	UDP-glucosyl transferase 85A2;
g.81787	2	15	20	97	51	98	12.6634305	82.4448174	2.70276053	0.00023637	0.01339444	
Ciclev10026534m.g	481	247	370	60	189	42	360.309573	92.0533046	-1.9686956	0.00024392	0.01378778	
Ciclev10011856m.g	392	171	235	94	79	16	260.034622	60.3228251	-2.1079278	0.00024566	0.01385124	growth-regulating factor 4;
g.80361	20	23	38	2	0	0	27.2083025	0.65025178	-5.3869047	0.00024991	0.01405582	Histone acetyltransferase KAT6A
Ciclev10030229m.g	241	200	137	47	46	40	188.591076	43.702829	-2.1094628	0.00025717	0.01442756	ROTUNDIFOLIA like 5;
Ciclev10030861m.g	88	38	51	11	4	5	57.6247246	6.59799517	-3.1265883	0.00026503	0.01483174	Cellulose-synthase-like C6;
Ciclev10030800m.g	127	52	127	26	4	23	101.152379	17.9822508	-2.4918847	0.00027063	0.01510773	mechanosensitive channel of small conductance-like 10;
Ciclev10011372m.g	2606	2611	2381	946	803	595	2506.44638	766.385908	-1.7095004	0.00027227	0.01512775	senescence-associated gene 101;

ID	R19T23	R19T24	R20T24	R20T18	R20T17	R19T17	baseMeanA	baseMeanB	2FoldChange	pval	padj	FuncAnn
Ciclev10030238m.g	25	12	11	266	83	280	15.5323189	212.899873	3.77682995	0.00027234	0.01512775	lectin protein kinase family protein;
Ciclev10025594m.g	821	513	428	223	130	150	573.073769	166.186406	-1.7859185	0.00027536	0.01525827	Senescence/dehydration-associated protein-related;
Ciclev10033314m.g	77	87	85	11	11	17	82.5092015	13.0607546	-2.6593168	0.00028676	0.01585056	PSF2;
Ciclev10012518m.g	10962	9083	9181	1869	3131	4220	9619.05556	3083.54142	-1.641307	0.00028793	0.01587655	expansin A4;
Ciclev10006097m.g	1955	966	1483	307	785	251	1444.53042	428.804407	-1.7522089	0.0002904	0.01597368	glycine-rich protein;
Ciclev10013004m.g	337	446	396	88	163	72	391.187576	104.111387	-1.9097327	0.00029732	0.01631435	Plant invertase/pectin methylesterase inhibitor superfamily;
Ciclev10032130m.g	523	325	356	1284	1426	1255	394.017694	1303.96373	1.72657141	0.00031112	0.01703003	ELMO/CED-12 family protein;
Ciclev10012119m.g	761	970	643	1867	3026	2723	782.023441	2509.82741	1.6823044	0.00032785	0.01788312	Eukaryotic protein of unknown function (DUF914);
g.61688	19	21	20	95	107	120	19.8595193	106.744131	2.42625415	0.0003283	0.01788312	
Ciclev10004484m.g_Cic	2	7	11	343	220	56	6.82743684	198.533783	4.86189664	0.00033726	0.01830955	Copper amine oxidase family protein;Copper amine oxidase family protein;
g.16894	346	336	379	52	102	136	351.543609	97.0305339	-1.857193	0.00033775	0.01830955	
Ciclev10004390m.g	46	14	18	139	107	137	25.1133634	127.195517	2.34052066	0.00033936	0.01835213	phosphate 1;
Ciclev10030702m.g	656	581	835	2408	2768	1467	689.252314	2153.34735	1.64347695	0.00034391	0.01855383	Protein of unknown function (DUF1666);
Ciclev10027294m.g	11	19	28	0	0	0	19.5813774	0	#NAME?	0.00034488	0.01856186	alpha/beta-Hydrolases superfamily protein;
Ciclev10006135m.g	88	125	100	512	331	295	103.765502	373.572166	1.84806008	0.00034712	0.01859643	histidine-containing phosphotransmitter 1;
Ciclev10007588m.g	901	1091	888	77	182	616	951.809328	302.968641	-1.6515041	0.00034718	0.01859643	Minichromosome maintenance (MCM2/3/5) family protein;
Ciclev10021117m.g	43	105	81	412	216	242	76.6200378	286.996256	1.90523827	0.00035334	0.01888141	NAC domain containing protein 1;
Ciclev10033182m.g	75	103	80	23	7	13	85.4147762	14.3021754	-2.5782511	0.00036089	0.01923923	basic helix-loop-helix (bHLH) DNA-binding superfamily protein;
Ciclev10021099m.g	137	72	54	8	13	23	84.9544782	14.8615035	-2.5151099	0.00036371	0.01931394	RING/FYVE/PHD zinc finger superfamily protein;
Ciclev10024384m.g	0	0	0	243	13	98	0	118.380118	Inf	0.00036484	0.01931394	purple acid phosphatase 17;
Ciclev10020738m.g	482	567	631	1929	2360	1102	558.961482	1741.82825	1.6397816	0.00036487	0.01931394	C2H2-like zinc finger protein;
Ciclev10019324m.g	1024	986	622	112	198	489	860.501433	273.29099	-1.6547397	0.00036755	0.0194105	Minichromosome maintenance (MCM2/3/5) family protein;
Ciclev10023176m.g	10343	6612	6206	2667	3153	1643	7557.51906	2418.03085	-1.6440801	0.00037344	0.01967499	
Ciclev10003632m.g	17	50	42	0	7	0	36.6353858	2.1245146	-4.1080326	0.00037731	0.01983245	ralf-like 34;
Ciclev10027279m.g	36	74	48	273	284	106	52.5241551	213.275124	2.02166274	0.00037828	0.0198373	LRR and NB-ARC domains-containing disease resistance protein;
Ciclev10033878m.g	0	2	1	964	824	5140	1.01588707	2421.72195	11.2190775	0.00038315	0.02004568	EF-TU receptor;
Ciclev10004922m.g	7351	5169	5104	1750	2606	1324	5772.37997	1838.54976	-1.650598	0.00038443	0.02006618	Protein of unknown function (DUF819);
Ciclev10005700m.g	192	82	69	14	13	14	110.440817	13.5585752	-3.025996	0.00038751	0.02018027	UV-B-insensitive 4-like;
Ciclev10022129m.g	7	96	16	582	235	268	39.5650038	357.433727	3.17537897	0.00040372	0.0209757	Kunitz family trypsin and protease inhibitor protein;
Ciclev10027381m.g	69	68	48	10	10	4	60.6571687	7.73236139	-2.9716972	0.0004093	0.02121663	Leucine-rich repeat protein kinase family protein;
Ciclev10031441m.g	523	308	172	25	59	85	323.169823	56.7640046	-2.5092442	0.00041036	0.02122258	bZIP transcription factor family protein;
Ciclev10007213m.g	5371	4137	3859	1185	1867	1283	4382.72726	1415.74325	-1.6302692	0.000427	0.0220326	Glycoprotein membrane precursor GPI-anchored;
Ciclev10011268m.g	317	118	188	47	37	50	202.852936	44.5865142	-2.1857549	0.00042965	0.02211871	POX (plant homeobox) family protein;
Ciclev10018572m.g	191	71	57	410	365	632	102.242677	472.560771	2.20850236	0.00043307	0.02224357	disease resistance protein (TIR-NBS-LRR class), putative;
Ciclev10030779m.g	103	94	74	311	303	370	88.895848	326.837833	1.87838705	0.00043471	0.0222773	
Ciclev10029378m.g	19205	8728	11430	3534	2876	5328	12826.0591	3948.04761	-1.6998667	0.00043793	0.02237377	Pollen Ole e 1 allergen and extensin family protein;
Ciclev10011283m.g	620	619	693	195	123	265	640.444475	196.533212	-1.7043004	0.00043858	0.02237377	Ypt/Rab-GAP domain of gyp1p superfamily protein;
Ciclev10013452m.g	12	7	9	98	45	56	9.18427918	65.7650738	2.84008321	0.00044263	0.0225292	eukaryotic translation initiation factor 3A;
Ciclev10028756m.g	9717	6850	6681	2889	3603	1133	7612.68737	2442.40933	-1.6401008	0.00045585	0.02314957	EXORDIUM like 5;
Ciclev10017335m.g	1698	1756	1932	560	927	239	1786.15664	549.820309	-1.6998265	0.00046103	0.02335971	ribosomal protein S27;
Ciclev10017225m.g	3543	4680	4720	1301	2425	503	4307.07992	1340.82611	-1.6835879	0.00046613	0.02356546	
g.49641	1	1	0	36	30	11	0.63728105	24.7863191	5.28147047	0.00046893	0.0236534	Putative phagocytic receptor 1a

ID	R19T23	R19T24	R20T24	R20T18	R20T17	R19T17	baseMeanA	baseMeanB	2FoldChange	pval	padj	FuncAnn
Ciclev10010673m.g	4159	13630	12497	529	1999	769	10212.552	1056.70146	-3.2727037	0.00047085	0.02366627	
Ciclev10028503m.g	1506	1292	699	434	181	489	1136.76353	372.821992	-1.6083733	0.00047128	0.02366627	
Ciclev10024360m.g	24	13	16	264	154	79	17.3288812	161.132669	3.2169986	0.00047932	0.02401597	Class I glutamine amidotransferase-like superfamily protein;
Ciclev10010422m.g	110	221	200	547	465	755	177.706318	591.920237	1.73590781	0.00048348	0.02417092	Calcium-binding EF-hand family protein;
Ciclev10012862m.g	1716	1637	542	359	134	174	1259.58214	220.294025	-2.5154429	0.00048971	0.0244279	
Ciclev10011116m.g	1001	1083	719	210	397	271	919.913288	286.738794	-1.6817608	0.00049233	0.02450417	CRINKLY4 related 1;
Ciclev10013139m.g	6849	895	1598	9	9	11	2962.22957	9.63437623	-8.2642765	0.00049539	0.02460206	lipid transfer protein 1;
Ciclev10026696m.g	137	228	186	46	54	36	183.33608	44.3596382	-2.0471712	0.00050019	0.02477142	NAD(P)-binding Rossmann-fold superfamily protein;
Ciclev10019938m.g	16280	10592	8622	2963	5482	3325	11550.3243	3829.20179	-1.5928178	0.00050104	0.02477142	Eukaryotic aspartyl protease family protein;
Ciclev10033457m.g	175	298	232	43	52	91	234.441625	62.6608784	-1.9035918	0.0005021	0.02477142	cytochrome P450, family 82, subfamily C, polypeptide 4;
Ciclev10012328m.g	569	378	411	155	194	51	445.144414	127.711458	-1.8013855	0.0005056	0.02488938	Polynucleotidyl transferase, ribonuclease H-like superfamily protein;
Ciclev10008936m.g	552	288	210	64	62	93	338.917307	73.2465838	-2.2100999	0.00051016	0.02505893	geranylgeranyl reductase;
Ciclev10013412m.g	2	0	2	711	302	54	1.32214222	342.344241	8.01642656	0.00051797	0.02538106	Jojoba acyl CoA reductase-related male sterility protein;
Ciclev10033583m.g	74	69	81	17	9	10	74.218917	11.873863	-2.6439976	0.00051897	0.02538106	Leucine-rich repeat receptor-like protein kinase family protein;
Ciclev10023052m.g	32	29	39	155	124	158	33.2259702	145.148996	2.12715136	0.00052984	0.0258563	Ankyrin repeat family protein;
Ciclev10022991m.g_Cic	1	1	2	27	15	45	1.34625918	29.5993485	4.45853733	0.00053733	0.02607845	myb domain protein 15;myb domain protein 14;
Ciclev10028858m.g	110	123	131	366	415	466	120.83807	413.41795	1.77452598	0.00053735	0.02607845	AP2/B3-like transcriptional factor family protein;
Ciclev10019657m.g	4432	3792	4209	2023	1536	554	4104.82673	1324.19119	-1.6322099	0.00053787	0.02607845	cytochrome P450, family 78, subfamily A, polypeptide 6;
Ciclev10000162m.g	28	31	41	222	96	228	33.3700181	183.7408	2.46104764	0.00054131	0.02618875	receptor like protein 6;
Ciclev10012179m.g	4078	2199	2222	680	1023	1095	2765.1234	927.433081	-1.5760288	0.00054631	0.02637428	Peroxidase superfamily protein;
Ciclev10025209m.g	1182	771	690	120	571	150	861.946366	266.542858	-1.6932306	0.00060524	0.02915645	growth-regulating factor 1;
Ciclev10030734m.g	160	70	113	386	303	480	112.259322	390.98952	1.80029468	0.0006104	0.02934233	P-loop containing nucleoside triphosphate hydrolases superfamily protein;
Ciclev10008330m.g	90	47	59	6	16	7	64.0500922	9.33743154	-2.778103	0.00061524	0.02951193	alkenal reductase;
Ciclev10014934m.g	597	251	296	61	93	112	370.963695	88.5486585	-2.0667356	0.0006253	0.02993042	
Ciclev10022156m.g	0	0	0	68	63	0	0	41.2291921	Inf	0.00063036	0.03010854	Kunitz family trypsin and protease inhibitor protein;
Ciclev10027218m.g	624	580	559	126	314	107	581.272007	174.9482	-1.7322856	0.00063736	0.0303785	
Ciclev10030014m.g	0	1	0	140	76	10	0.330699	72.1989875	7.77031609	0.00063924	0.03040378	SAUR-like auxin-responsive protein family ;
Ciclev10011219m.g	11	8	7	73	56	135	8.499418	89.5355613	3.39702484	0.00064601	0.03066113	Wall-associated kinase family protein;
Ciclev10013919m.g	28	78	78	222	242	222	62.0289668	225.882982	1.86456158	0.00065289	0.03087966	Protein kinase superfamily protein;
Ciclev10026049m.g	4502	2932	2547	944	1357	1049	3252.7255	1098.00607	-1.566763	0.00065336	0.03087966	carboxyesterase 18;
Ciclev10009194m.g	718769	2E+06	789821	130563	353867	129718	1003248.48	196744.488	-2.3502838	0.00068142	0.03213818	Concanavalin A-like lectin protein kinase family protein;
Ciclev10025940m.g	180	205	161	1057	802	382	180.050804	725.167535	2.0099103	0.00068546	0.03226122	bZIP transcription factor family protein;
Ciclev10018374m.g	447	394	636	185	233	27	492.792628	140.625327	-1.8091242	0.00069517	0.03262977	Oxidoreductase, zinc-binding dehydrogenase family protein;
Ciclev10012556m.g	4626	3073	103	5	64	23	2470.99896	29.3647322	-6.3948661	0.00069619	0.03262977	myb-like HTH transcriptional regulator family protein;
Ciclev10022581m.g	48	107	129	1147	524	451	95.8298211	695.000193	2.8584668	0.00070806	0.03311688	SAUR-like auxin-responsive protein family ;
Ciclev10007176m.g	3	2	6	54	29	38	3.70807854	40.096134	3.43471943	0.00071264	0.03326176	F-box family protein with a domain of unknown function (DUF295);
Ciclev10014352m.g	7134	5661	5303	749	2968	2456	5939.0989	2032.20759	-1.5471963	0.00071853	0.03346727	Calcium-dependent lipid-binding (CaLB domain) plant phosphoribosyltransferase family protein;
g.57791	2	21	13	98	94	30	12.166201	71.2371456	2.54975096	0.00072251	0.03358309	
Ciclev10027783m.g	44	5	27	0	0	1	24.7143098	0.3615204	-6.0951257	0.00073965	0.03430914	Protein kinase family protein with leucine-rich repeat domain;

ID	R19T23	R19T24	R20T24	R20T18	R20T17	R19T17	baseMeanA	baseMeanB	2FoldChange	pval	padj	FuncAnn
Ciclev10024164m.g	104	189	103	503	424	375	130.899018	427.793359	1.70845981	0.00076064	0.03514308	Class I glutamine amidotransferase-like superfamily protein;
g.49628	5	28	19	0	0	0	17.5277746	0	#NAME?	0.00076075	0.03514308	Protein BONZAI 3
Ciclev10028301m.g	1059	586	672	114	210	425	756.676656	254.445961	-1.5723176	0.00076868	0.03537947	glycosyl hydrolase 9B18;
Ciclev10021017m.g	3169	1369	2591	739	1016	533	2342.76661	741.316528	-1.6600517	0.00076902	0.03537947	Heavy metal transport/detoxification superfamily protein ;
Ciclev10006682m.g	211	101	147	15	29	69	150.199304	38.6233566	-1.9593327	0.00077529	0.03559537	DNA repair (Rad51) family protein;
Ciclev10021975m.g	90	123	174	18	39	33	129.949459	29.6190207	-2.1333548	0.00078365	0.03586293	ABL interactor-like protein 2;
Ciclev10004407m.g	54	30	89	279	330	599	58.0259275	407.416533	2.81173495	0.00078431	0.03586293	pyruvate kinase family protein;
Ciclev10021342m.g	2117	2845	3330	7023	9460	7700	2770.32145	7938.19597	1.51875779	0.00079464	0.03626151	Duplicated homeodomain-like superfamily protein;
Ciclev10006304m.g	66	47	29	212	124	211	46.0574512	182.841753	1.98908907	0.00079755	0.03632084	F-box family protein;
Ciclev10032123m.g	4858	5893	6289	2020	2962	887	5667.56655	1876.39608	-1.594765	0.00080329	0.03650815	magnesium-protoporphyrin IX methyltransferase;
Ciclev10011063m.g	4346	2615	2428	9154	10141	7986	3057.88292	8941.119	1.54792223	0.00082125	0.0372495	starch branching enzyme 2.2;
Ciclev10031896m.g	23158	14956	10350	1577	3809	8193	15714.7232	4630.69963	-1.7628148	0.00083153	0.03763984	Leucine-rich repeat (LRR) family protein;
Ciclev10016312m.g	17628	12167	11547	4347	7173	2848	13521.3283	4619.95282	-1.5492869	0.00083439	0.03769341	FASCICLIN-like arabinoogalactan 7;
Ciclev10003998m.g	139	174	242	622	697	433	185.942885	570.307593	1.6168807	0.00083643	0.03770985	Protein of unknown function (DUF726);
Ciclev10013652m.g	12	6	25	71	99	68	14.5254052	77.7140322	2.41959671	0.00084208	0.03788858	Leucine-rich repeat protein kinase family protein;
Ciclev10030019m.g	111	166	172	34	77	0	149.898761	34.4239409	-2.1225043	0.00085063	0.038197	
Ciclev10028359m.g	7062	4408	3579	2236	2079	891	4891.51999	1680.07701	-1.5417555	0.00085394	0.03826925	arogenate dehydratase 6;
Ciclev10011938m.g	1268	798	554	161	299	388	849.030783	283.362308	-1.583169	0.00085814	0.03838082	squamosa promoter binding protein-like 9;
Ciclev10001212m.g	128634	142371	147008	7772	104325	34282	138631.551	46583.376	-1.5733685	0.00087257	0.0389488	Eukaryotic aspartyl protease family protein;
Ciclev10009711m.g	1237	816	664	219	439	240	884.473119	291.204883	-1.6027838	0.00087462	0.03896285	Calcium-binding EF-hand family protein;
Ciclev10006436m.g	31	32	45	0	0	9	36.0384195	3.25368362	-3.4693899	0.00087711	0.03899674	Major facilitator superfamily protein;
Ciclev10017244m.g	8784	11057	10125	2363	6659	1430	9938.75738	3306.26705	-1.5878622	0.00088151	0.03911506	Gibberellin-regulated family protein;
Ciclev10031461m.g	97	96	69	579	361	903	85.9453085	624.265068	2.86066795	0.00088668	0.03921814	
Ciclev10027290m.g	0	0	0	18	11	22	0	17.1442379	Inf	0.00088732	0.03921814	HXXXD-type acyl-transferase family protein;
Ciclev10032726m.g	6139	3745	3526	1816	2242	600	4370.5034	1487.79254	-1.5546261	0.00090467	0.03990679	prenylated RAB acceptor 1.B4;
Ciclev10013222m.g	165	103	101	400	392	410	120.451431	397.24654	1.72158312	0.00090959	0.03993685	
Ciclev10032534m.g	14515	14880	5353	1435	2354	4062	11268.4196	2649.49544	-2.0884956	0.00090993	0.03993685	tonoplast intrinsic protein 1;3;
Ciclev10027656m.g	0	0	0	19	14	55	0	30.3100433	Inf	0.00091068	0.03993685	
Ciclev10028504m.g	328	410	183	134	106	34	301.017002	88.0297844	-1.7737813	0.00093375	0.04086889	HXXXD-type acyl-transferase family protein;
Ciclev10029683m.g	1553	1562	2749	44	916	203	1967.1642	365.702091	-2.4273766	0.00094461	0.04126374	PHYTOSULFOKINE 3 PRECURSOR;
Ciclev10020772m.g	344	339	419	1671	1702	625	366.102105	1285.79617	1.81234398	0.0009465	0.04126627	
Ciclev10020637m.g	259	51	148	24	8	0	148.734781	10.2310381	-3.8617176	0.0009503	0.0413518	Oxidoreductase family protein;
Ciclev10031131m.g	394	490	339	111	92	178	403.007632	128.361798	-1.6505913	0.00095722	0.04157261	Transmembrane amino acid transporter family protein;
Ciclev10030080m.g	57	93	98	342	315	190	82.9701125	275.485089	1.73131058	0.00097062	0.0420184	S-adenosyl-L-methionine-dependent methyltransferases superfamily protein;
Ciclev10020633m.g	2788	2262	2640	866	1245	555	2538.64303	860.062943	-1.5615434	0.00097122	0.0420184	MAP kinase 4;
Ciclev10030744m.g	4134	4199	3000	704	1749	1517	3719.4825	1308.14023	-1.5075847	0.00101189	0.04369394	potassium transporter 2;
Ciclev10026932m.g	50	14	13	0	1	0	24.5672462	0.30350209	-6.3388859	0.0010303	0.04440343	
Ciclev10013721m.g	379	87	136	6	11	9	193.175922	8.54296192	-4.4990351	0.00103942	0.0447108	Plant protein of unknown function (DUF247);
Ciclev10015924m.g	5366	3718	2817	1400	1462	1330	3873.25386	1379.71843	-1.4891722	0.00106571	0.04568211	Peroxidase superfamily protein;
Ciclev10011084m.g	134	85	74	695	283	401	95.4236004	456.823267	2.2592181	0.00106606	0.04568211	
Ciclev10010236m.g	24	15	31	1682	1397	80	23.3076152	999.775797	5.4227313	0.00108354	0.04634278	multiprotein bridging factor 1C;

ID	R19T23	R19T24	R20T24	R20T18	R20T17	R19T17	baseMeanA	baseMeanB	2FoldChange	pval	padj	FuncAnn
Ciclev10012511m.g_Cic	11	12	7	127	65	215	9.82221402	138.74551	3.82024902	0.00109084	0.04656663	Glutathione S-transferase family protein;Glutathione S-transferase family protein;
Ciclev10019453m.g	1221	692	474	140	187	299	771.208207	210.367115	-1.8742112	0.00111043	0.04731322	Pyruvate kinase family protein;
Ciclev10013348m.g	544	272	248	86	157	65	344.644051	99.1094804	-1.7980121	0.00111625	0.04732358	3-oxo-5-alpha-steroid 4-dehydrogenase family protein;
Ciclev10022419m.g	6	19	14	91	107	29	13.0856203	72.5452711	2.47089926	0.00111743	0.04732358	Bifunctional inhibitor/lipid-transfer protein/seed storage 2S albumin superfamily protein;
Ciclev10032075m.g	254	191	166	53	59	59	199.880535	56.4679991	-1.8236326	0.0011178	0.04732358	Succinyl-CoA ligase, alpha subunit;
Ciclev10007993m.g	3016	1733	1349	7364	4138	5768	1975.95858	5735.36838	1.53733345	0.00111912	0.04732358	pectin methylesterase 44;
Ciclev10011408m.g	57	34	32	241	111	134	40.0625929	160.487806	2.00213598	0.00112119	0.04732358	galactosyltransferase1;
Ciclev10022798m.g	7334	5227	3734	2455	2531	807	5300.6986	1858.09481	-1.5123584	0.00113263	0.04769185	histone H2A 11;
Ciclev10001088m.g	2472	2135	1717	272	907	1037	2072.57092	738.607292	-1.4885421	0.0011357	0.04769185	Protein kinase superfamily protein;
Ciclev10016074m.g	89617	105995	87820	46229	52342	3125	93658.634	32045.9023	-1.5472721	0.00113628	0.04769185	chlorophyll A/B-binding protein 2;
Ciclev10030874m.g	224	275	258	578	701	986	251.074784	757.136845	1.59243694	0.00114933	0.04814994	ARF-GAP domain 1;
Ciclev10015161m.g	450	423	524	65	151	236	463.59987	152.280813	-1.606146	0.0011745	0.0489705	DNA repair metallo-beta-lactamase family protein;
Ciclev10023140m.g	23	18	26	0	2	0	22.2206848	0.60700417	-5.194053	0.00117471	0.0489705	spermidine synthase 2;
Ciclev10032171m.g	447	277	361	57	164	119	356.616352	111.327446	-1.6795635	0.00117545	0.0489705	Squamosa promoter-binding protein-like (SBP domain) transcription factor family protein;
Ciclev10000463m.g	2967	4341	4117	9470	9790	12767	3804.62479	10665.7586	1.48716049	0.00119377	0.04964196	S-adenosyl-L-methionine-dependent methyltransferases superfamily protein;
Ciclev10013199m.g	91	16	21	0	0	0	40.6344207	0	#NAME?	0.00120097	0.04984908	
Ciclev10016385m.g	73	99	46	1	25	14	71.4261879	12.9739637	-2.4608338	0.00122449	0.05073177	PLATZ transcription factor family protein;
Ciclev10001376m.g	281	227	184	33	58	111	226.444217	68.4610401	-1.7258006	0.00123723	0.0511653	TESMIN/TSO1-like CXC 2;
g.24760	0	0	0	31	35	143	0	72.3988932	Inf	0.00124607	0.05136973	Protein FAR1-RELATED SEQUENCE 5
Ciclev10020613m.g	335	571	922	6694	4905	1693	618.373035	4277.1245	2.79009188	0.00124674	0.05136973	NAD(P)-binding Rossmann-fold superfamily protein;
Ciclev10022148m.g	96	126	147	27	30	33	123.209844	29.8136349	-2.0470733	0.00125038	0.05142545	Calcium-binding EF-hand family protein;
Ciclev10000257m.g	289	165	153	32	60	80	197.404374	57.5357859	-1.7786225	0.00125272	0.05142765	putative protein kinase 1;
Ciclev10033790m.g	88	106	74	14	36	6	88.2655054	17.64696	-2.3224301	0.00125846	0.05156928	NAC domain containing protein 80;
Ciclev10022303m.g	629	1061	793	150	297	401	824.82158	283.878685	-1.5388076	0.00126475	0.05173288	myb domain protein 82;
Ciclev10022123m.g	207	185	112	49	61	26	164.344575	43.8443264	-1.9062618	0.00127237	0.05174138	homolog of carrot EP3-3 chitinase;
g.5568	21	14	14	0	0	0	16.030856	0	#NAME?	0.00127275	0.05174138	
Ciclev10028445m.g	10581	10125	5833	2996	5389	1190	8660.00677	3039.85919	-1.5103637	0.00127355	0.05174138	GTP binding Elongation factor Tu family protein;
Ciclev10011595m.g	549	429	367	77	102	258	440.280903	149.26417	-1.5605564	0.00127524	0.05174138	ammonium transporter 2;
Ciclev10031364m.g	9010	6522	5441	1636	3958	1963	6847.89815	2442.83177	-1.4871067	0.00127646	0.05174138	Eukaryotic aspartyl protease family protein;
Ciclev10026481m.g	260977	392112	410441	106414	202862	77000	355178.757	124004.058	-1.518158	0.00128919	0.05216336	germin-like protein 1;
Ciclev10000878m.g	2911	2815	1539	992	716	880	2368.93671	857.970333	-1.46524	0.00130506	0.05271099	Protein kinase superfamily protein;
Ciclev10025415m.g	3121	2606	1528	8600	5685	5929	2360.30346	6664.9465	1.49762094	0.00131232	0.05290892	
Ciclev10025811m.g	280	102	142	35	41	39	169.911719	37.9222874	-2.1636675	0.00135064	0.05435657	Calcium-dependent phosphotriesterase superfamily protein;
Ciclev10013908m.g	84	53	75	14	18	7	69.866619	12.5454429	-2.4774399	0.00135645	0.05449296	heteroglycan glucosidase 1;
g.22776	0	0	0	38	44	0	0	25.7088757	Inf	0.00136586	0.05477299	
Ciclev10020714m.g	469	270	227	150	71	82	313.544729	99.9622049	-1.6492166	0.00138691	0.05551842	Protein kinase superfamily protein;
Ciclev10030045m.g	48	42	63	5	8	9	50.9381075	7.30732977	-2.8013291	0.00140733	0.05623584	UDP-Glycosyltransferase superfamily protein;
Ciclev10031240m.g	87	98	129	4	28	41	104.81023	24.6208985	-2.0898242	0.0014122	0.05633026	Phototropic-responsive NPH3 family protein;
Ciclev10033347m.g	70	102	126	314	365	275	99.8576639	312.285902	1.64492238	0.00142329	0.05667213	Polymerase/histidinol phosphatase-like;

ID	R19T23	R19T24	R20T24	R20T18	R20T17	R19T17	baseMeanA	baseMeanB	2FoldChange	pval	padj	FuncAnn
Ciclev10023350m.g	1	1	1	15	30	60	0.99177012	35.6731751	5.16869006	0.00144553	0.0574558	
Ciclev10004792m.g	900	109	291	0	4	13	415.126351	5.91377357	-6.1333277	0.0014627	0.05796351	3-ketoacyl-CoA synthase 11;
Ciclev10032535m.g	2642	3564	3199	248	1679	1477	3122.61154	1124.17686	-1.4738841	0.00146345	0.05796351	RNA-binding (RRM/RBD/RNP motifs) family protein;
Ciclev10013846m.g	506	572	816	311	260	74	633.553423	206.777205	-1.6153891	0.00147362	0.05826356	Carbohydrate-binding X8 domain superfamily protein;
Ciclev10010132m.g	120	168	178	422	458	738	155.446332	543.009139	1.8045599	0.00149957	0.05909627	
Ciclev10023721m.g	117	36	30	0	4	3	58.4099356	2.29856955	-4.6674056	0.00149993	0.05909627	
Ciclev10016270m.g	2967	2857	2210	425	1620	864	2637.85682	942.205511	-1.4852526	0.00151264	0.05949285	expansin B3;
Ciclev10027007m.g	1233	1067	809	520	513	62	1017.65315	347.176299	-1.5515056	0.00153732	0.06033972	
Ciclev10011724m.g	198	157	149	54	28	58	165.44186	47.0230399	-1.8148846	0.00154084	0.06033972	Plant protein of unknown function (DUF247);
Ciclev10011218m.g	867	282	303	75	32	37	466.47394	47.4727636	-3.2966245	0.00154222	0.06033972	receptor lectin kinase;
Ciclev10028941m.g	3531	2618	2404	393	1139	1579	2800.50291	1044.30407	-1.4231441	0.00156538	0.06113961	fatty acid desaturase 3;
Ciclev10010682m.g	36	20	36	5	2	0	30.4125401	2.23263363	-3.7678479	0.00158207	0.06158022	Ankyrin repeat family protein;
Ciclev10007168m.g	107	122	205	60	22	19	145.819816	33.0534871	-2.1413124	0.00158472	0.06158022	cyclic nucleotide gated channel 1;
Ciclev10031899m.g	277	117	175	42	24	92	185.650597	54.1992146	-1.7762461	0.00158488	0.06158022	Serine/threonine-protein kinase WNK (With No Lysine)-related;
Ciclev10012020m.g	333	441	412	1216	907	1238	393.979577	1118.19174	1.50497483	0.00159617	0.06191203	F-box and associated interaction domains-containing protein;
Ciclev10006049m.g	9030	3940	4538	1147	2451	1527	5680.06134	1668.84467	-1.7670568	0.00159917	0.06192186	
Ciclev10011382m.g	2261	1137	1332	363	502	837	1541.36621	572.971323	-1.4276748	0.00161426	0.06239865	acyl activating enzyme 12;
g.15513	237	374	420	961	1205	771	345.22678	956.898226	1.47082111	0.00162157	0.06257363	
Ciclev10010161m.g_Cic	15	65	36	474	512	96	38.8557723	344.2087	3.14708272	0.00164214	0.06317657	O-Glycosyl hydrolases family 17 protein;
Ciclev10008811m.g	4647	2680	2947	999	1962	804	3355.63938	1210.93426	-1.4704671	0.00164281	0.06317657	
Ciclev10028174m.g	5194	6547	4235	2338	2165	1385	5258.73472	1917.93211	-1.4551641	0.0016524	0.06343681	polyol/monosaccharide transporter 5;
Ciclev10028241m.g	9196	4918	4048	1423	2912	1532	5880.67794	1900.30147	-1.6297542	0.00167736	0.06428538	O-Glycosyl hydrolases family 17 protein;
Ciclev10022791m.g	3173	6294	7417	123	1840	1793	5683.44977	1246.6404	-2.1887215	0.00168566	0.06449354	PHD finger protein;
Ciclev10023632m.g	0	0	0	8	14	24	0	15.526526	Inf	0.00170034	0.06494503	NAD(P)-binding Rossmann-fold superfamily protein;
Ciclev10016889m.g	1078	411	747	160	278	241	731.216069	223.52014	-1.7098929	0.00170829	0.06513786	ubiquitin-conjugating enzyme19;
Ciclev10001310m.g	6366	3963	4153	1524	2672	1157	4734.45455	1724.72854	-1.4568289	0.00171987	0.06546853	lysm domain GPI-anchored protein 1 precursor;
Ciclev10007806m.g	1054	636	488	115	295	351	706.452708	253.816254	-1.4768085	0.0017231	0.06548068	pectin methylesterase 3;
Ciclev10011513m.g	190	83	63	10	13	28	108.031417	17.3193573	-2.6409936	0.00172771	0.06554515	
Ciclev10013959m.g	44	14	13	3	0	0	22.727754	0.97537768	-4.5423504	0.00173597	0.06574765	Disease resistance protein (TIR-NBS-LRR class) family;
Ciclev10011488m.g	0	0	1	17	16	52	0.35448907	29.1822345	6.36320542	0.00176719	0.06681748	
Ciclev10020219m.g	519	524	392	1629	1379	984	471.362074	1303.89553	1.4679207	0.00180406	0.06805889	
Ciclev10031332m.g	5250	3104	2090	1057	1474	914	3376.9276	1121.44979	-1.5903462	0.00180607	0.06805889	Eukaryotic aspartyl protease family protein;
Ciclev10020910m.g	1353	667	737	304	276	402	896.640186	327.936049	-1.4511147	0.00181238	0.06818255	Lactate/malate dehydrogenase family protein;
Ciclev10006099m.g	117	141	98	308	490	291	117.238587	354.057234	1.5945351	0.00181649	0.06822309	MLP-like protein 423;
Ciclev10000604m.g	872	902	442	2965	2015	1332	722.314214	2057.10015	1.50991357	0.00182734	0.06851606	Mannose-binding lectin superfamily protein;
Ciclev10020672m.g	193	204	133	574	522	460	173.779978	511.349736	1.55704849	0.00183738	0.06877778	Leucine-rich repeat transmembrane protein kinase;
Ciclev10003684m.g	453	499	569	1133	1281	1790	505.604748	1404.27533	1.47374391	0.00187635	0.07012007	NAD(P)-binding Rossmann-fold superfamily protein;
Ciclev10017548m.g	543	1236	942	84	437	196	909.14672	230.798985	-1.9778763	0.001883	0.07025175	HXXXD-type acyl-transferase family protein;
Ciclev10005103m.g	48	127	178	354	329	391	119.813765	356.301229	1.57230379	0.00189041	0.0703011	Cation efflux family protein;
Ciclev10005466m.g	498	250	150	31	41	78	288.52597	50.7210796	-2.5080438	0.00189057	0.0703011	Homeodomain-like superfamily protein;
Ciclev10009160m.g	72	48	57	204	233	161	58.1533363	195.246453	1.74736247	0.00191043	0.07092245	RING/U-box superfamily protein;
Ciclev10011477m.g	76	145	148	499	291	325	123.715973	368.051058	1.57287414	0.0019388	0.07179399	Jojoba acyl CoA reductase-related male sterility protein;
g.70084	819	983	932	230	261	490	906.551626	331.137997	-1.4529566	0.00194112	0.07179399	

ID	R19T23	R19T24	R20T24	R20T18	R20T17	R19T17	baseMeanA	baseMeanB	2FoldChange	pval	padj	FuncAnn
Ciclev10027566m.g	17	21	17	1	0	0	18.182888	0.32512589	-5.8054391	0.00194348	0.07179399	HAD superfamily, subfamily IIIB acid phosphatase ;
Ciclev10006897m.g	205	82	117	34	35	39	131.441859	35.776149	-1.8773548	0.0019472	0.07181339	myb domain protein 18;
Ciclev10031511m.g	324	458	340	28	64	253	371.319009	119.99232	-1.6297171	0.00195372	0.07193599	DNA primase, large subunit family;
Ciclev10033895m.g	1543	626	507	6	80	263	859.799631	121.310788	-2.8252926	0.00196224	0.07213149	beta-ketoacyl reductase 2;
Ciclev10031869m.g	479	271	328	120	107	131	352.744644	118.849003	-1.5694944	0.0019779	0.07258862	phosphate transporter 3;1;
Ciclev10017313m.g	169	260	174	1230	867	410	199.475204	811.264521	2.02396297	0.00199806	0.07320889	
Ciclev10026265m.g	684	658	643	88	264	349	655.238534	234.90625	-1.4799351	0.00200832	0.07346549	phosphate starvation-induced gene 2;
Ciclev10011988m.g	17	28	17	0	1	1	20.497781	0.66502249	-4.9459208	0.0020128	0.07350952	MATE efflux family protein;
g.8481	382	213	204	87	128	39	259.868999	81.2335153	-1.6776376	0.00203205	0.07409244	
Ciclev10020787m.g	679	374	893	8	120	278	648.409373	139.523929	-2.2163924	0.00206653	0.07522777	Pectin lyase-like superfamily protein;
Ciclev10007028m.g	35	28	26	4	3	0	29.2066595	2.21100983	-3.72352	0.00207582	0.07544367	receptor-like protein kinase 1;
Ciclev10011875m.g	20735	10632	8171	4270	4487	2380	12769.5007	3610.51998	-1.8224236	0.00208521	0.07561295	Ribosomal protein L4/L1 family;
Ciclev10013114m.g	23660	4746	4910	7	123	354	10563.77	167.58486	-5.9780892	0.0020872	0.07561295	lipid transfer protein 6;
Ciclev10014531m.g	3260	4343	3188	749	1594	1711	3565.79439	1345.86303	-1.4056919	0.00209794	0.07588014	Cellulose-synthase-like C4;
Ciclev10031185m.g	54580	41663	23791	19058	13595	13010	38944.8101	15025.7405	-1.373995	0.00211157	0.07625033	calnexin 1;
Ciclev10033534m.g	17	18	28	79	89	105	21.0901707	90.6562734	2.10383609	0.00212503	0.0765297	P-loop containing nucleoside triphosphate hydrolases superfamily protein;
Ciclev10012705m.g	40	107	37	268	194	148	60.7641708	199.518163	1.71522729	0.00212611	0.0765297	Protein of unknown function (DUF567);
Ciclev10010597m.g	87	99	134	36	22	22	106.913374	26.3350269	-2.0213875	0.00214099	0.07694239	
Ciclev10017279m.g	15	28	26	312	233	63	23.0750185	194.93105	3.0785602	0.00214657	0.07701973	
Ciclev10015508m.g	1559	838	619	104	247	438	974.515908	267.124044	-1.8671759	0.00219333	0.07857219	Leucine-rich repeat (LRR) family protein;
Ciclev10025924m.g	189	74	100	19	50	26	117.86464	30.7520267	-1.9383776	0.00220801	0.07897238	CYCLIN D4;1;
Ciclev10006285m.g	257	428	427	128	187	61	371.697591	120.423749	-1.6260094	0.00222137	0.07927669	RNA polymerase Rpb7-like, N-terminal domain;
Ciclev10022365m.g	11	10	14	222	192	32	11.6422395	142.019001	3.60864347	0.00222357	0.07927669	homeobox 7;
Ciclev10008876m.g	701	451	446	131	185	245	522.161389	187.311876	-1.4790534	0.00223324	0.07949554	Galactosyltransferase family protein;
Ciclev10001482m.g	1590	1734	1904	218	1285	448	1735.84471	622.838765	-1.4787072	0.00225333	0.08008409	heat shock transcription factor B4;
Ciclev10021324m.g	515	616	916	2168	2093	1251	686.312325	1792.36482	1.38492715	0.00225718	0.08009444	Galactose mutarotase-like superfamily protein;
Ciclev10032065m.g	747	493	372	140	250	177	523.92133	185.382258	-1.498847	0.00227048	0.08043941	growth-regulating factor 5;
Ciclev10013514m.g	28	10	26	204	84	88	21.108003	123.633653	2.55020949	0.00230566	0.0815573	NAD(P)-binding Rossmann-fold superfamily protein;
Ciclev10005315m.g	156	180	122	1034	570	389	150.600286	649.807798	2.10928855	0.00232762	0.08220483	nodulin MtN21 /EamA-like transporter family protein;
Ciclev10006759m.g	40	75	58	8	10	13	57.626073	10.3357932	-2.4790726	0.00234279	0.08261087	NagB/RpiA/CoA transferase-like superfamily protein;
Ciclev10003332m.g	0	0	1	18	23	13	0.35448907	17.5325793	5.62815331	0.0023488	0.08269315	N-terminal nucleophile aminohydrolases (Ntn hydrolases) superfamily protein;
Ciclev10000783m.g	44481	33895	27440	4193	18079	18408	34573.2987	13505.1346	-1.3561502	0.0023552	0.08274179	SKU5 similar 5;
Ciclev10013926m.g	129	219	186	19	44	97	177.907132	54.5989627	-1.7041789	0.00235754	0.08274179	NmrA-like negative transcriptional regulator family protein;
Ciclev10019700m.g	131	82	35	23	11	11	79.6866837	14.7931429	-2.4294101	0.00237457	0.08317822	alpha/beta-Hydrolases superfamily protein;
Ciclev10021731m.g	328	394	465	83	130	195	395.691734	136.937199	-1.5308625	0.00237737	0.08317822	18S pre-ribosomal assembly protein gar2-related;
Ciclev10001186m.g	2234	1208	969	529	572	501	1427.88859	526.716512	-1.4387848	0.00238312	0.08325009	
Ciclev10029171m.g	1	1	0	40	18	8	0.63728105	21.3602365	5.06685404	0.00239968	0.08369858	CSL zinc finger domain-containing protein;
Ciclev10022844m.g	4457	3361	3271	1732	2023	476	3637.44927	1349.18648	-1.4308374	0.00244435	0.08512476	B-cell receptor-associated 31-like;
Ciclev10031216m.g	170	200	190	843	436	1055	185.611671	787.812061	2.08556407	0.00246968	0.08587395	cytochrome P450, family 82, subfamily C, polypeptide 4;
Ciclev10022041m.g	93229	195242	107816	44792	49672	20744	131368.266	37137.9738	-1.8226498	0.0024809	0.08599308	Kunitz family trypsin and protease inhibitor protein;
Ciclev10001754m.g	136	152	148	445	493	326	144.425789	412.163202	1.51288733	0.0024815	0.08599308	Protein kinase superfamily protein;

ID	R19T23	R19T24	R20T24	R20T18	R20T17	R19T17	baseMeanA	baseMeanB	2FoldChange	pval	padj	FuncAnn
Ciclev10025649m.g	179	137	172	1085	534	456	161.156069	679.685011	2.07640779	0.00248458	0.08599308	CBL-interacting protein kinase 25;
Ciclev10003072m.g	407	129	113	11	21	14	207.496329	15.0112142	-3.7889732	0.00250318	0.08650398	
Ciclev10012875m.g	42878	23710	19371	13702	11557	6547	27853.3061	10329.3227	-1.4311029	0.00251743	0.08686281	
Ciclev10031790m.g	228	344	335	792	1022	673	302.415001	810.982069	1.42314031	0.00254067	0.08738275	Magnesium transporter CorA-like family protein;
Ciclev10031210m.g	79	86	62	207	319	187	74.6384181	231.72254	1.63440807	0.00254401	0.08738275	cytokinin oxidase/dehydrogenase 6;
Ciclev10025175m.g	99	77	66	262	192	382	79.2117243	281.556178	1.82963692	0.00254415	0.08738275	Leucine-rich repeat protein kinase family protein;
Ciclev10029471m.g	5165	4038	3302	1607	2624	567	4089.38174	1523.84885	-1.4241629	0.00254843	0.08739644	proline-rich family protein;
Ciclev10013209m.g	1900	1861	3263	522	1085	540	2354.63456	694.236496	-1.7620041	0.00257254	0.08808883	basic helix-loop-helix (bHLH) DNA-binding family protein;
Ciclev10020608m.g	6730	7370	6965	2729	3483	1925	6969.56518	2640.2931	-1.4003706	0.00260561	0.08908577	gamma-glutamyl hydrolase 2;
Ciclev10000950m.g	157	126	120	406	486	280	132.340144	380.728839	1.5245131	0.00260976	0.08909217	MATE efflux family protein;
Ciclev10021984m.g	224	377	240	796	984	529	278.425279	748.690556	1.42707935	0.00261946	0.08928778	HSP20-like chaperones superfamily protein;
Ciclev10017369m.g	177	210	236	71	92	33	207.371233	62.9363035	-1.7202514	0.00266984	0.09083991	
Ciclev10017663m.g	1	1	1	31	31	5	0.99177012	21.2950693	4.42436986	0.00267307	0.09083991	
Ciclev10017557m.g	35	65	29	145	120	182	42.5059898	149.360218	1.81305788	0.00268132	0.09098295	Protein kinase family protein;
Ciclev10028437m.g	14	24	16	115	78	48	17.9007498	78.4156196	2.13112104	0.00270136	0.09152482	UDP-glucosyl transferase 76E2;
Ciclev10033325m.g	0	2	0	14	19	26	0.66139801	19.7178326	4.89783846	0.00271849	0.09196678	disease resistance family protein / LRR family protein;
Ciclev10006428m.g_Cic	511	391	290	44	105	267	388.768565	142.699206	-1.4459343	0.00272464	0.09203634	P-loop containing nucleoside triphosphate hydrolases superfamily protein;P-loop containing nucleoside triphosphate hydrolases superfamily protein;
Ciclev10009498m.g	6177	1711	3096	250	682	810	3557.08144	581.101421	-2.6138321	0.00273579	0.09227453	
Ciclev10011390m.g	2657	2260	1427	737	652	1040	2067.82414	813.482361	-1.3459305	0.00276916	0.09326029	
Ciclev10011232m.g	47	22	49	93	151	172	39.0546985	138.247032	1.82368048	0.00277702	0.09338508	Protein kinase superfamily protein;
Ciclev10010159m.g	610	698	874	268	385	150	727.666397	258.210103	-1.4947317	0.00278561	0.09353398	cyclic nucleotide-gated channel 14;
Ciclev10015544m.g	68	45	48	8	15	5	52.7445095	8.96114044	-2.5572667	0.0027945	0.0936928	
Ciclev10014223m.g	149	53	54	4	11	21	82.3501817	12.230955	-2.7512348	0.00282958	0.09472787	NB-ARC domain-containing disease resistance protein;
Ciclev10009223m.g	201	282	181	17	22	151	219.042631	66.7937668	-1.7134263	0.00284403	0.09507011	Concanavalin A-like lectin protein kinase family protein;
Ciclev10022948m.g	1325	2358	1847	6028	4632	3661	1840.75077	4689.20673	1.34904958	0.00285036	0.09514033	SAUR-like auxin-responsive protein family ;
Ciclev10028446m.g	53522	28573	19529	10013	12914	4402	32780.7639	8766.32431	-1.9028055	0.00287754	0.09587365	GTP binding Elongation factor Tu family protein;
Ciclev10002417m.g	24154	26610	16609	8044	14631	3734	22092.7921	8405.76888	-1.3941241	0.00288488	0.09587365	F-box family protein;
Ciclev10030084m.g	54	31	42	6	6	6	41.6956404	5.94089028	-2.8111455	0.00288512	0.09587365	AP2/B3-like transcriptional factor family protein;
Ciclev10033671m.g	162	45	85	5	17	25	94.6793173	15.823175	-2.5810102	0.00290878	0.09644911	CLAVATA3/ESR-RELATED 9;
Ciclev10001851m.g	1878	1153	1268	316	556	766	1406.54917	548.41157	-1.3588291	0.00291101	0.09644911	
Ciclev10024351m.g	0	0	0	8	33	7	0	15.1472188	Inf	0.0029211	0.09664116	
Ciclev10020640m.g	70421	50533	63110	3359	10352	44700	60672.832	20393.9134	-1.572912	0.00293904	0.09709187	GDSL-like Lipase/Acylhydrolase superfamily protein;
Ciclev10026280m.g	464	485	499	145	233	142	479.53313	169.195138	-1.5029424	0.00295274	0.09731229	F-box family protein;
Ciclev10030225m.g	7	10	5	153	33	80	7.2255097	88.6814626	3.6174613	0.00295436	0.09731229	cytochrome P450, family 76, subfamily C, polypeptide 4;
Ciclev10021105m.g	6493	3314	3356	1866	2348	795	4276.23903	1606.71653	-1.4122271	0.00297311	0.09778666	pfkB-like carbohydrate kinase family protein;
Ciclev10030153m.g	0	0	0	18	9	52	0	27.3828458	Inf	0.0030061	0.0987273	transcription factor-related;
Ciclev10011753m.g	3	3	2	38	17	33	2.62082128	29.4444926	3.48990693	0.00304619	0.09989801	receptor serine/threonine kinase, putative;
