# Supplementary material for: Transcriptome Profiling of Huanglongbing (HLB) Tolerant and Susceptible Citrus Plants Reveals the Role of Basal Resistance in HLB Tolerance
Source: Front Plant Sci. 2016 Jun 28;7:933. doi: 10.3389/fpls.2016.00933 (PMC4923198; doi:10.3389/fpls.2016.00933)
Supplement: Table S7 — DE genes related to disease response identified by MapMan. [file Table7.PDF]

Table S7. DE genes related to disease response identified by MapMan

Class	Familiy	GeneID	Description	log2(R/S)	pval
Transcript factor.ERF	RNA.regulation of transcription.AP2/EREBP, APETALA2/Ethylene-responsive element binding protein family	ciclev10005701m|pacid:20792340	weakly similar to (160) AT3G16770 | Symbols: RAP2.3, ATEBP, ERF72 | ATEBP (ETHYLENE-RESPONSIVE ELEMENT BINDING PROTEIN); DNA binding / protein binding / transcription activator/ transcription factor | chr3:5705784-5706768 FORWARDvery weakly similar to (90.9) ERF1_ORYSA Ethylene-responsive transcription factor 1 (Ethylene-responsive element-binding factor 1) (EREBP-1) (OsEREBP1) - Oryza sativa (Rice)very weakly similar to (91.3) loc_os06g09390 12006.m091636 protein ethylene-responsive element binding protein 1, putative, expressedAP2 AP2 no original description	-3.4628572	0.01074975
Transcript factor.bZIP	RNA.regulation of transcription.bZIP transcription factor family	ciclev10031441m|pacid:20802696	moderately similar to (428) AT1G68640 | Symbols: PAN | PAN (PERIANTHIA); DNA binding / transcription factor | chr1:25769739-25772303 REVERSEmoderately similar to (365) HBP1C_WHEAT Transcription factor HBP-1b(c1) (Fragment) - Triticum aestivum (Wheat)moderately similar to (377) loc_os06g15480 12006.m32041 protein transcription factor HBP-1b, putative, expressed no original description	-2.5092442	0.02122258
Transcript factor.bZIP	RNA.regulation of transcription.bZIP transcription factor family	ciclev10022056m|pacid:20810096	weakly similar to (188) AT3G14880 | Symbols: | FUNCTIONS IN: molecular_function unknown; INVOLVED IN: biological_process unknown; BEST Arabidopsis thaliana protein match is: DOG1 (DELAY OF GERMINATION 1) (TAIR:AT5G45830.1); Has 368 Blast hits to 367 proteins in 28 species: Archae - 0; Bacteria - 0; Metazoa - 2; Fungi - 0; Plants - 366; Viruses - 0; Other Eukaryotes - 0 (source: NCBI BLink). | chr3:5006565-5007689 FORWARDvery weakly similar to (97.4) loc_os05g48650 12005.m08936 protein tumor-related protein-like, putative, expressed no original description	-3.16216	0.00129172
Transcript factor.bZIP	RNA.regulation of transcription.bZIP transcription factor family	ciclev10015966m|pacid:20816343	moderately similar to (280) AT3G58120 | Symbols: ATBZIP61, BZIP61 | BZIP61; DNA binding / transcription activator/ transcription factor | chr3:21521289-21523078 REVERSEvery weakly similar to (84.0) RF2A_ORYSA Transcription factor RF2a - Oryza sativa (Rice)weakly similar to (198) loc_os01g11350 12001.m07754 protein transcription factor RF2b, putative, expressed no original description	-2.0819943	0.0017926
Transcript factor.MYB	RNA.regulation of transcription.MYB domain transcription factor family	ciclev10031944m|pacid:20804198	moderately similar to (236) AT1G69560 | Symbols: MYB105, AtMYB105 | MYB105 (myb domain protein 105); DNA binding / transcription factor | chr1:26157755-26158906 FORWARDweakly similar to (103) MYB1_MAIZE Myb-related protein Zm1 - Zea mays (Maize)moderately similar to (242) loc_os08g33800 12008.m07370 protein myb-like DNA-binding domain containing protein REB1 no original description	-3.1979697	0.01293749

Class	Familiy	GeneID	Description	log2(R/S)	pval
Transcript factor.MYB	RNA.regulation of transcription.MYB domain transcription factor family	ciclev10022303m|pacid:20808564	weakly similar to (162) AT5G52600 | Symbols: AtMYB82 | AtMYB82 (myb domain protein 82); DNA binding / transcription factor | chr5:21343197-21343968 REVERSEweakly similar to (166) MYBC_MAIZE Anthocyanin regulatory C1 protein - Zea mays (Maize)weakly similar to (166) loc_os03g29614 12003.m08231 protein anthocyanin regulatory C1 protein, putative, expressed no original description	-1.5388075	0.05173288
Transcript factor.MYB	RNA.regulation of transcription.MYB domain transcription factor family	ciclev10022991m|pacid:20810025	weakly similar to (164) AT3G23250 | Symbols: MYB15, ATY19, ATMYB15 | MYB15 (MYB DOMAIN PROTEIN 15); DNA binding / transcription factor | chr3:8309742-8310624 FORWARDweakly similar to (171) MYB4_ORYSA Myb-related protein Myb4 (OsMyb4) (Transcription factor RLTR1) - Oryza sativa (Rice)weakly similar to (171) loc_os04g43680 12004.m09327 protein myb-related protein Myb4, putative, expressed no original description	4.458537	0.02607845
Transcript factor.MYB	RNA.regulation of transcription.MYB domain transcription factor family	ciclev10006897m|pacid:20790071	weakly similar to (173) AT5G52260 | Symbols: AtMYB19 | AtMYB19 (myb domain protein 19); DNA binding / transcription factor | chr5:21220165-21221223 FORWARDweakly similar to (163) MYB3_HORVU Myb-related protein Hv33 - Hordeum vulgare (Barley)weakly similar to (194) loc_os02g42850 12002.m09320 protein transcription factor LAF1, putative, expressed no original description	-1.8773547	0.07181339
Hormone signaling.Auxins	hormone metabolism.auxin.induced-regulated-responsive-activated	ciclev10022948m|pacid:20806466	weakly similar to (110) AT3G61900 | Symbols: | auxin-responsive family protein | chr3:22925813-22926379 FORWARDAuxin_inducible no original description	1.3490496	0.09514033
Hormone signaling.Auxins	hormone metabolism.auxin.induced-regulated-responsive-activated	ciclev10030014m|pacid:20813343	weakly similar to (116) AT4G38840 | Symbols: | auxin-responsive protein, putative | chr4:18125174-18125473 REVERSEweakly similar to (106) A10A5_SOYBN Auxin-induced protein 10A5 - Glycine max (Soybean)Auxin_inducible no original description	7.770316	0.03040378
Hormone signaling.Auxins	hormone metabolism.auxin.induced-regulated-responsive-activated	ciclev10032615m|pacid:20804275	weakly similar to (166) AT3G07390 | Symbols: AIR12 | AIR12; extracellular matrix structural constituent | chr3:2365452-2366273 FORWARDweakly similar to (137) loc_os08g41290 12008.m08107 protein AIR12, putative, expressed DUF568 no original description	-1.7580322	0.0088359
Hormone signaling.Auxins	hormone metabolism.auxin.induced-regulated-responsive-activated	ciclev10022581m|pacid:20806571	weakly similar to (164) AT4G22620 | Symbols: | auxin-responsive family protein | chr4:11907631-11908113 FORWARDvery weakly similar to (83.6) loc_os02g42990 12002.m100801 protein OsSAUR11 - Auxin-responsive SAUR gene family member, expressedAuxin_inducible no original description	2.8584669	0.03311688
Hormone signaling.Brassinosteroid	hormone metabolism.brassinosteroid.synthesis-degradation.BRs.DET2	ciclev10013348m|pacid:20798626	moderately similar to (319) AT2G38050 | Symbols: DET2, DWF6, ATDET2 | DET2 (DE-ETIOLATED 2); sterol 5-alpha reductase | chr2:15921303-15922176 REVERSEweakly similar to (179) loc_os01g63260 12001.m12442 protein steroid reductase DET2, putative, expressed Steroid_dh no original description	-1.7980121	0.04732358

Class	Familiy	GeneID	Description	log2(R/S)	pval
Hormone signaling.Brassinosteroid	hormone metabolism.brassinosteroid.synthesis-degradation.sterols.HYD1	ciclev10006027m|pacid:20791879	weakly similar to (163) AT1G20050 | Symbols: HYD1 | HYD1 (HYDRA1); C-8 sterol isomerase | chr1:6949160-6950135 FORWARDweakly similar to (143) EBP_ORYSA Probable 3-beta-hydroxysteroid-Delta(8),Delta(7)-isomerase (EC 5.3.3.5) (Cholestenol Delta-isomerase) (Delta(8)-Delta(7) sterol isomerase) (D8-D7 sterol isomerase) - Oryza sativa (Rice)weakly similar to (143) loc_os01g01369 12001.m150882 protein 3-beta-hydroxysteroid-delta-isomerase, putative, expressed EBP no original description	-5.3842583	4.12E-09
Hormone signaling.Ethylene	hormone metabolism.ethylene.synthesis-degradation	ciclev10022725m|pacid:20806801	moderately similar to (233) AT5G24530 | Symbols: DMR6 | DMR6 (DOWNY MILDEW RESISTANT 6); oxidoreductase/ oxidoreductase, acting on paired donors, with incorporation or reduction of molecular oxygen, 2-oxoglutarate as one donor, and incorporation of one atom each of oxygen into both donors | chr5:8378964-8383154 FORWARDweakly similar to (139) ACCO_PEA 1-aminocyclopropane-1-carboxylate oxidase (EC 1.14.17.4) (ACC oxidase) (Ethylene-forming enzyme) (EFE) - Pisum sativum (Garden pea)moderately similar to (252) loc_os03g03034 12003.m35589 protein flavonol synthase/flavanone 3-hydroxylase, putative, expressed 2OG-FeII_Oxy PcbC no original description	-2.481379	4.78E-04
Hormone signaling.Ethylene	hormone metabolism.ethylene.synthesis-degradation	ciclev10020843m|pacid:20807380	moderately similar to (422) AT1G17020 | Symbols: SRG1, ATSRG1 | SRG1 (SENESCENCE-RELATED GENE 1); oxidoreductase, acting on diphenols and related substances as donors, oxygen as acceptor / oxidoreductase, acting on paired donors, with incorporation or reduction of molecular oxygen, 2-oxoglutarate as one donor, and inc | chr1:5820258-5821741 FORWARDweakly similar to (180) FLS_PETHY Flavonol synthase/flavanone 3-hydroxylase (EC 1.14.11.23) (EC 1.14.11.9) (FLS) - Petunia hybrida (Petunia)moderately similar to (281) loc_os01g25010 12001.m08967 protein flavonol synthase/flavanone 3-hydroxylase, putative, expressed PcbC 2OG-FeII_Oxy no original description	2.5800436	3.96E-05
Hormone signaling.Ethylene	hormone metabolism.ethylene.synthesis-degradation	ciclev10028699m|pacid:20812923	moderately similar to (374) AT3G19000 | Symbols: | oxidoreductase, 2OG-Fe(II) oxygenase family protein | chr3:6554004-6554987 REVERSEweakly similar to (160) GAO1B_WHEAT Gibberellin 20 oxidase 1-B (EC 1.14.11.-) (Gibberellin C-20 oxidase 1-B) (GA 20-oxidase 1-B) (Ta20ox1B) (TaGA20ox1-B) - Triticum aestivum (Wheat)moderately similar to (394) loc_os03g42130 12003.m09271 protein gibberellin 20 oxidase 2, putative, expressed PcbC 2OG-FeII_Oxy no original description	-99	6.26E-05

Class	Familiy	GeneID	Description	log2(R/S)	pval
Hormone signaling.Ethylene	hormone metabolism.ethylene.synthesis-degradation	ciclev10006569m|pacid:20789952	moderately similar to (344) AT1G06620 | Symbols: | 2-oxoglutarate-dependent dioxygenase, putative | chr1:2025618-2027094 FORWARDmoderately similar to (346) DV4H_CATRO Desacetoxyvindoline 4-hydroxylase (EC 1.14.11.20) - Catharanthus roseus (Rosy periwinkle) (Madagascar periwinkle)moderately similar to (275) loc_os03g48430 12003.m09851 protein 1-aminocyclopropane-1-carboxylate oxidase, putative, expressed PcbC 2OG-FeII_Oxy no original description	-2.593697	2.05E-04
Hormone signaling.Ethylene	hormone metabolism.ethylene.synthesis-degradation	ciclev10029921m|pacid:20813228	moderately similar to (216) AT3G19000 | Symbols: | oxidoreductase, 2OG-Fe(II) oxygenase family protein | chr3:6554004-6554987 REVERSEweakly similar to (137) GAOX2_ORYSA Gibberellin 20 oxidase 2 (EC 1.14.11.-) (Gibberellin C-20 oxidase 2) (GA 20-oxidase 2) (Os20ox2) (Semidwarf-1 protein) - Oryza sativa (Rice)moderately similar to (257) loc_os03g42130 12003.m09271 protein gibberellin 20 oxidase 2, putative, expressed PcbC 2OG-FeII_Oxy no original description	-99	0.00959151
Hormone signaling.Ethylene	hormone metabolism.ethylene.signal transduction	ciclev10017663m|pacid:20815464	weakly similar to (115) AT4G31980 | Symbols: | unknown protein | chr4:15464905-15469204 FORWARDvery weakly similar to (84.7) loc_os12g29650 12012.m06779 protein expressed proteinDUF247 no original description	4.42437	0.09083991
Cell wall	cell wall.precursor synthesis.UXS	ciclev10004959m|pacid:20792473	highly similar to (657) AT3G62830 | Symbols: UXS2, ATUXS2 | AUD1; UDP-glucuronate decarboxylase/ catalytic/ dTDP-glucose 4,6-dehydratase | chr3:23232539-23235353 FORWARDweakly similar to (121) GME1_ORYSA GDP-mannose 3,5-epimerase 1 (EC 5.1.3.18) (GDP-Man 3,5-epimerase 1) (OsGME-1) - Oryza sativa (Rice)highly similar to (626) loc_os05g29990 12005.m07235 protein UDP-glucuronic acid decarboxylase 1, putative, expressed WcaG Epimerase RfbB GalE PRK10084 Gmd PRK08125 PRK10217 PRK11908 PRK10675 RfbD no original description	-2.1916707	2.66E-04
Cell wall	cell wall.cellulose synthesis	ciclev10030861m|pacid:20802780	highly similar to (927) AT3G07330 | Symbols: ATCSLC06, CSLC06, ATCSLC6 | ATCSLC6 (CELLULOSE-SYNTHASE LIKE C6); cellulose synthase/ transferase, transferring glycosyl groups | chr3:2336121-2338942 REVERSEhighly similar to (733) loc_os05g43530 12005.m08480 protein CSLC7 - cellulose synthase-like family C, expressedCOG1215 bcsA no original description	-3.1265883	0.01483174
Cell wall	cell wall.cellulose synthesis	ciclev10014531m|pacid:20816884	highly similar to (955) AT3G28180 | Symbols: ATCSLC04, CSLC04, ATCSLC4, CSLC4 | ATCSLC04 (CELLULOSE-SYNTHASE LIKE C4); cellulose synthase/ transferase, transferring glycosyl groups | chr3:10506110-10509067 FORWARDhighly similar to (783) loc_os08g15420 12008.m05665 protein CSLC3 - cellulose synthase-like family C, expressedCOG1215 bcsA no original description	-1.405692	0.07588015

Class	Familiy	GeneID	Description	log2(R/S)	pval
Cell wall	cell wall.cellulose synthesis.cellulose synthase	ciclev10023570m|pacid:20807251	highly similar to (879) AT4G23990 | Symbols: ATCSLG3, CSLG3 | ATCSLG3; cellulose synthase/ transferase/ transferase, transferring glycosyl groups | chr4:12456491-12460498 FORWARDmoderately similar to (488) loc_os09g30130 12009.m060004 protein CSLE6 - cellulose synthase-like family E, expressedCellulose_synt no original description	-2.334664	0.0130799
Cell wall	cell wall.cell wall proteins.AGPs.AGP	ciclev10020328m|pacid:20806955	moderately similar to (443) AT3G60900 | Symbols: FLA10 | FLA10 | chr3:22499573-22500841 REVERSEmoderately similar to (339) loc_os04g48490 12004.m09785 protein fasciclin-like arabinogalactan protein 10 precursor, putative, expressed no original description	-1.7819489	0.0082457
Cell wall	cell wall.cell wall proteins.RGP	ciclev10020723m|pacid:20806109	highly similar to (659) AT3G08900 | Symbols: RGP3, RGP | RGP3 (REVERSIBLY GLYCOSYLATED POLYPEPTIDE 3); transferase, transferring hexosyl groups | chr3:2708347-2709714 REVERSEhighly similar to (676) UPTG_PEA Alpha-1,4-glucan-protein synthase [UDP-forming] (EC 2.4.1.112) (UDP-glucose:protein transglucosylase) (UPTG) (Reversibly glycosylated polypeptide) - Pisum sativum (Garden pea)highly similar to (682) loc_os07g41360 12007.m08372 protein alpha-1,4-glucan-protein synthase 1, putative, expressedRGP no original description	-2.3015063	9.14E-05
Cell wall	cell wall.degradation.cellulases and beta -1,4-glucanases	ciclev10019799m|pacid:20812041	highly similar to (576) AT1G02800 | Symbols: ATCEL2, CEL2 | ATCEL2; cellulase/ hydrolase, hydrolyzing O-glycosyl compounds | chr1:613386-616103 REVERSEhighly similar to (612) GUN19_ORYSA Endoglucanase 19 precursor (EC 3.2.1.4) (Endo-1,4-beta glucanase 19) - Oryza sativa (Rice)highly similar to (612) loc_os08g02220 12008.m04366 protein endoglucanase 1 precursor, putative, expressedGlyco_hydro_9 no original description	-3.519792	7.36E-05
Cell wall	cell wall.degradation.cellulases and beta -1,4-glucanases	ciclev10014994m|pacid:20817143	highly similar to (828) AT1G70710 | Symbols: ATGH9B1, CEL1 | ATGH9B1 (ARABIDOPSIS THALIANA GLYCOSYL HYDROLASE 9B1); cellulase/ hydrolase, hydrolyzing O-glycosyl compounds | chr1:26659356-26662962 REVERSEhighly similar to (733) GUN17_ORYSA Endoglucanase 17 precursor (EC 3.2.1.4) (Endo-1,4-beta glucanase 17) (OsGLU13) - Oryza sativa (Rice)highly similar to (733) loc_os06g14540 12006.m06169 protein endoglucanase 1 precursor, putative, expressedGlyco_hydro_9 no original description	-2.3650746	3.95E-04
Cell wall	cell wall.degradation.cellulases and beta -1,4-glucanases	ciclev10028301m|pacid:20813445	highly similar to (707) AT4G39010 | Symbols: AtGH9B18 | AtGH9B18 (Arabidopsis thaliana glycosyl hydrolase 9B18); catalytic/ hydrolase, hydrolyzing O-glycosyl compounds | chr4:18176162-18179102 REVERSEhighly similar to (674) GUN23_ORYSA Endoglucanase 23 precursor (EC 3.2.1.4) (Endo-1,4-beta glucanase 23) (OsGLU12) - Oryza sativa (Rice)highly similar to (674) loc_os09g36060 12009.m06556 protein endoglucanase 1 precursor, putative, expressedGlyco_hydro_9 no original description	-1.5723176	0.03537947

Class	Familiy	GeneID	Description	log2(R/S)	pval
Cell wall	cell wall.degradation.pectate lyases and polygalacturonases	ciclev10029006m|pacid:20812585	weakly similar to (176) AT1G49320 | Symbols: | BURP domain-containing protein | chr1:18246441-18247817 FORWARDweakly similar to (173) loc_os01g53240 12001.m11493 protein dehydration-induced protein RD22-like protein 2, putative, expressedBURP no original description	-3.9997144	4.02E-05
Cell wall	cell wall.modification	ciclev10016123m|pacid:20816630	highly similar to (526) AT5G13870 | Symbols: EXGT-A4 | EXGT-A4 (ENDOXYLOGLUCAN TRANSFERASE A4); hydrolase, acting on glycosyl bonds / hydrolase, hydrolyzing O-glycosyl compounds / xyloglucan:xyloglucosyl transferase | chr5:4475089-4476217 REVERSEhighly similar to (523) XTHB_PHAAN Probable xyloglucan endotransglucosylase/hydrolase protein B precursor (EC 2.4.1.207) (VaXTH2) - Phaseolus angularis (Adzuki bean) (Vigna angularis)moderately similar to (477) loc_os11g33270 12011.m07226 protein xyloglucan endotransglucosylase/hydrolase precursor, putative, expressedGH16_XET Glyco_hydro_16 Glyco_hydrolase_16 GH16_GPI_glucanosyltransferase GH16_lichenase no original description	-1.9350705	0.00201314
Cell wall	cell wall.modification	ciclev10012518m|pacid:20796457	moderately similar to (424) AT2G39700 | Symbols: ATEXPA4, ATEXP4, ATHEXP ALPHA 1.6 | ATEXPA4 (ARABIDOPSIS THALIANA EXPANSIN A4) | chr2:16544246-16545434 REVERSEmoderately similar to (399) EXPA7_ORYSA Expansin-A7 precursor (OsEXPA7) (Alpha-expansin-7) (OsEXP7) (OsaEXPa1.26) - Oryza sativa (Rice)moderately similar to (399) loc_os03g60720 12003.m10961 protein alpha-expansin 6 precursor, putative, expressedPollen_allerg_1 DPBB_1 no original description	-1.641307	0.01587655
Cell wall	cell wall.modification	ciclev10012611m|pacid:20797823	moderately similar to (353) AT3G29030 | Symbols: ATEXPA5, ATEXP5, ATHEXP ALPHA 1.4, EXP5, EXPA5 | EXPA5 (EXPANSIN A5) | chr3:11011538-11013068 REVERSEmoderately similar to (305) EXPA4_ORYSA Expansin-A4 precursor (OsEXPA4) (Alpha-expansin-4) (OsEXP4) (OsaEXPa1.22) - Oryza sativa (Rice)moderately similar to (305) loc_os05g39990 12005.m08176 protein alpha-expansin 1 precursor, putative, expressedPollen_allerg_1 DPBB_1 no original description	-3.2794926	7.66E-08
Cell wall	cell wall.modification	ciclev10013804m|pacid:20798473	moderately similar to (373) AT2G40610 | Symbols: ATEXPA8, EXP8, ATEXP8, ATHEXP ALPHA 1.11 | ATEXPA8 (ARABIDOPSIS THALIANA EXPANSIN A8) | chr2:16949121-16950472 REVERSEmoderately similar to (372) EXPA2_ORYSA Expansin-A2 precursor (OsEXPA2) (Alpha-expansin-2) (OsEXP2) (OsaEXPa1.23) (RiExB) (RiExC) - Oryza sativa (Rice)moderately similar to (372) loc_os01g60770 12001.m12205 protein alpha-expansin 10 precursor, putative, expressedPollen_allerg_1 DPBB_1 no original description	-2.9172523	0.00110636

Class	Familiy	GeneID	Description	log2(R/S)	pval
Cell wall	cell wall.pectinesterases.PME	ciclev10007806m|pacid:20795598	highly similar to (678) AT3G14310 | Symbols: ATPME3 | ATPME3; pectinesterase | chr3:4772214-4775095 REVERSEhighly similar to (703) PME3_CITSI Pectinesterase-3 precursor (EC 3.1.1.11) (Pectin methylesterase 3) (PE 3) - Citrus sinensis (Sweet orange)moderately similar to (469) loc_os01g21034 12001.m42984 protein pectinesterase-2 precursor, putative, expressedPectinesterase PemB PMEI PRK10531 no original description	-1.4768085	0.06548068
Cell wall	cell wall.pectinesterases.PME	ciclev10013004m|pacid:20798479	no original description	-1.9097327	0.01631435
Cell wall	cell wall.pectinesterases.PME	ciclev10007993m|pacid:20792857	highly similar to (697) AT4G33220 | Symbols: PME44, ATPME44 | enzyme inhibitor/ pectinesterase | chr4:16022506-16026130 FORWARDhighly similar to (744) PME_PRUPE Pectinesterase PPE8B precursor (EC 3.1.1.11) (Pectin methylesterase) (PE) - Prunus persica (Peach)highly similar to (526) loc_os08g34900 12008.m07479 protein pectinesterase PPE8B precursor, putative, expressedPectinesterase PemB no original description	1.5373335	0.04732358
Beta glucanase	misc.beta 1,3 glucan hydrolases	ciclev10013846m|pacid:20798263	weakly similar to (107) AT1G79480 | Symbols: | LOCATED IN: endomembrane system; CONTAINS InterPro DOMAIN/s: X8 (InterPro:IPR012946); BEST Arabidopsis thaliana protein match is: glycosyl hydrolase family protein 17 (TAIR:AT5G67460.1); Has 4247 Blast hits to 3762 proteins in 375 species: Archae - 10; Bacteria - 432; Metazoa - 1406; Fungi - 619; Plants - 1140; Viruses - 87; Other Eukaryotes - 553 (source: NCBI BLink). | chr1:29897905-29899267 REVERSEvery weakly similar to (80.1) E13B_WHEAT Glucan endo-1,3-beta-glucosidase precursor (EC 3.2.1.39) ((1->3)-beta-glucan endohydrolase) ((1->3)-beta-glucanase) (Beta-1,3-endoglucanase) - Triticum aestivum (Wheat)weakly similar to (102) loc_os06g45450 12006.m09073 protein glucan endo-1,3-beta-glucosidase precursor, putativeX8 no original description	-1.6153891	0.05826356
Beta glucanase	misc.beta 1,3 glucan hydrolases	ciclev10022230m|pacid:20811793	weakly similar to (132) AT1G18650 | Symbols: PDCB3 | PDCB3 (PLASMODESMATA CALLOSE-BINDING PROTEIN 3); callose binding / polysaccharide binding | chr1:6419036-6420413 REVERSEvery weakly similar to (90.5) E13B_WHEAT Glucan endo-1,3-beta-glucosidase precursor (EC 3.2.1.39) ((1->3)-beta-glucan endohydrolase) ((1->3)-beta-glucanase) (Beta-1,3-endoglucanase) - Triticum aestivum (Wheat)weakly similar to (107) loc_os03g54910 12003.m10406 protein glucan endo-1,3-beta-glucosidase 3 precursor, putative, expressedX8 no original description	-2.0758953	9.84E-04

Class	Familiy	GeneID	Description	log2(R/S)	pval
Beta glucanase	misc.beta 1,3 glucan hydrolases.glucan endo-1,3-beta-glucosidase	ciclev10028241m|pacid:20814669	moderately similar to (482) AT5G24318 | Symbols: | catalytic/ cation binding / hydrolase, hydrolyzing O-glycosyl compounds | chr5:8282390-8283956 REVERSEmoderately similar to (466) E13B_WHEAT Glucan endo-1,3-beta-glucosidase precursor (EC 3.2.1.39) ((1->3)-beta-glucan endohydrolase) ((1->3)-beta-glucanase) (Beta-1,3-endoglucanase) - Triticum aestivum (Wheat)moderately similar to (477) loc_os11g47820 12011.m08580 protein glucan endo-1,3-beta-glucosidase precursor, putative, expressedGlyco_hydro_17 X8 no original description	-1.6297542	0.06428538
Beta glucanase	misc.beta 1,3 glucan hydrolases.glucan endo-1,3-beta-glucosidase	ciclev10015086m|pacid:20816294	highly similar to (736) AT2G05790 | Symbols: | glycosyl hydrolase family 17 protein | chr2:2199450-2201293 FORWARDmoderately similar to (291) E13B_WHEAT Glucan endo-1,3-beta-glucosidase precursor (EC 3.2.1.39) ((1->3)-beta-glucan endohydrolase) ((1->3)-beta-glucanase) (Beta-1,3-endoglucanase) - Triticum aestivum (Wheat)highly similar to (540) loc_os02g53200 12002.m10349 protein glucan endo-1,3-beta-glucosidase 7 precursor, putative, expressedGlyco_hydro_17 X8 no original description	-2.163776	0.00670767
Proteolysis	protein.degradation	ciclev10013211m|pacid:20798512	weakly similar to (105) AT1G14570 | Symbols: | UBX domain-containing protein | chr1:4983770-4987192 FORWARDweakly similar to (107) loc_os04g57520 12004.m10637 protein UBX domain-containing protein 7, putative, expressed no original description	8.303	8.74E-08
Proteolysis	protein.degradation	ciclev10023127m|pacid:20806758	very weakly similar to (96.7) AT1G78660 | Symbols: ATGGH1 | gamma-glutamyl hydrolase, putative / gamma-Glu-X carboxypeptidase, putative / conjugase, putative | chr1:29585901-29588117 FORWARDvery weakly similar to (92.8) loc_os05g44130 12005.m27626 protein gamma-glutamyl hydrolase precursor, putative, expressed GATase1_Glutamyl_Hydrolase no original description	4.6642375	1.35E-07
Proteolysis	protein.degradation.cysteine protease	ciclev10031514m|pacid:20802509	very weakly similar to (80.5) loc_os01g25370 12001.m43010 protein SUMO protease, putative, expressed Peptidase_C48 no original description	7.0172777	2.48E-11
Proteolysis	protein.degradation.cysteine protease	ciclev10020105m|pacid:20808956	very weakly similar to (80.9) AT4G15880 | Symbols: ESD4 | ESD4 (EARLY IN SHORT DAYS 4); SUMO-specific protease/ cysteine-type peptidase | chr4:9012769-9015797 FORWARDvery weakly similar to (82.4) loc_os01g25370 12001.m43010 protein SUMO protease, putative, expressed Peptidase_C48 no original description	5.4511876	1.52E-14
Proteolysis	protein.degradation.aspartate protease	ciclev10007181m|pacid:20790305	moderately similar to (366) AT1G03220 | Symbols: | extracellular dermal glycoprotein, putative / EDGP, putative | chr1:787143-788444 FORWARDmoderately similar to (206) 7SB1_SOYBN Basic 7S globulin precursor (Bg) (SBg7S) [Contains: Basic 7S globulin high kDa subunit; Basic 7S globulin low kDa subunit] - Glycine max (Soybean)moderately similar to (333) loc_os05g33430 12005.m07575 protein basic 7S globulin precursor, putative, expressed no original description	-4.6260986	5.27E-11

Class	Familiy	GeneID	Description	log2(R/S)	pval
Proteolysis	protein.degradation.serine protease	ciclev10020491m|pacid:20808161	moderately similar to (242) AT3G27925 | Symbols: DEGP1, Deg1 | DEGP1 (DegP protease 1); serine-type endopeptidase/ serine-type peptidase | chr3:10366659-10368864 REVERSEmoderately similar to (230) loc_os05g49380 12005.m083815 protein protease Do-like 1, chloroplast precursor, putative, expressed DegQ PRK10942 PRK10139 PRK10898 ilvH ACT_AHAS ilvH no original description	9.10128	1.26E-31
Proteolysis	protein.degradation.AAA type	ciclev10006428m|pacid:20790866	moderately similar to (468) AT1G04730 | Symbols: | AAA-type ATPase family protein | chr1:1325385-1331086 REVERSEmoderately similar to (380) loc_os03g15810 12003.m07018 protein ATP binding protein, putative, expressed PRK04195 no original description	-1.4459343	0.09203634
Proteolysis	protein.degradation.AAA type	ciclev10006732m|pacid:20791220	moderately similar to (388) AT1G04730 | Symbols: | AAA-type ATPase family protein | chr1:1325385-1331086 REVERSEmoderately similar to (331) loc_os03g15810 12003.m07018 protein ATP binding protein, putative, expressed no original description	-1.4459343	0.09203634
Proteolysis	protein.degradation.AAA type	ciclev10028347m|pacid:20814770	moderately similar to (419) AT3G50930 | Symbols: BCS1 | BCS1 (CYTOCHROME BC1 SYNTHESIS); ATP binding / ATPase/ nucleoside-triphosphatase/ nucleotide binding | chr3:18929817-18931547 FORWARDmoderately similar to (363) loc_os01g42030 12001.m10470 protein mitochondrial chaperone BCS1, putative, expressed AAA no original description	2.7775612	0.00336317
Proteolysis	protein.degradation.ubiquitin	ciclev10018876m|pacid:20811796	weakly similar to (196) AT3G47890 | Symbols: | ubiquitin thiolesterase/ zinc ion binding | chr3:17667172-17673530 REVERSEweakly similar to (147) loc_os03g06950 12003.m06212 protein cysteine-type endopeptidase/ nucleic acid binding protein, putative, expressedDUF629 no original description	3.9994984	0.0012166
Proteolysis	protein.degradation.ubiquitin.E2	ciclev10016889m|pacid:20818646	moderately similar to (296) AT3G20060 | Symbols: UBC19 | UBC19 (ubiquitin-conjugating enzyme19); ubiquitin-protein ligase | chr3:7002927-7003727 REVERSEweakly similar to (129) UBC2_MEDSA Ubiquitin-conjugating enzyme E2-17 kDa (EC 6.3.2.19) (Ubiquitin-protein ligase) (Ubiquitin carrier protein) - Medicago sativa (Alfalfa)moderately similar to (248) loc_os01g16650 12001.m08261 protein ubiquitin-conjugating enzyme X, putative, expressed UQ_con UBCc COG5078 UBCc no original description	-1.709893	0.06513786
Proteolysis	protein.degradation.ubiquitin.E3.RING	ciclev10023776m|pacid:20809503	weakly similar to (140) AT3G14250 | Symbols: | protein binding / zinc ion binding | chr3:4745963-4746958 REVERSEweakly similar to (130) loc_os08g35060 12008.m07495 protein ubiquitin-conjugating enzyme 7-interacting protein 4, putative, expressed no original description	-2.7419868	0.0018658
Proteolysis	protein.degradation.ubiquitin.E3.RING	ciclev10009160m|pacid:20795187	moderately similar to (233) AT3G53690 | Symbols: | zinc finger (C3HC4-type RING finger) family protein | chr3:19898997-19900044 REVERSEmoderately similar to (227) loc_os09g25190 12009.m05671 protein ubiquitin-protein ligase/ zinc ion binding protein, putative, expressed no original description	1.7473625	0.07092245

Class	Familiy	GeneID	Description	log2(R/S)	pval
Proteolysis	protein.degradation.ubiquitin.E3.RING	ciclev10021802m|pacid:20810059	weakly similar to (109) AT5G47610 | Symbols: | zinc finger (C3HC4-type RING finger) family protein | chr5:19301399-19301899 REVERSE no original description	-2.173604	3.81E-04
Proteolysis	protein.degradation.ubiquitin.E3.RING	ciclev10014457m|pacid:20815123	moderately similar to (484) AT2G38970 | Symbols: | zinc finger (C3HC4-type RING finger) family protein | chr2:16274135-16276651 FORWARDmoderately similar to (468) loc_os02g56280 12002.m10651 protein protein binding protein, putative, expressedvWA_C3HC4_type vWA_subgroup vWA_interalpha_trypsin_inhibitor vWFA no original description	-3.7776165	0.00281091
Proteolysis	protein.degradation.ubiquitin.E3.SCF.FBOX	ciclev10011675m|pacid:20798975	weakly similar to (150) AT1G69630 | Symbols: | F-box family protein | chr1:26191640-26193174 REVERSEvery weakly similar to (90.5) loc_os11g09670 12011.m079961 protein F-box domain containing protein, expressed no original description	-9.913525	7.39E-21
Proteolysis	protein.degradation.ubiquitin.E3.SCF.FBOX	ciclev10019274m|pacid:20809535	highly similar to (514) AT3G54650 | Symbols: FBL17 | FBL17; ubiquitin-protein ligase | chr3:20226004-20228882 REVERSEmoderately similar to (372) loc_os12g40860 12012.m07869 protein Leucine Rich Repeat family protein, expressed no original description	-2.5106866	3.07E-04
Proteolysis	protein.degradation.ubiquitin.E3.SCF.FBOX	ciclev10012029m|pacid:20799224	weakly similar to (175) AT2G36090 | Symbols: | F-box family protein | chr2:15158631-15159584 FORWARDweakly similar to (103) loc_os02g33240 12002.m08417 protein F-box domain containing protein, expressed no original description	3.4371197	2.93E-05
Proteolysis	protein.degradation.ubiquitin.E3.SCF.FBOX	ciclev10013437m|pacid:20799041	very weakly similar to (95.9) AT4G10400 | Symbols: | F-box family protein | chr4:6446335-6447715 REVERSE no original description	-8.522334	6.60E-13
Proteolysis	protein.degradation.ubiquitin.E3.SCF.FBOX	ciclev10012020m|pacid:20798563	weakly similar to (125) AT4G12560 | Symbols: | F-box family protein | chr4:7441815-7443157 FORWARD no original description	1.5049748	0.06191203
Proteolysis	protein.degradation.ubiquitin.E3.SCF.FBOX	ciclev10002417m|pacid:20788238	weakly similar to (161) AT4G24210 | Symbols: SLY1 | SLY1 (SLEEPY1) | chr4:12563658-12564113 FORWARDweakly similar to (104) GID2_ORYSA F-box protein GID2 (Gibberellin-insensitive dwarf protein 2) (Protein GIBBERELLIN INSENSITIVE DWARF2) - Oryza sativa (Rice) no original description	-1.394124	0.09587365
Proteolysis	protein.degradation.ubiquitin.E3.SCF.FBOX	ciclev10011664m|pacid:20796899	weakly similar to (140) AT3G03030 | Symbols: | F-box family protein | chr3:682252-683850 FORWARDvery weakly similar to (89.4) loc_os04g13160 12004.m06556 protein ribosomal RNA apurinic site specific lyase, putative, expressed no original description	-3.6731353	0.00309516
Proteolysis	protein.degradation.ubiquitin.E3.SCF.FBOX	ciclev10031775m|pacid:20804136	moderately similar to (262) AT1G31350 | Symbols: | F-box family protein | chr1:11221519-11222706 REVERSEmoderately similar to (237) loc_os06g49750 12006.m09499 protein expressed protein no original description	8.449144	7.52E-23
PR-proteins	stress.biotic.PR-proteins	ciclev10018572m|pacid:20808083	highly similar to (578) AT5G36930 | Symbols: | disease resistance protein (TIR-NBS-LRR class), putative | chr5:14567771-14571916 REVERSEweakly similar to (108) loc_os01g15580 12001.m08156 protein disease resistance protein RGA2, putative, expressed TIR TIR NB-ARC no original description	2.2085023	2.22E-02

Class	Familiy	GeneID	Description	log2(R/S)	pval
PR-proteins	stress.biotic.PR-proteins	ciclev10013959m|pacid:20799318	moderately similar to (246) AT4G12010 | Symbols: | disease resistance protein (TIR-NBS-LRR class), putative | chr4:7197325-7201393 REVERSE no original description	-4.5423503	0.06574765
PR-proteins	stress.biotic.PR-proteins	ciclev10018835m|pacid:20810296	weakly similar to (153) AT5G36930 | Symbols: | disease resistance protein (TIR-NBS-LRR class), putative | chr5:14567771-14571916 REVERSE NB-ARC no original description	3.5734	1.94E-06
PR-proteins	stress.biotic.PR-proteins	ciclev10023985m|pacid:20806170	highly similar to (666) AT5G17680 | Symbols: | disease resistance protein (TIR-NBS-LRR class), putative | chr5:5822999-5827153 FORWARDweakly similar to (129) loc_os01g15580 12001.m08156 protein disease resistance protein RGA2, putative, expressed NB-ARC TIR TIR no original description	-99	1.52E-10
PR-proteins	stress.biotic.PR-proteins	ciclev10023913m|pacid:20808671	highly similar to (530) AT5G17680 | Symbols: | disease resistance protein (TIR-NBS-LRR class), putative | chr5:5822999-5827153 FORWARDvery weakly similar to (92.4) loc_os12g29280 12012.m06742 protein disease resistance protein RGA3, putative, expressed TIR NB-ARC TIR no original description	10.559226	9.55E-27
PR-proteins	stress.biotic.PR-proteins	ciclev10018578m|pacid:20808778	highly similar to (514) AT4G12010 | Symbols: | disease resistance protein (TIR-NBS-LRR class), putative | chr4:7197325-7201393 REVERSEvery weakly similar to (96.7) loc_os02g09790 12002.m06276 protein disease resistance protein RPM1, putative, expressed NB-ARC TIR TIR no original description	6.240809	6.45E-04
PR-proteins	stress.biotic.PR-proteins.proteinase inhibitors.trypsin inhibitor	ciclev10022156m|pacid:20808320	weakly similar to (123) AT1G17860 | Symbols: | trypsin and protease inhibitor family protein / Kunitz family protein | chr1:6149343-6149933 FORWARDSTI STI Kunitz_legume no original description	99	3.01E-02
PR-proteins	stress.biotic.PR-proteins.proteinase inhibitors.trypsin inhibitor	ciclev10022129m|pacid:20806463	weakly similar to (114) AT1G17860 | Symbols: | trypsin and protease inhibitor family protein / Kunitz family protein | chr1:6149343-6149933 FORWARDSTI Kunitz_legume STI no original description	3.175379	2.10E-02
Secondary metabolites	secondary	ciclev10029878m|pacid:20813099	highly similar to (664) AT4G34350 | Symbols: CLB6, ISPH, HDR | HDR	5.192735	2.95E-06
Secondary metabolites	secondary	ciclev10008936m|pacid:20793415	very weakly similar to (93.2) AT4G36810 | Symbols: GGPS1 | GGPS1	-2.2101	2.51E-02
Secondary metabolites	secondary	ciclev10033766m|pacid:20804632	highly similar to (608) AT1G78950 | Symbols: | beta-amyrin synthase,	2.190874	5.24E-04
Secondary metabolites	secondary	ciclev10031967m|pacid:20803490	moderately similar to (487) AT1G78955 | Symbols: CAMS1 | CAMS1	2.190874	5.24E-04
Secondary metabolites	secondary	ciclev10033377m|pacid:20803195	nearly identical (1014) AT1G78960 | Symbols: ATLUP2 | ATLUP2; beta-	2.190874	5.24E-04
Secondary metabolites	secondary	ciclev10033930m|pacid:20804789	highly similar to (938) AT1G78960 | Symbols: ATLUP2 | ATLUP2; beta-	2.190874	5.24E-04
Secondary metabolites	secondary	ciclev10028364m|pacid:20812611	highly similar to (504) AT1G78990 | Symbols: | transferase family	-4.3437304	3.20E-04
Secondary metabolites	secondary	ciclev10031464m|pacid:20803210	highly similar to (634) AT2G39980 | Symbols: | transferase family	-1.8452783	7.50E-03
Secondary metabolites	secondary	ciclev10011647m|pacid:20797445	highly similar to (640) AT5G01210 | Symbols: | transferase family	-2.0015702	0.00172957
Secondary metabolites	secondary	ciclev10015724m|pacid:20818317	weakly similar to (176) AT4G35150 | Symbols: | O-methyltransferase	6.6657166	4.17E-14
Secondary metabolites	secondary	ciclev10027290m|pacid:20800186	moderately similar to (246) AT3G26040 | Symbols: | transferase	99	0.03921814
Secondary metabolites	secondary	ciclev10011796m|pacid:20797859	moderately similar to (265) AT1G24430 | Symbols: | transferase/	4.2257047	0.01074975
Secondary metabolites	secondary	ciclev10028504m|pacid:20812352	moderately similar to (334) AT1G24430 | Symbols: | transferase/	-1.7737813	4.09E-02
Secondary metabolites	secondary metabolism.N	ciclev10016019m|pacid:20816093	weakly similar to (101) AT3G51420 | Symbols: SSL4, ATSSL4 | SSL4	-3.262266	9.56E-09
Secondary metabolites	secondary metabolism.N	ciclev10025811m|pacid:20799364	moderately similar to (400) AT3G51430 | Symbols: YLS2 | YLS2;	-2.1636674	0.05435657

Class	Familiy	GeneID	Description	log2(R/S)	pval
Secondary metabolites	secondary	ciclev10019871m|pacid:20807404	moderately similar to (206) AT5G24530 | Symbols: DMR6 | DMR6	2.2545683	8.63E-04
Secondary metabolites	secondary	ciclev10003684m|pacid:20786904	moderately similar to (281) AT2G33590 | Symbols: | cinnamoyl-CoA	1.4737439	0.07012007
Secondary metabolites	secondary	ciclev10013926m|pacid:20814925	very weakly similar to (99.4) AT4G39230 | Symbols: | isoflavone	-1.7041789	0.08274179
Secondary metabolites	secondary metabolism.flavonoids.isoflavones.isoflavone reductase	ciclev10012523m|pacid:20796686	moderately similar to (305) AT4G39230 | Symbols: | isoflavone reductase, putative | chr4:18266024-18267604 REVERSEmoderately similar to (284) IFRH_SOLTU Isoflavone reductase homolog (EC 1.3.1.-) (CP100) - Solanum tuberosum (Potato)moderately similar to (284) loc_os06g27770 12006.m07322 protein isoflavone reductase, putative, expressedNmrA no original description	-6.7739263	1.40E-07
Signalling	signalling.receptor kinases.leucine rich repeat III	ciclev10021063m|pacid:20806954	weakly similar to (113) AT5G58300 | Symbols: | leucine-rich repeat transmembrane protein kinase, putative | chr5:23572821-23574871 FORWARDweakly similar to (117) loc_os03g50450 12003.m10037 protein atypical receptor-like kinase MARK, putative, expressed no original description	-5.0296745	7.05E-07
Signalling	signalling.receptor kinases.leucine rich repeat X	ciclev10027614m|pacid:20802184	very weakly similar to (84.0) AT5G07280 | Symbols: EMS1, EXS | EMS1 (EXCESS MICROSPOROCYTES1); kinase/ transmembrane receptor protein kinase | chr5:2285088-2288666 FORWARD no original description	-2.2903037	0.01017689
Signalling	signalling.receptor kinases.leucine rich repeat XI	ciclev10023311m|pacid:20809114	highly similar to (506) AT3G47570 | Symbols: | leucine-rich repeat transmembrane protein kinase, putative | chr3:17527611-17530748 FORWARDmoderately similar to (308) RPK1_IPONI Receptor-like protein kinase precursor (EC 2.7.11.1) - Ipomoea nil (Japanese morning glory) (Pharbitis nil)highly similar to (525) loc_os11g46980 12011.m08478 protein receptor-like protein kinase precursor, putative Pkinase_Tyr TyrKc Pkinase S_TKc S_TKc PTKc PTKc_Fes_like PTKc_Jak_rpt2 PTKc_Srm_Brk PTKc_Src_like PTKc_Trk PTKc_Csk_like PTKc_Fer PTKc_Axl_like PTKc_EGFR_like PTKc_Fes PTKc_Chk PTKc_EphR PTKc_Ror PTKc_TrkA PTKc_Syk_like PTKc_Itk PTKc_Frk_like PTKc_Jak2_Jak3_rpt2 SPS1 PTKc_Src PTKc_Abl PTKc_TrkC PTKc_Csk PTKc_TrkB PTKc_Tec_like PTKc_EphR_A2 PTKc_Tie1 PTKc_Tyro3 PTKc_Yes PTKc_c-ros PTKc_InsR_like PTKc_Met_Ron PTKc_Fyn_Yrk PTKc_Zap-70 PTKc_ALK_LTK no original description	99	0.00250858

Class	Familiy	GeneID	Description	log2(R/S)	pval
Signalling	signalling.receptor kinases.leucine rich repeat XI	ciclev10030665m|pacid:20805277	highly similar to (530) AT4G08850 | Symbols: | kinase | chr4:5637467-5640496 REVERSEmoderately similar to (376) RPK1_IPONI Receptor-like protein kinase precursor (EC 2.7.11.1) - Ipomoea nil (Japanese morning glory) (Pharbitis nil)highly similar to (553) loc_os10g02970 12010.m03714 protein receptor-like protein kinase precursor, putative S_TKc S_TKc Pkinase Pkinase_Tyr TyrKc PTKc PTKc_EphR SPS1 PTKc_Tec_like PTKc_Csk_like PTKc_Srm_Brk PTKc_Jak_rpt2 PTKc_EphR_B PTKc_EGFR_like PTKc_Abl PTKc_Syk_like PTKc_Src_like PTKc_Btk_Bmx PTKc_Trk PTKc_Frk_like PTKc_EphR_A2 PTKc_Ack_like PTKc_Tec_Rlk PTK_CCK4 PTKc_Fes_like PTKc_Itk PTKc_Lck_Blk PTKc_EphR_A PTKc_Jak2_Jak3_rpt2 PTKc_Lyn PTKc_Ror PTKc_InsR_like PTKc_Src PTKc_TrkA PTKc_Fes PTKc_Fyn_Yrk PTKc_Hck PTKc_DDR PTKc_Chk PTKc_c-ros PTKc_FAK PTKc_EGFR PTKc_Fer PTKc_FGFR PTKc_Csk PTKc_RET PTKc_Yes PTKc_TrkC PTKc_Musk PTKc_ALK_LTK no original description	-2.8080661	1.30E-04
Signalling	signalling.receptor kinases.leucine rich repeat XI	ciclev10024083m|pacid:20811378	highly similar to (580) AT1G74190 | Symbols: AtRLP15 | AtRLP15 (Receptor Like Protein 15); protein binding | chr1:27902590-27906158 REVERSEmoderately similar to (217) RPK1_IPONI Receptor-like protein kinase precursor (EC 2.7.11.1) - Ipomoea nil (Japanese morning glory) (Pharbitis nil)moderately similar to (352) loc_os04g55420 12004.m10434 protein protein binding protein, putative, expressed no original description	6.233848	0.0015379
Signalling	signalling.receptor kinases.leucine rich repeat XI	ciclev10013524m|pacid:20798530	moderately similar to (213) AT3G47570 | Symbols: | leucine-rich repeat transmembrane protein kinase, putative | chr3:17527611-17530748 FORWARDvery weakly similar to (96.7) PSKR_DAUCA Phytosulfokine receptor precursor (EC 2.7.11.1) (Phytosulfokine LRR receptor kinase) - Daucus carota (Carrot)moderately similar to (248) loc_os02g12400 12002.m06488 protein receptor-like protein kinase precursor, putative, expressed Pkinase_Tyr PTKc TyrKc Pkinase no original description	4.4313126	3.82E-04
Signalling	signalling.receptor kinases.leucine rich repeat XI	ciclev10027783m|pacid:20814695	highly similar to (548) AT1G35710 | Symbols: | leucine-rich repeat transmembrane protein kinase, putative | chr1:13220940-13224386 FORWARDmoderately similar to (352) RPK1_IPONI Receptor-like protein kinase precursor (EC 2.7.11.1) - Ipomoea nil (Japanese morning glory) (Pharbitis nil)moderately similar to (489) loc_os10g02970 12010.m03714 protein receptor-like protein kinase precursor, putative no original description	-6.0951257	0.03430915
Signalling	signalling.receptor kinases.leucine rich repeat XI	ciclev10031263m|pacid:20803452	moderately similar to (453) AT1G68780 | Symbols: | leucine-rich repeat family protein | chr1:25831881-25833335 REVERSEweakly similar to (106) RPK1_IPONI Receptor-like protein kinase precursor (EC 2.7.11.1) - Ipomoea nil (Japanese morning glory) (Pharbitis nil)moderately similar to (423) loc_os01g02060 12001.m06848 protein too many mouths protein precursor, putative, expressed no original description	-3.5004525	9.74E-05

Class	Familiy	GeneID	Description	log2(R/S)	pval
Signalling	signalling.receptor kinases.leucine rich repeat XI	ciclev10033878m|pacid:20804469	moderately similar to (229) AT3G47570 | Symbols: | leucine-rich repeat transmembrane protein kinase, putative | chr3:17527611-17530748 FORWARDweakly similar to (180) RPK1_IPONI Receptor-like protein kinase precursor (EC 2.7.11.1) - Ipomoea nil (Japanese morning glory) (Pharbitis nil)moderately similar to (265) loc_os11g07260 12011.m04922 protein leucine-rich repeat receptor protein kinase EXS precursor, putative, expressed no original description	11.219077	0.02004569
Signalling	signalling.receptor kinases.leucine rich repeat XI	ciclev10033583m|pacid:20803519	moderately similar to (350) AT4G08850 | Symbols: | kinase | chr4:5637467-5640496 REVERSEmoderately similar to (254) RPK1_IPONI Receptor-like protein kinase precursor (EC 2.7.11.1) - Ipomoea nil (Japanese morning glory) (Pharbitis nil)moderately similar to (376) loc_os10g02970 12010.m03714 protein receptor-like protein kinase precursor, putative S_TKc S_TKc Pkinase Pkinase_Tyr TyrKc PTKc PTKc_EphR PTKc_Csk_like PTKc_EphR_B SPS1 PTKc_Tec_like PTKc_Src_like PTKc_Srm_Brk PTKc_Fes_like PTKc_Trk PTKc_Jak_rpt2 PTKc_Abl PTKc_EGFR_like PTKc_Syk_like PTKc_EphR_A2 no original description	-2.6439977	0.02538106
Signalling	signalling.receptor kinases.leucine rich repeat XI	ciclev10015508m|pacid:20817114	highly similar to (527) AT3G20820 | Symbols: | leucine-rich repeat family protein | chr3:7280930-7282027 FORWARDweakly similar to (144) PGIP1_PHAVU Polygalacturonase inhibitor 1 precursor (Polygalacturonase-inhibiting protein) (PGIP-1) - Phaseolus vulgaris (Kidney bean) (French bean)moderately similar to (383) loc_os08g39550 12008.m07936 protein polygalacturonase inhibitor 2 precursor, putative, expressed no original description	-1.8671759	0.07857219
Signalling	signalling.receptor kinases.leucine rich repeat XI	ciclev10030632m|pacid:20804646	highly similar to (562) AT4G08850 | Symbols: | kinase | chr4:5637467-5640496 REVERSEmoderately similar to (409) RPK1_IPONI Receptor-like protein kinase precursor (EC 2.7.11.1) - Ipomoea nil (Japanese morning glory) (Pharbitis nil)highly similar to (578) loc_os10g02970 12010.m03714 protein receptor-like protein kinase precursor, putative S_TKc S_TKc Pkinase Pkinase_Tyr TyrKc PTKc PTKc_EphR SPS1 PTKc_EGFR_like PTKc_Csk_like PTKc_EphR_B PTKc_Tec_like PTKc_Frk_like PTKc_Jak_rpt2 PTKc_Src_like PTKc_Ack_like PTKc_Fes_like PTKc_Srm_Brk PTKc_Trk PTKc_Abl PTKc_Syk_like PTKc_Lck_Blk PTKc_EphR_A2 PTKc_Btk_Bmx PTKc_Tec_Rlk PTKc_Itk PTKc_EphR_A PTKc_Ror PTKc_Fyn_Yrk PTKc_Jak2_Jak3_rpt2 PTKc_Fes PTKc_Lyn PTKc_Chk PTK_CCK4 PTKc_Src PTKc_TrkA PTKc_EGFR PTKc_Hck PTKc_InsR_like PTKc_c-ros PTKc_DDR PTKc_Csk PTKc_RET PTKc_ALK_LTK PTKc_HER2 PTKc_HER4 PTK_HER3 PTKc_TrkC PTKc_Musk PTKc_FAK PTKc_Yes no original description	1.8739352	0.00467704

Class	Familiy	GeneID	Description	log2(R/S)	pval
Signalling	signalling.receptor kinases.leucine rich repeat XI	ciclev10013652m|pacid:20797532	highly similar to (734) AT3G47570 | Symbols: | leucine-rich repeat transmembrane protein kinase, putative | chr3:17527611-17530748 FORWARDmoderately similar to (347) RPK1_IPONI Receptor-like protein kinase precursor (EC 2.7.11.1) - Ipomoea nil (Japanese morning glory) (Pharbitis nil)highly similar to (699) loc_os12g42520 12012.m08031 protein receptor-like protein kinase precursor, putative Pkinase_Tyr PTKc TyrKc S_TKc Pkinase S_TKc PTKc_Jak_rpt2 PTKc_Csk_like PTKc_Src_like PTKc_Srm_Brk PTKc_Trk PTKc_EphR PTKc_c-ros PTKc_Syk_like PTKc_Abl PTKc_Fes_like PTKc_EGFR_like PTKc_Frk_like PTKc_EphR_A2 PTKc_EphR_B PTKc_Itk PTKc_Tec_like PTKc_InsR_like PTKc_Fer PTKc_Jak1_rpt2 PTKc_Src PTKc_EphR_A PTKc_Fyn_Yrk PTKc_Chk PTKc_Ack_like PTKc_Jak2_Jak3_rpt2 PTKc_Ror PTKc_FAK SPS1 PTKc_Yes PTKc_TrkA PTKc_Zap-70 PTKc_Hck PTKc_Lyn PTKc_FGFR PTKc_Tec_Rlk PTKc_Csk PTKc_Axl_like PTKc_Lck_Blk PTKc_Tyk2_rpt2 PTKc_Fes PTKc_TrkC PTKc_DDR_like PTKc_Aatyk1_Aatyk3 PTKc_Btk_Bmx PTKc_Musk PTK_CCK4 PTK_Ryk PTKc_ALK_LTK PTKc_EphR_A10 PTK_HER3 no original description	2.4195967	0.03788858
Signalling	signalling.receptor kinases.leucine rich repeat XI	ciclev10010969m|pacid:20799210	highly similar to (755) AT3G47570 | Symbols: | leucine-rich repeat transmembrane protein kinase, putative | chr3:17527611-17530748 FORWARDmoderately similar to (369) RPK1_IPONI Receptor-like protein kinase precursor (EC 2.7.11.1) - Ipomoea nil (Japanese morning glory) (Pharbitis nil)highly similar to (724) loc_os12g42520 12012.m08031 protein receptor-like protein kinase precursor, putative S_TKc S_TKc Pkinase_Tyr Pkinase TyrKc PTKc PTKc_Csk_like PTKc_Srm_Brk PTKc_Frk_like PTKc_EphR PTKc_Src_like PTKc_Jak_rpt2 PTKc_Trk PTKc_Abl PTKc_Tec_like PTKc_Chk PTKc_Btk_Bmx PTKc_EphR_B PTKc_InsR_like SPS1 PTKc_Lck_Blk PTKc_Fyn_Yrk PTKc_Csk PTKc_Fes_like PTKc_TrkB PTKc_EphR_A2 PTKc_Src PTKc_EphR_A PTKc_TrkC PTKc_Tyk2_rpt2 PTKc_TrkA PTKc_Ror PTKc_Syk_like PTKc_EGFR_like PTKc_Itk PTKc_Fer PTKc_Yes PTKc_FGFR PTKc_Tec_Rlk PTKc_Jak1_rpt2 PTKc_Tie2 no original description	-99	9.86E-10
Signalling	signalling.receptor kinases.leucine rich repeat XI	ciclev10003409m|pacid:20787832	highly similar to (738) AT1G35710 | Symbols: | leucine-rich repeat transmembrane protein kinase, putative | chr1:13220940-13224386 FORWARDmoderately similar to (411) RPK1_IPONI Receptor-like protein kinase precursor (EC 2.7.11.1) - Ipomoea nil (Japanese morning glory) (Pharbitis nil)highly similar to (661) loc_os10g02970 12010.m03714 protein receptor-like protein kinase precursor, putative S_TKc S_TKc Pkinase TyrKc Pkinase_Tyr PTKc PTKc_Tec_like PTKc_Fes_like PTKc_Srm_Brk PTKc_EphR PTKc_Jak_rpt2 PTKc_Frk_like PTKc_Csk_like no original description	6.2512546	2.02E-20

Class	Familiy	GeneID	Description	log2(R/S)	pval
Signalling	signalling.receptor kinases.leucine rich repeat XI	ciclev10024240m|pacid:20807848	moderately similar to (339) AT4G08850 | Symbols: | kinase | chr4:5637467-5640496 REVERSEweakly similar to (182) PSKR_DAUCA Phytosulfokine receptor precursor (EC 2.7.11.1) (Phytosulfokine LRR receptor kinase) - Daucus carota (Carrot)moderately similar to (317) loc_os10g02970 12010.m03714 protein receptor-like protein kinase precursor, putative S_TKc Pkinase S_TKc TyrKc Pkinase_Tyr PTKc PTKc_Srm_Brk PTKc_Trk PTKc_EphR SPS1 PTKc_Tec_like PTKc_Fes_like PTKc_Csk_like PTKc_Src_like PTKc_Frk_like PTKc_Tec_Rlk PTKc_EphR_B no original description	-3.091349	0.0132453
Signalling	signalling.receptor kinases.leucine rich repeat XI	ciclev10030621m|pacid:20805683	highly similar to (579) AT4G08850 | Symbols: | kinase | chr4:5637467-5640496 REVERSEmoderately similar to (389) RPK1_IPONI Receptor-like protein kinase precursor (EC 2.7.11.1) - Ipomoea nil (Japanese morning glory) (Pharbitis nil)highly similar to (569) loc_os10g02970 12010.m03714 protein receptor-like protein kinase precursor, putative S_TKc S_TKc Pkinase Pkinase_Tyr TyrKc PTKc PTKc_EphR SPS1 PTKc_Csk_like PTKc_Tec_like PTKc_Jak_rpt2 PTKc_EGFR_like PTKc_Srm_Brk PTKc_EphR_B PTKc_Syk_like PTKc_Abl PTKc_Trk PTKc_Src_like PTKc_Ack_like PTKc_EphR_A2 PTKc_Btk_Bmx PTKc_Frk_like PTKc_Tec_Rlk PTKc_Fes_like PTK_CCK4 PTKc_Itk PTKc_EphR_A PTKc_Jak2_Jak3_rpt2 PTKc_Ror PTKc_Lck_Blk PTKc_Lyn PTKc_InsR_like PTKc_TrkA PTKc_Src PTKc_DDR PTKc_Fyn_Yrk PTKc_Fes PTKc_EGFR PTKc_Chk PTKc_c-ros PTKc_Hck PTKc_FAK PTKc_Fer PTKc_FGFR PTKc_ALK_LTK PTKc_TrkC PTKc_RET PTKc_Csk PTKc_Tyk2_rpt2 PTKc_Musk PTKc_Yes PTKc_HER2 no original description	-3.4017117	2.22E-07
Signalling	signalling.receptor kinases.leucine rich repeat XI	ciclev10027381m|pacid:20801390	highly similar to (515) AT3G47570 | Symbols: | leucine-rich repeat transmembrane protein kinase, putative | chr3:17527611-17530748 FORWARDmoderately similar to (310) RPK1_IPONI Receptor-like protein kinase precursor (EC 2.7.11.1) - Ipomoea nil (Japanese morning glory) (Pharbitis nil)highly similar to (539) loc_os02g01800 12002.m05530 protein receptor-like protein kinase precursor, putative S_TKc Pkinase_Tyr S_TKc TyrKc PTKc Pkinase PTKc_Srm_Brk PTKc_Src_like PTKc_Jak_rpt2 PTKc_Csk_like PTKc_Frk_like PTKc_Src PTKc_Abl PTKc_Fyn_Yrk PTKc_Tec_like PTKc_Fes_like SPS1 PTKc_InsR_like PTKc_Yes PTKc_Csk PTKc_Ror PTKc_EphR PTKc_Trk PTKc_Chk PTKc_Lck_Blk PTKc_Ack_like PTKc_EphR_A2 PTKc_Itk PTKc_EGFR_like PTKc_Tyk2_rpt2 PTKc_Syk_like PTKc_Btk_Bmx PTKc_EphR_B PTKc_Axl_like PTKc_EphR_A PTKc_c-ros PTKc_Lyn PTKc_Hck PTKc_Fes PTKc_Jak2_Jak3_rpt2 no original description	-2.971697	0.02121663

Class	Familiy	GeneID	Description	log2(R/S)	pval
Signalling	signalling.receptor kinases.leucine rich repeat XI	ciclev10000162m|pacid:20786057	moderately similar to (447) AT1G45616 | Symbols: AtRLP6 | AtRLP6 (Receptor Like Protein 6); protein binding | chr1:17183550-17186534 REVERSEmoderately similar to (217) RPK1_IPONI Receptor-like protein kinase precursor (EC 2.7.11.1) - Ipomoea nil (Japanese morning glory) (Pharbitis nil)moderately similar to (437) loc_os12g10870 12012.m05069 protein verticillium wilt disease resistance protein, putative no original description	2.4610476	0.02618876
Signalling	signalling.receptor kinases.leucine rich repeat XI	ciclev10025175m|pacid:20801462	moderately similar to (397) AT3G47570 | Symbols: | leucine-rich repeat transmembrane protein kinase, putative | chr3:17527611-17530748 FORWARDmoderately similar to (231) PSKR_DAUCA Phytosulfokine receptor precursor (EC 2.7.11.1) (Phytosulfokine LRR receptor kinase) - Daucus carota (Carrot)moderately similar to (462) loc_os01g49920 12001.m11177 protein receptor kinase-like protein, putative, expressed Pkinase_Tyr TyrKc S_TKc S_TKc Pkinase PTKc PTKc_Trk PTKc_Jak_rpt2 PTKc_Csk_like PTKc_Srm_Brk PTKc_Fes_like PTKc_Src_like PTKc_TrkC SPS1 PTKc_Frk_like PTKc_TrkA PTKc_Tec_like PTKc_Fer PTKc_Itk PTKc_Ror PTKc_EGFR_like PTKc_Chk PTKc_Csk PTKc_FGFR PTKc_TrkB PTKc_Jak2_Jak3_rpt2 PTKc_Abl PTKc_Src PTKc_Axl_like PTKc_Fes PTKc_FAK PTKc_Syk_like PTKc_Tyk2_rpt2 PTKc_EphR PTKc_EphR_A2 PTKc_Btk_Bmx PTKc_InsR_like PTKc_Lck_Blk PTKc_Tec_Rlk PTKc_Ack_like PTKc_Yes PTKc_Fyn_Yrk PTKc_Musk PTK_CCK4 PTKc_Tyro3 PTKc_c-ros PTKc_Ror1 PTKc_Lyn PTKc_DDR PTKc_EphR_A PTKc_Tie1 PTKc_EphR_B PTKc_Axl PTK_HER3 PTKc_Tie2 PTKc_Met_Ron PTKc_ALK_LTK PTKc_Jak1_rpt2 PTKc_HER4 no original description	1.8296369	0.08738275
Signalling	signalling.receptor kinases.leucine rich repeat XI	ciclev10033507m|pacid:20803361	moderately similar to (228) AT1G35710 | Symbols: | leucine-rich repeat transmembrane protein kinase, putative | chr1:13220940-13224386 FORWARDweakly similar to (196) RPK1_IPONI Receptor-like protein kinase precursor (EC 2.7.11.1) - Ipomoea nil (Japanese morning glory) (Pharbitis nil)moderately similar to (245) loc_os05g07740 12005.m05291 protein receptor-like protein kinase precursor, putative, expressed no original description	-4.070867	0.00103038
Signalling	signalling.receptor kinases.leucine rich repeat XI	ciclev10030752m|pacid:20803026	moderately similar to (464) AT3G47570 | Symbols: | leucine-rich repeat transmembrane protein kinase, putative | chr3:17527611-17530748 FORWARDmoderately similar to (241) RPK1_IPONI Receptor-like protein kinase precursor (EC 2.7.11.1) - Ipomoea nil (Japanese morning glory) (Pharbitis nil)highly similar to (513) loc_os02g12010 12002.m06449 protein receptor-like protein kinase precursor, putative no original description	-6.057374	4.36E-04

Class	Familiy	GeneID	Description	log2(R/S)	pval
Signalling	signalling.receptor kinases.leucine rich repeat XI	ciclev10033325m|pacid:20804207	moderately similar to (246) AT2G34930 | Symbols: | disease resistance family protein | chr2:14737169-14739886 REVERSEweakly similar to (127) PSKR_DAUCA Phytosulfokine receptor precursor (EC 2.7.11.1) (Phytosulfokine LRR receptor kinase) - Daucus carota (Carrot)moderately similar to (231) loc_os01g41750 12001.m10443 protein leucine-rich repeat receptor protein kinase EXS precursor, putative, expressed no original description	4.8978386	9.20E-02
Signalling	signalling.receptor kinases.leucine rich repeat XI	ciclev10025171m|pacid:20801501	moderately similar to (393) AT3G47570 | Symbols: | leucine-rich repeat transmembrane protein kinase, putative | chr3:17527611-17530748 FORWARDmoderately similar to (234) PSKR_DAUCA Phytosulfokine receptor precursor (EC 2.7.11.1) (Phytosulfokine LRR receptor kinase) - Daucus carota (Carrot)moderately similar to (461) loc_os01g49920 12001.m11177 protein receptor kinase-like protein, putative, expressed Pkinase_Tyr TyrKc S_TKc S_TKc Pkinase PTKc PTKc_Trk PTKc_Jak_rpt2 PTKc_Fes_like PTKc_Src_like PTKc_Srm_Brk PTKc_Csk_like PTKc_Frk_like PTKc_TrkC SPS1 PTKc_Tec_like PTKc_Ror PTKc_Itk PTKc_Fer PTKc_TrkA PTKc_Chk PTKc_FGFR PTKc_EGFR_like PTKc_Abl PTKc_TrkB PTKc_Src PTKc_Ror1 PTKc_Fes PTKc_EphR_A2 PTKc_Csk PTKc_Jak2_Jak3_rpt2 PTKc_Syk_like PTKc_InsR_like PTKc_Fyn_Yrk PTKc_Axl_like PTKc_Ack_like PTKc_FAK PTKc_Lck_Blk PTKc_Yes PTKc_c-ros PTKc_Tec_Rlk PTKc_EphR PTKc_Btk_Bmx PTKc_Tyk2_rpt2 PTKc_EphR_B PTKc_Tie1 PTKc_Musk PTKc_DDR PTKc_Lyn PTKc_Tie2 PTKc_Tyro3 PTK_CCK4 PTKc_EphR_A PTK_HER3 PTKc_ALK_LTK PTKc_Axl PTKc_Jak1_rpt2 PTKc_Met_Ron PTKc_Zap-70 PTKc_Ror2 PTKc_Tie PTKc_RET no original description	2.220121	3.82E-03
Signalling	signalling.receptor kinases.leucine rich repeat XI	ciclev10031896m|pacid:20804869	moderately similar to (426) AT3G20820 | Symbols: | leucine-rich repeat family protein | chr3:7280930-7282027 FORWARDweakly similar to (126) RPK1_IPONI Receptor-like protein kinase precursor (EC 2.7.11.1) - Ipomoea nil (Japanese morning glory) (Pharbitis nil)moderately similar to (390) loc_os08g39550 12008.m07936 protein polygalacturonase inhibitor 2 precursor, putative, expressed no original description	-1.7628148	3.76E-02

Class	Familiy	GeneID	Description	log2(R/S)	pval
Signalling	signalling.receptor kinases.leucine rich repeat XI	ciclev10033466m|pacid:20803311	moderately similar to (489) AT3G47570 | Symbols: | leucine-rich repeat transmembrane protein kinase, putative | chr3:17527611-17530748 FORWARDmoderately similar to (272) RPK1_IPONI Receptor-like protein kinase precursor (EC 2.7.11.1) - Ipomoea nil (Japanese morning glory) (Pharbitis nil)moderately similar to (486) loc_os06g16070 12006.m06320 protein receptor-like protein kinase precursor, putative Pkinase_Tyr S_TKc TyrKc Pkinase S_TKc PTKc PTKc_Trk PTKc_Csk_like PTKc_Jak_rpt2 PTKc_Fes_like PTKc_Srm_Brk PTKc_TrkC PTKc_TrkA PTKc_Src_like PTKc_Fer SPS1 PTKc_Ror PTKc_Frk_like PTKc_TrkB PTKc_FGFR PTKc_Tec_like PTKc_Ror1 PTKc_Fes PTKc_Itk PTKc_InsR_like PTKc_Syk_like PTKc_Csk PTKc_Ack_like PTKc_EphR PTKc_FAK PTKc_c-ros PTK_CCK4 PTKc_Jak2_Jak3_rpt2 PTKc_DDR PTKc_EGFR_like PTKc_EphR_A2 PTKc_Abl PTKc_Chk PTKc_Src PTKc_Axl_like PTKc_Tie2 PTKc_DDR_like PTKc_Fyn_Yrk PTKc_Met_Ron PTKc_Ror2 PTKc_Tie1 PTKc_Tec_Rlk PTKc_ALK_LTK PTKc_Musk PTKc_Tyro3 PTKc_Tyk2_rpt2 PTKc_Lck_Blk PTKc_Btk_Bmx PTKc_Yes PTKc_EphR_A no original description	4.6735115	4.39E-05
Signalling	signalling.receptor kinases.leucine rich repeat XI	ciclev10030536m|pacid:20803310	highly similar to (561) AT3G47570 | Symbols: | leucine-rich repeat transmembrane protein kinase, putative | chr3:17527611-17530748 FORWARDmoderately similar to (362) RPK1_IPONI Receptor-like protein kinase precursor (EC 2.7.11.1) - Ipomoea nil (Japanese morning glory) (Pharbitis nil)highly similar to (633) loc_os06g16070 12006.m06320 protein receptor-like protein kinase precursor, putative Pkinase_Tyr S_TKc Pkinase TyrKc S_TKc PTKc PTKc_Trk PTKc_Csk_like PTKc_Jak_rpt2 PTKc_Srm_Brk PTKc_Fes_like PTKc_TrkC PTKc_Src_like PTKc_TrkA PTKc_Ror PTKc_TrkB PTKc_Tec_like PTKc_Frk_like PTKc_Fer PTKc_Itk PTKc_Csk SPS1 PTKc_DDR PTKc_FAK PTKc_FGFR PTKc_Fes PTKc_Ack_like PTKc_Abl PTKc_InsR_like PTKc_Syk_like PTKc_EphR PTKc_Chk PTKc_Tec_Rlk PTKc_Ror1 PTKc_Jak2_Jak3_rpt2 PTKc_Ror2 PTKc_Src PTKc_c-ros PTKc_EGFR_like PTKc_Btk_Bmx PTKc_EphR_A2 PTKc_Met_Ron PTK_CCK4 PTKc_DDR_like PTKc_Fyn_Yrk PTKc_Axl_like PTKc_Tie1 PTKc_Musk no original description	3.8089938	3.33E-07

Class	Familiy	GeneID	Description	log2(R/S)	pval
Signalling	signalling.receptor kinases.leucine rich repeat XI	ciclev10029978m|pacid:20813974	highly similar to (684) AT4G08850 | Symbols: | kinase | chr4:5637467-5640496 REVERSEmoderately similar to (387) RPK1_IPONI Receptor-like protein kinase precursor (EC 2.7.11.1) - Ipomoea nil (Japanese morning glory) (Pharbitis nil)highly similar to (620) loc_os10g02970 12010.m03714 protein receptor-like protein kinase precursor, putative S_TKc Pkinase S_TKc Pkinase_Tyr TyrKc PTKc PTKc_EphR PTKc_Tec_like PTKc_Csk_like PTKc_Src_like PTKc_Srm_Brk PTKc_Fes_like PTKc_Btk_Bmx PTKc_Jak_rpt2 PTKc_Frk_like PTKc_Trk PTKc_EphR_A2 SPS1 PTKc_Tec_Rlk PTKc_Syk_like PTKc_EGFR_like PTKc_Ack_like PTKc_TrkC PTKc_Abl PTKc_EphR_B PTK_CCK4 PTKc_Itk PTKc_EphR_A PTKc_DDR PTKc_Fes PTKc_TrkA PTKc_Fyn_Yrk PTKc_Src PTKc_Tyk2_rpt2 PTKc_Ror PTKc_Jak2_Jak3_rpt2 PTKc_Jak1_rpt2 PTKc_TrkB PTKc_InsR_like PTKc_Csk PTKc_Chk PTKc_Yes PTKc_Fer PTKc_Lyn PTKc_Syk PTKc_Lck_Blk PTKc_FAK PTKc_c-ros no original description	2.1410694	0.0088359
Signalling	signalling.receptor kinases.leucine rich repeat XI	ciclev10025188m|pacid:20799799	moderately similar to (324) AT3G47570 | Symbols: | leucine-rich repeat transmembrane protein kinase, putative | chr3:17527611-17530748 FORWARDweakly similar to (148) RPK1_IPONI Receptor-like protein kinase precursor (EC 2.7.11.1) - Ipomoea nil (Japanese morning glory) (Pharbitis nil)moderately similar to (384) loc_os11g47210 12011.m08501 protein receptor-like protein kinase 5 precursor, putative Pkinase_Tyr S_TKc TyrKc S_TKc PTKc Pkinase PTKc_Csk_like PTKc_Trk PTKc_Src_like PTKc_Srm_Brk no original description	3.81068	2.58E-04
Signalling	signalling.receptor kinases.DUF 26	ciclev10023313m|pacid:20807107	moderately similar to (438) AT5G38260 | Symbols: | serine/threonine protein kinase, putative | chr5:15283692-15285837 REVERSEweakly similar to (176) KPRO_MAIZE Putative receptor protein kinase ZmPK1 precursor (EC 2.7.11.1) - Zea mays (Maize)moderately similar to (433) loc_os01g49614 12001.m43492 protein Ser/Thr protein kinase, putative, expressed Pkinase_Tyr TyrKc PTKc S_TKc Pkinase S_TKc PTKc_Src_like PTKc_Csk_like PTKc_Frk_like PTKc_Srm_Brk PTKc_EGFR_like PTKc_EphR PTKc_Tec_like SPS1 PTKc_Trk PTKc_Abl PTKc_Jak_rpt2 PTKc_Itk PTKc_Lck_Blk PTKc_Axl_like PTKc_Src PTKc_Fyn_Yrk PTKc_Yes PTKc_Syk_like PTKc_Fes_like PTKc_Chk PTKc_InsR_like PTKc_Tec_Rlk PTKc_Ror PTKc_Lyn PTKc_FGFR PTKc_Btk_Bmx PTKc_Axl PTKc_FGFR2 PTKc_Hck PTKc_Met_Ron PTKc_FGFR3 PTKc_EphR_A2 PTKc_c-ros PTKc_ALK_LTK PTKc_FGFR1 PTKc_EphR_B PTKc_Ack_like PTKc_Tie1 PTKc_InsR PTKc_Fer PTKc_EphR_A PTKc_EGFR PTKc_FGFR4 PTKc_TrkA PTKc_EphR_A10 PTKc_Fes PTKc_Jak2_Jak3_rpt2 PTKc_Tyro3 PTKc_HER4 PTKc_Tie2 PTK_HER3 PTKc_Syk PTKc_DDR2 PTKc_IGF-1R PTKc_Csk PTKc_DDR1 PTKc_DDR PTKc_DDR_like PTK_CCK4 no original description	-3.1647236	0.003659

Class	Familiy	GeneID	Description	log2(R/S)	pval
Signalling	signalling.receptor kinases.DUF 26	ciclev10027067m|pacid:20800943	moderately similar to (321) AT5G60900 | Symbols: RLK1 | RLK1 (RECEPTOR-LIKE PROTEIN KINASE 1); ATP binding / carbohydrate binding / kinase/ protein kinase/ protein serine/threonine kinase/ protein tyrosine kinase/ sugar binding | chr5:24498467-24501494 REVERSEmoderately similar to (232) KPRO_MAIZE Putative receptor protein kinase ZmPK1 precursor (EC 2.7.11.1) - Zea mays (Maize)moderately similar to (421) loc_os03g61310 12003.m11014 protein receptor-like protein kinase, putative, expressed Pkinase S_TKc S_TKc TyrKc Pkinase_Tyr PTKc PTKc_Src_like PTKc_Tec_like SPS1 PTKc_EphR PTKc_Frk_like PTKc_Jak_rpt2 PTKc_Csk_like PTKc_Lck_Blk PTKc_Itk PTKc_Srm_Brk PTKc_Fes_like PTKc_EphR_B PTKc_EphR_A PTKc_EphR_A2 PTKc_Chk PTKc_Syk_like PTKc_Abl PTKc_Lyn PTKc_Jak2_Jak3_rpt2 no original description	-4.7411947	1.91E-10
Signalling	signalling.receptor kinases.DUF 26	ciclev10010532m|pacid:20793692	highly similar to (577) AT1G65800 | Symbols: ARK2 | ARK2 (A. THALIANA RECEPTOR KINASE 2); kinase/ protein kinase/ transmembrane receptor protein serine/threonine kinase | chr1:24473166-24476523 FORWARDmoderately similar to (254) SLSG3_BRAOL S-locus-specific glycoprotein S13 precursor (SLSG-13) (Fragment) - Brassica oleracea (Wild cabbage)highly similar to (533) loc_os01g57560 12001.m11907 protein serine/threonine-protein kinase receptor precursor, putative, expressed Pkinase_Tyr PTKc TyrKc S_TKc Pkinase S_TKc PTKc_Jak_rpt2 B_lectin B_lectin B_lectin PTKc_Src_like PTKc_EphR PTKc_Trk PTKc_Srm_Brk PTKc_Csk_like PTKc_Axl_like PTKc_FGFR SPS1 PTKc_Fes_like PTKc_Tec_like PTKc_Syk_like PTKc_EphR_A2 PTKc_c-ros PTKc_Lck_Blk PTKc_Ror PTKc_Frk_like PTKc_ALK_LTK PTKc_InsR_like PTKc_EGFR_like PTKc_Chk PTKc_Ack_like PTKc_EphR_A PTKc_Tyk2_rpt2 PTKc_Lyn PTKc_Src PTKc_Btk_Bmx PAN_2 PTK_CCK4 PTKc_Musk PTKc_DDR PTKc_EphR_B PTKc_Itk PTKc_Axl PTKc_Tyro3 PTKc_Fyn_Yrk PTKc_Tie2 PTKc_Csk PTKc_RET PTKc_TrkB PTK_Ryk PTKc_Jak2_Jak3_rpt2 PTKc_DDR2 PTKc_Abl PTKc_FGFR4 PTKc_TrkA PTKc_Fer PTKc_Yes PTKc_Ror2 PTKc_Hck PTKc_DDR1 no original description	2.9355912	0.0132453

Class	Familiy	GeneID	Description	log2(R/S)	pval
Signalling	signalling.receptor kinases.DUF 26	ciclev10007028m|pacid:20791925	moderately similar to (369) AT5G60900 | Symbols: RLK1 | RLK1 (RECEPTOR-LIKE PROTEIN KINASE 1); ATP binding / carbohydrate binding / kinase/ protein kinase/ protein serine/threonine kinase/ protein tyrosine kinase/ sugar binding | chr5:24498467-24501494 REVERSEmoderately similar to (272) KPRO_MAIZE Putative receptor protein kinase ZmPK1 precursor (EC 2.7.11.1) - Zea mays (Maize)moderately similar to (389) loc_os04g12600 12004.m06503 protein receptor-like protein kinase, putative TyrKc Pkinase_Tyr Pkinase S_TKc S_TKc PTKc PTKc_EphR PTKc_Csk_like PTKc_Srm_Brk SPS1 PTKc_Syk_like PTKc_Src_like PTKc_Jak_rpt2 PTKc_EphR_A2 PTKc_InsR_like PTKc_Frk_like PTKc_EGFR_like PTKc_FGFR PTKc_Fes_like PTKc_Trk PTKc_c-ros PTKc_ALK_LTK PTKc_Itk PTKc_Abl PTKc_Btk_Bmx PTKc_Zap-70 PTKc_Met_Ron PTKc_Tec_like PTKc_EphR_B PTKc_EphR_A PTKc_Chk PTKc_Syk PTKc_Fer PTKc_Ack_like PTKc_Ror PTKc_Jak2_Jak3_rpt2 PTKc_DDR PTKc_Tie2 PTKc_Fes PTKc_HER4 PTKc_Tec_Rlk PTKc_FAK PTKc_Csk PTKc_Lyn PTKc_EGFR PTKc_TrkB PTKc_Tie1 PTKc_DDR_like PTKc_Axl_like PTKc_TrkA PTKc_Musk PTKc_Tyk2_rpt2 PTKc_RET PTK_CCK4 PTKc_EphR_A10 PTKc_Tie PTKc_Fyn_Yrk PTKc_InsR PTKc_TrkC PTKc_Lck_Blk no original description	-3.72352	0.07544367
Signalling	signalling.receptor kinases.DUF 26	ciclev10013919m|pacid:20796516	weakly similar to (122) AT3G09010 | Symbols: | protein kinase family protein | chr3:2750285-2752086 FORWARDvery weakly similar to (85.5) CRI4_MAIZE Putative receptor protein kinase CRINKLY4 precursor (EC 2.7.11.1) - Zea mays (Maize)weakly similar to (141) loc_os08g10070 12008.m26611 protein serine/threonine-protein kinase receptor precursor, putative, expressed no original description	1.8645616	0.03087966
Signalling	signalling.receptor kinases.DUF 26	ciclev10016248m|pacid:20817899	moderately similar to (207) AT3G14840 | Symbols: | leucine-rich repeat family protein / protein kinase family protein | chr3:4988271-4993891 FORWARDweakly similar to (107) NORK_MEDTR Nodulation receptor kinase precursor (EC 2.7.11.1) (Does not make infections protein 2) (Symbiosis receptor-like kinase) (MtSYMRK) - Medicago truncatula (Barrel medic)weakly similar to (197) loc_os09g17630 12009.m05021 protein receptor-like protein kinase 2, putative, expressed PTKc TyrKc Pkinase_Tyr S_TKc Pkinase S_TKc PTKc_Csk_like no original description	3.2091112	7.51E-03

Class	Familiy	GeneID	Description	log2(R/S)	pval
Signalling	signalling.receptor kinases.DUF 26	ciclev10020672m|pacid:20810014	moderately similar to (267) AT1G53430 | Symbols: | leucine-rich repeat family protein / protein kinase family protein | chr1:19936073-19940959 FORWARDweakly similar to (127) NORK_MEDTR Nodulation receptor kinase precursor (EC 2.7.11.1) (Does not make infections protein 2) (Symbiosis receptor-like kinase) (MtSYMRK) - Medicago truncatula (Barrel medic)moderately similar to (279) loc_os09g17630 12009.m05021 protein receptor-like protein kinase 2, putative, expressed Pkinase S_TKc PTKc TyrKc Pkinase_Tyr S_TKc PTKc_EphR PTKc_Jak_rpt2 PTKc_Csk_like PTKc_Srm_Brk SPS1 PTKc_EGFR_like PTKc_EphR_A2 PTKc_Ror no original description	1.5570484	0.06877779
Signalling	signalling.receptor kinases.DUF 26	ciclev10011232m|pacid:20798864	moderately similar to (471) AT5G38260 | Symbols: | serine/threonine protein kinase, putative | chr5:15283692-15285837 REVERSEweakly similar to (179) KPRO_MAIZE Putative receptor protein kinase ZmPK1 precursor (EC 2.7.11.1) - Zea mays (Maize)moderately similar to (418) loc_os01g49614 12001.m43492 protein Ser/Thr protein kinase, putative, expressed TyrKc Pkinase_Tyr Pkinase PTKc S_TKc S_TKc PTKc_Src_like PTKc_Tec_like PTKc_Jak_rpt2 PTKc_Csk_like PTKc_EGFR_like PTKc_Abl PTKc_Itk PTKc_Srm_Brk SPS1 PTKc_Frk_like PTKc_Syk_like PTKc_Lck_Blk PTKc_EphR PTKc_Trk PTKc_Ack_like PTKc_FGFR PTKc_Ror PTKc_Fyn_Yrk PTKc_Lyn PTKc_Axl_like PTKc_Chk PTKc_Fes_like PTKc_Tec_Rlk PTKc_Src PTKc_Yes PTKc_Hck PTKc_Jak2_Jak3_rpt2 PTKc_ALK_LTK PTKc_HER4 PTKc_InsR_like PTKc_Btk_Bmx PTKc_c-ros PTKc_EphR_B PTKc_EphR_A2 PTKc_Ror1 PTKc_HER2 PTKc_Tie2 PTKc_Fer PTKc_DDR PTKc_TrkA PTK_CCK4 PTKc_EphR_A PTKc_Fes PTKc_Musk PTKc_EGFR PTKc_Axl PTKc_FAK PTK_HER3 PTKc_InsR PTKc_RET PTKc_Tyro3 PTKc_TrkC PTKc_IGF-1R PTKc_FGFR3 PTKc_Ror2 PTKc_FGFR2 PTKc_Met_Ron PTKc_EphR_A10 PTKc_TrkB PTKc_DDR1 PTKc_FGFR1 PTKc_FGFR4 PTKc_PDGFR PTKc_DDR_like PTKc_Csk PTKc_Syk PTKc_Aatyk1_Aatyk3 PTKc_DDR2 PTKc_Tyk2_rpt2 no original description	1.8236805	9.34E-02

Class	Familiy	GeneID	Description	log2(R/S)	pval
Signalling	signalling.receptor kinases.DUF 26	ciclev10030238m|pacid:20812879	moderately similar to (349) AT4G11530 | Symbols: | kinase | chr4:6987093-6989599 FORWARDmoderately similar to (204) PSKR_DAUCA Phytosulfokine receptor precursor (EC 2.7.11.1) (Phytosulfokine LRR receptor kinase) - Daucus carota (Carrot)moderately similar to (365) loc_os01g57480 12001.m11899 protein serine/threonine-protein kinase receptor precursor, putative, expressed Glyco_18 Glyco_hydro_18 Pkinase_Tyr TyrKc PTKc S_TKc S_TKc Pkinase PTKc_Srm_Brk PTKc_Src_like PTKc_Jak_rpt2 PTKc_Frk_like PTKc_Csk_like PTKc_Tec_like PTKc_Abl PTKc_EGFR_like PTKc_ALK_LTK PTKc_InsR_like PTKc_Fes_like PTKc_Lck_Blk PTKc_Ror PTKc_c-ros PTKc_EphR PTKc_Trk PTKc_Syk_like PTKc_Itk PTKc_Lyn PTKc_Jak2_Jak3_rpt2 PTKc_Musk SPS1 PTKc_TrkA PTKc_Ack_like PTKc_Fyn_Yrk PTKc_DDR PTKc_RET PTKc_IGF-1R PTKc_TrkB PTK_CCK4 PTKc_Axl_like PTKc_InsR PTKc_Yes PTKc_TrkC PTKc_Hck PTKc_Ror1 ChiA PTKc_Src PTKc_Tec_Rlk PTKc_FGFR PTKc_Ror2 PTKc_Chk PTKc_EphR_A2 PTKc_Csk PTKc_Btk_Bmx PTKc_EphR_A PTKc_HER4 PTKc_DDR1 PTKc_EphR_B PTKc_Axl PTKc_DDR2 PTKc_DDR_like PTKc_EGFR PTKc_Fer PTK_HER3 PTKc_PDGFR PTKc_Met_Ron PTKc_Tie2 PTKc_Tyk2_rpt2 no original description	3.77683	1.51E-02
Signalling	signalling.receptor kinases.DUF 26	ciclev10018902m|pacid:20806016	highly similar to (583) AT5G60900 | Symbols: RLK1 | RLK1 (RECEPTOR-LIKE PROTEIN KINASE 1); ATP binding / carbohydrate binding / kinase/ protein kinase/ protein serine/threonine kinase/ protein tyrosine kinase/ sugar binding | chr5:24498467-24501494 REVERSEmoderately similar to (309) KPRO_MAIZE Putative receptor protein kinase ZmPK1 precursor (EC 2.7.11.1) - Zea mays (Maize)highly similar to (665) loc_os04g12560 12004.m06499 protein receptor-like protein kinase, putative, expressed S_TKc Pkinase S_TKc Pkinase_Tyr TyrKc PTKc PTKc_Csk_like PTKc_InsR_like PTKc_Itk PTKc_Chk PTKc_Jak_rpt2 PTKc_InsR PTKc_Tec_like SPS1 PTKc_Srm_Brk PTKc_Syk_like PTKc_EphR PTKc_Ror PTKc_Src_like PTKc_IGF-1R PTKc_Trk PTKc_Frk_like PTKc_Btk_Bmx PTKc_ALK_LTK PTKc_Abl PTKc_Fes_like PTKc_Tec_Rlk PTKc_Jak2_Jak3_rpt2 PTKc_EphR_A2 PTKc_Csk PTKc_DDR PTKc_Axl_like PTKc_FGFR PTKc_Ack_like PTKc_TrkA PTKc_Met_Ron PTKc_EphR_B PTKc_EGFR_like PTKc_c-ros PTKc_Musk PTKc_Syk PTKc_PDGFR PTKc_Fer PTKc_EphR_A PTK_CCK4 PTKc_Fes PTKc_Tie2 PTKc_Zap-70 PTKc_Aatyk1_Aatyk3 PTKc_Aatyk PTKc_Tyk2_rpt2 PTKc_Ror1 PTKc_RET PTKc_Lck_Blk no original description	2.6319273	9.58E-03

Class	Familiy	GeneID	Description	log2(R/S)	pval
Signalling	signalling.receptor kinases.DUF 26	ciclev10011753m|pacid:20797932	moderately similar to (365) AT1G70250 | Symbols: | receptor serine/threonine kinase, putative | chr1:26452975-26456088 FORWARDweakly similar to (179) KPRO_MAIZE Putative receptor protein kinase ZmPK1 precursor (EC 2.7.11.1) - Zea mays (Maize)moderately similar to (374) loc_os01g49580 12001.m11145 protein protein kinase domain containing protein, expressed Pkinase S_TKc TyrKc Pkinase_Tyr PTKc S_TKc PTKc_Csk_like PTKc_Jak_rpt2 PTKc_Abl PTKc_Tec_like PTKc_Src_like SPS1 PTKc_Itk PTKc_Fes_like PTKc_EGFR_like PTKc_EphR PTKc_FGFR PTKc_Ror PTKc_Srm_Brk PTKc_Trk PTKc_Syk_like PTKc_InsR_like PTKc_Lck_Blk PTKc_Ack_like PTKc_Axl_like PTKc_Btk_Bmx PTKc_Chk PTKc_EphR_A2 PTKc_Fyn_Yrk PTKc_Tec_Rlk PTKc_Lyn PTKc_Src PTKc_EphR_B PTKc_Jak2_Jak3_rpt2 PTKc_Frk_like PTKc_Fes PTKc_ALK_LTK PTKc_c-ros PTK_CCK4 PTKc_Yes PTKc_Fer PTKc_HER4 PTKc_DDR PTKc_Tie2 PTKc_Musk PTKc_InsR PTKc_Hck PTKc_Ror2 PTK_HER3 PTKc_Csk PTKc_EphR_A PTKc_Axl PTKc_HER2 PTKc_DDR1 PTKc_TrkA PTKc_EGFR PTKc_IGF-1R PTKc_FGFR3 PTKc_TrkB no original description	3.489907	9.99E-02
Signalling	signalling.receptor kinases.DUF 26	ciclev10014567m|pacid:20818017	moderately similar to (403) AT5G38260 | Symbols: | serine/threonine protein kinase, putative | chr5:15283692-15285837 REVERSEweakly similar to (166) KPRO_MAIZE Putative receptor protein kinase ZmPK1 precursor (EC 2.7.11.1) - Zea mays (Maize)moderately similar to (422) loc_os01g49580 12001.m11145 protein protein kinase domain containing protein, expressed TyrKc PTKc Pkinase_Tyr S_TKc Pkinase S_TKc PTKc_Tec_like PTKc_Jak_rpt2 PTKc_Src_like PTKc_Itk PTKc_Csk_like PTKc_FGFR PTKc_Srm_Brk PTKc_Abl PTKc_EGFR_like PTKc_EphR PTKc_Tec_Rlk PTKc_Syk_like PTKc_Trk SPS1 PTKc_Fes_like PTKc_Ror PTKc_Axl_like PTKc_Btk_Bmx PTKc_Frk_like PTKc_ALK_LTK PTKc_Lck_Blk PTKc_FGFR3 PTKc_Chk PTKc_InsR_like PTKc_HER4 PTKc_FGFR2 PTKc_Ack_like PTKc_Tie2 PTKc_Fes PTKc_Src PTKc_FGFR1 PTKc_Fer PTKc_Axl PTKc_Lyn PTKc_Yes PTKc_FGFR4 PTKc_Fyn_Yrk PTKc_Met_Ron PTKc_EphR_A2 PTKc_Tie1 PTKc_c-ros PTK_CCK4 PTKc_EphR_B PTKc_RET PTKc_Tie PTKc_EphR_A PTKc_Hck PTKc_InsR PTKc_Ror2 PTKc_Jak2_Jak3_rpt2 PTKc_Ror1 PTK_HER3 no original description	-4.5669	1.66E-09
Signalling	signalling.receptor kinases.misc	ciclev10023778m|pacid:20809452	weakly similar to (180) AT3G46280 | Symbols: | protein kinase-related | chr3:17005672-17008410 REVERSEweakly similar to (171) loc_os01g03370 12001.m06976 protein senescence-induced receptor-like serine/threonine-protein kinase precursor, putative no original description	99	1.08E-15

Class	Familiy	GeneID	Description	log2(R/S)	pval
Signalling	signalling.calcium	ciclev10012776m|pacid:20797948	weakly similar to (194) AT4G20780 | Symbols: | calcium-binding protein, putative | chr4:11133309-11133884 REVERSEmoderately similar to (204) ALLB3_BETVE Calcium-binding allergen Bet v 3 (Bet v III) - Betula verrucosa (White birch) (Betula pendula)weakly similar to (161) loc_os03g21380 12003.m35206 protein calcium-binding protein CAST, putative, expressed no original description	-2.2135084	8.47E-04
Signalling	signalling.calcium	ciclev10010422m|pacid:20795887	weakly similar to (115) AT4G27280 | Symbols: | calcium-binding EF hand family protein | chr4:13663770-13664162 REVERSEweakly similar to (108) loc_os01g57470 12001.m11898 protein caltractin, putative, expressed no original description	1.7359078	2.42E-02
Signalling	signalling.calcium	ciclev10011627m|pacid:20798960	highly similar to (560) AT3G52870 | Symbols: | calmodulin-binding family protein | chr3:19593365-19595686 REVERSEhighly similar to (512) loc_os12g05420 12012.m04535 protein calmodulin binding protein, putative, expressed no original description	-1.715192	0.00982231
Signalling	signalling.calcium	ciclev10029360m|pacid:20814504	moderately similar to (220) AT2G15680 | Symbols: | calmodulin-related protein, putative | chr2:6831024-6831587 FORWARDvery weakly similar to (89.4) CALM_CHLRE Calmodulin (CaM) - Chlamydomonas reinhardtiiweakly similar to (110) loc_os05g13580 12005.m05773 protein calmodulin-related protein 2, touch-induced, putative, expressed FRQ1 no original description	2.19992	0.00110858
Signalling	signalling.G-proteins	ciclev10032726m|pacid:20802915	weakly similar to (181) AT5G05380 | Symbols: PRA1.B3 | PRA1.B3 (PRENYLATED RAB ACCEPTOR 1.B3) | chr5:1592214-1592867 FORWARDweakly similar to (147) loc_os05g39670 12005.m08145 protein prenylated Rab receptor 2, putative, expressed PRA1 no original description	-1.5546261	0.03990679
Signalling	signalling.G-proteins	ciclev10030874m|pacid:20804320	highly similar to (875) AT5G61980 | Symbols: AGD1 | AGD1 (ARF-GAP domain 1); ARF GTPase activator/ protein binding / zinc ion binding | chr5:24894472-24899178 FORWARDhighly similar to (760) loc_os09g33600 12009.m06409 protein VAN3, putative, expressed ArfGap ArfGap COG5347 PH_centaurin no original description	1.5924369	0.04814994
Signalling	signalling.phosphorelay	ciclev10006135m|pacid:20791051	moderately similar to (202) AT3G21510 | Symbols: AHP1 | AHP1 (HISTIDINE-CONTAINING PHOSPHOTRANSMITTER 1); histidine phosphotransfer kinase | chr3:7578432-7579537 REVERSEweakly similar to (114) HP1_ORYSA Histidine-containing phosphotransfer protein 1 (OsHP1) - Oryza sativa (Rice)weakly similar to (129) loc_os05g44570 12005.m64253 protein histidine-containing phosphotransfer protein 4, putative, expressed no original description	1.8480601	0.01859643
Signalling	signalling.light	ciclev10031240m|pacid:20803230	highly similar to (572) AT5G03250 | Symbols: | phototropic-responsive NPH3 family protein | chr5:774591-776855 FORWARDmoderately similar to (258) NPH3_ORYSA Coleoptile phototropism protein 1 (Non-phototropic hypocotyl 3-like protein) (NPH3-like protein) - Oryza sativa (Rice)highly similar to (504) loc_os03g43990 12003.m35330 protein transposon protein, putative, Mutator sub-class, expressedNPH3 no original description	-2.0898242	0.05633026

Class	Familiy	GeneID	Description	log2(R/S)	pval
Signalling	signalling.light	ciclev10013892m|pacid:20796657	moderately similar to (298) AT4G38180 | Symbols: FRS5 | FRS5 (FAR1-related sequence 5); zinc ion binding | chr4:17906702-17909404 REVERSEmoderately similar to (345) loc_os04g25100 12004.m07640 protein transposon protein, putative, unclassified no original description	-8.669809	2.76E-12
Peroxidase	misc.peroxidases	ciclev10015924m|pacid:20817540	moderately similar to (431) AT3G21770 | Symbols: | peroxidase 30 (PER30) (P30) (PRXR9) | chr3:7673345-7674661 FORWARDmoderately similar to (332) PER1_ORYSA Peroxidase 1 precursor (EC 1.11.1.7) - Oryza sativa (Rice)moderately similar to (378) loc_os06g46799 12006.m09205 protein peroxidase 39 precursor, putative, expressedsecretory_peroxidase plant_peroxidase peroxidase no original description	-1.4891722	4.57E-02
Peroxidase	misc.peroxidases	ciclev10012179m|pacid:20796807	moderately similar to (447) AT5G40150 | Symbols: | peroxidase, putative | chr5:16059750-16060736 REVERSEmoderately similar to (218) PER2_ARAHY Cationic peroxidase 2 precursor (EC 1.11.1.7) (PNPC2) - Arachis hypogaea (Peanut)moderately similar to (363) loc_os02g50770 12002.m10111 protein peroxidase 65 precursor, putative, expressedsecretory_peroxidase plant_peroxidase peroxidase ascorbate_peroxidase no original description	-1.5760288	0.02637428
Glutathione-S-transferase	misc.glutathione S transferases	ciclev10006981m|pacid:20790505	weakly similar to (144) AT3G09270 | Symbols: ATGSTU8 | ATGSTU8 (GLUTATHIONE S-TRANSFERASE TAU 8); glutathione transferase | chr3:2848407-2849226 REVERSEweakly similar to (172) GSTX3_TOBAC Probable glutathione S-transferase (EC 2.5.1.18) (Auxin-induced protein PCNT103) - Nicotiana tabacum (Common tobacco)weakly similar to (151) loc_os09g29200 12009.m06071 protein glutathione S-transferase, putative, expressed GST_N_Tau GST_C_Tau Gst no original description	-99	0.00387935
Glutathione-S-transferase	misc.glutathione S transferases	ciclev10012511m|pacid:20797745	moderately similar to (309) AT5G02790 | Symbols: | In2-1 protein, putative | chr5:632877-634858 FORWARDmoderately similar to (252) IN21_MAIZE IN2-1 protein - Zea mays (Maize)moderately similar to (286) loc_os03g17470 12003.m07174 protein IN2-1 protein, putative, expressed GST_C_Lambda no original description	3.820249	4.66E-02
Glutathione-S-transferase	misc.glutathione S transferases	ciclev10006839m|pacid:20790572	weakly similar to (127) AT1G74590 | Symbols: ATGSTU10, GSTU10 | GSTU10 (GLUTATHIONE S-TRANSFERASE TAU 10); glutathione transferase | chr1:28023887-28024666 REVERSEweakly similar to (138) GSTX3_TOBAC Probable glutathione S-transferase (EC 2.5.1.18) (Auxin-induced protein PCNT103) - Nicotiana tabacum (Common tobacco)weakly similar to (130) loc_os09g29200 12009.m06071 protein glutathione S-transferase, putative, expressed GST_N_Tau no original description	-7.043616	3.07E-11

Class	Familiy	GeneID	Description	log2(R/S)	pval
Glutathione-S-transferase	misc.glutathione S transferases	ciclev10012621m|pacid:20797419	moderately similar to (293) AT5G02790 | Symbols: | In2-1 protein, putative | chr5:632877-634858 FORWARDmoderately similar to (246) IN21_MAIZE IN2-1 protein - Zea mays (Maize)moderately similar to (285) loc_os03g17470 12003.m07174 protein IN2-1 protein, putative, expressed GST_C_Lambda no original description	3.820249	4.66E-02
Glutathione-S-transferase	misc.glutathione S transferases	ciclev10032702m|pacid:20802483	weakly similar to (163) AT2G29420 | Symbols: ATGSTU7, GST25 | ATGSTU7 (ARABIDOPSIS THALIANA GLUTATHIONE S-TRANSFERASE TAU 7); glutathione transferase | chr2:12618111-12618871 REVERSEweakly similar to (166) GSTX1_TOBAC Probable glutathione S-transferase (EC 2.5.1.18) (Auxin-induced protein PGNT1/PCNT110) - Nicotiana tabacum (Common tobacco)weakly similar to (161) loc_os09g29200 12009.m06071 protein glutathione S-transferase, putative, expressed GST_C_Tau GST_N_Tau Gst no original description	2.7584672	1.05E-05
Abiotic stress	stress.abiotic.heat	ciclev10033341m|pacid:20803150	highly similar to (617) AT5G02500 | Symbols: HSC70-1, HSP70-1, AT-HSC70-1, HSC70 | HSC70-1 (HEAT SHOCK COGNATE PROTEIN 70-1); ATP binding | chr5:554055-556334 REVERSEhighly similar to (620) HSP7C_PETHY Heat shock cognate 70 kDa protein - Petunia hybrida (Petunia)highly similar to (628) loc_os11g47760 12011.m080070 protein heat shock cognate 70 kDa protein 2, putative, expressed HSP70 dnaK DnaK dnaK hscA hscA no original description	3.5518293	4.25E-08
Abiotic stress	stress.abiotic.heat	ciclev10015130m|pacid:20817387	moderately similar to (295) AT5G09590 | Symbols: MTHSC70-2, HSC70-5 | MTHSC70-2 (MITOCHONDRIAL HSP70 2); ATP binding | chr5:2975721-2978508 FORWARDmoderately similar to (310) HSP7M_PHAVU Heat shock 70 kDa protein, mitochondrial precursor - Phaseolus vulgaris (Kidney bean) (French bean)moderately similar to (305) loc_os03g02260 12003.m05768 protein heat shock 70 kDa protein, mitochondrial precursor, putative, expressed dnaK HSP70 DnaK dnaK hscA hscA no original description	-8.796031	1.07E-14
Abiotic stress	stress.abiotic.drought/salt	ciclev10030080m|pacid:20813170	weakly similar to (105) AT2G34300 | Symbols: | dehydration-responsive protein-related | chr2:14473916-14476811 REVERSEvery weakly similar to (87.8) loc_os01g66110 12001.m42777 protein ankyrin-like protein, putative, expressed no original description	1.7313106	0.0420184
Abiotic stress	stress.abiotic.drought/salt	ciclev10000463m|pacid:20789212	nearly identical (1042) AT1G78240 | Symbols: TSD2, QUA2 | TSD2 (TUMOROUS SHOOT DEVELOPMENT 2); methyltransferase | chr1:29433173-29435815 REVERSEhighly similar to (836) loc_os02g51860 12002.m10217 protein ATP binding protein, putative, expressedDUF248 no original description	1.4871604	4.96E-02
Abiotic stress	stress.abiotic.drought/salt	ciclev10024532m|pacid:20810943	nearly identical (1037) AT4G02900 | Symbols: | early-responsive to dehydration protein-related / ERD protein-related | chr4:1284066-1287747 FORWARDnearly identical (1011) loc_os05g32720 12005.m07505 protein ERD4 protein, putative, expressed DUF221 COG5594 no original description	-2.990212	6.54E-05

Class	Familiy	GeneID	Description	log2(R/S)	pval
Abiotic stress	stress.abiotic.unspecified	ciclev10026481m|pacid:20800527	moderately similar to (254) AT1G72610 | Symbols: GLP1, ATGER1, GER1 | GER1 (GERMIN-LIKE PROTEIN 1); oxalate oxidase | chr1:27339302-27339928 REVERSEmoderately similar to (258) AB19A_PRUPE Auxin-binding protein ABP19a precursor - Prunus persica (Peach)moderately similar to (246) loc_os08g35760 12008.m07565 protein auxin-binding protein ABP20 precursor, putative, expressedCupin_1 no original description	-1.518158	5.22E-02
