# Supplementary material for: Transcriptome Profiling of Huanglongbing (HLB) Tolerant and Susceptible Citrus Plants Reveals the Role of Basal Resistance in HLB Tolerance
Source: Front Plant Sci. 2016 Jun 28;7:933. doi: 10.3389/fpls.2016.00933 (PMC4923198; doi:10.3389/fpls.2016.00933)
Supplement: Table S9 — DE genes validated using real time PCR. [file Table9.PDF]

Table S9 Validating DE genes using real time PCR

	Relative gene expression based on RT-PCR (2-ΔΔCt)		
GENE ID	the mean value of the tolerant citrus 'Jackson' (R20T18 and R20T17) compared with the susceptible citrus 'Marsh'(R19T24 and R20T24)		the mean value of he tolerant citrus 'Jackson' (R20T09 and R20T10) compared with thesusceptible citrus 'Marsh'(R20T07 and R20T08)
up-regulated genes			
XLOC-007465	18.99457307		6.233316637
XLOC-008688	48.87902071		3.073750363
XLOC-009459	Inf		Inf
XLOC-016038	41.54227534		5.337817572
XLOC-017605	Inf		Inf
XLOC-017905	4.886650687		30.64330498
XLOC-020552	15.17571556		3.512504321
XLOC-010182	0.871305165		4.515857619
XLOC-012787	3.369419364		2.049113646
XLOC-017358	16.30791006		3.482202253
XLOC-024919	0.10330629		0.375009747
XLOC-027134	0.52168469		16.50693087
XLOC-032747	82.28171733		349.1008356
XLOC-010254	235.1601837		29.96071192
down-regualted genes			
XLOC-002746	0.356959899		0.896577376
XLOC-006005	-Inf		0.278114332
XLOC-017588	0.067472507		0.031576608
XLOC-025084	0.56254106		0.19144475
XLOC-026805	0.26413187		0.160428237
XLOC-033010	0.463641643		1.068065408
XLOC-010349	0.189847995		0.06640796
XLOC-024934	0.457840397		0.412509715
XLOC-026417	-Inf		-Inf
XLOC-026696	-Inf		-Inf
XLOC-036620	-Inf		1.233279674
XLOC-031655	0.006053912		0.048445283
XLOC-008025	-Inf		1.224760789
XLOC-000748	-Inf		0.076547549
XLOC-004058	0.002859542		0.07419676
XLOC-005869	0.771105413		2.103074562
XLOC-006868	0.027489034		-Inf
XLOC-019674	-Inf		-Inf
XLOC-033108	0.048361406		0.723216752

gene expression was normalized to expression of GADPH and is presened as mean value of the tolarant citrus 'Jackson' campared to the susceptible citrus 'Marsh'. The The value >1 means up-regulated. The value <1 means down-regulated.

inf means the Ct value was not detected 'Marsh' samples but detected in 'Jackson' samples within 40 cycles of RT-PCR.
 -inf means the Ct value was detected 'Marsh' samples but not detected in 'Jackson' samples within 40 cycles of RT-PCR.
